# Enantiospecific deoxyfluorination of cyclic α-OH-β-ketoesters†

**DOI:** 10.1039/d0ob02152k

**Published:** 2021-01-06

**Authors:** Christopher Mairhofer, Victoria Haider, Thomas Bögl, Mario Waser

**Affiliations:** a Johannes Kepler University Linz, Institute of Organic Chemistry, Altenbergerstraße 69, 4040 Linz, Austria; b Johannes Kepler University Linz, Institute of Analytical Chemistry, Altenbergerstraße 69, 4040 Linz, Austria

## Abstract

We herein report the deoxyfluorination of cyclic α-hydroxy-β-ketoesters using diethylaminosulfur trifluoride (DAST). The reaction proceeds with excellent levels of stereospecificity, giving the configurationally inverted α-fluoro-β-ketoesters in high yields under operationally simple conditions.

The deoxyfluorination of alcohol derivatives is one of the most commonly applied methods to access organofluorine compounds.^1,2^ A broad variety of complementary strategies for the deoxyfluorination of C_sp2_–OH and C_sp3_–OH functionalities by making use of different, nowadays often commercially available, reagents have been reported over the last few decades.^1–6^ The most prominently used agent for this purpose is diethylaminosulfur trifluoride (DAST, **1**), which acts by simultaneously activating the hydroxyl group while delivering a nucleophilic fluoride anion.^3–5^ Besides, the last years have seen significant progress in the development of alternative (sometimes less sensitive) and maybe cheaper reagents,^6^ as illustrated in *e.g.* a very recent report using CuF_2_ in combination with Lewis bases.^6*g*^

Over the last years our group has focused on the stereoselective synthesis of α-hydroxylated and α-fluorinated β-ketoesters **2** and **3** (starting from β-ketoesters **4**) by using bifunctional chiral quaternary ammonium salt catalysts.^7,8^ Within these studies, we generally achieved higher enantio-selectivities for the α-hydroxylated products **2** ^8^ than for the α-fluorinated **3**.^7^ We thus wondered, if it may be possible to convert the alcohols **2** into organofluorine compounds **3** by means of a stereospecific deoxyfluorination process (Scheme 1). However, and surprising to us, despite of all the achievements in the field of (stereospecific) deoxyfluorinations of alcohols, α-hydroxy-carbonyl substrates have been sparingly used only.^9,10^ When looking at these previous reports it becomes obvious that the main obstacle, when targeting the conversion of **2** into **3**, will be the suppression of the deoxygenation and difluorination of the carbonyl-group. However, by looking at some of the earlier reports, it may sound feasible that carefully balanced reaction conditions (temperature, order of addition of the reagents, …) may be fruitful to carry out the deoxyfluorination of compounds **2** without touching the carbonyl group, thus filling this long-standing application gap in the toolbox of enantiospecific deoxyfluorination reactions.

We started our investigations by optimizing the deoxyfluorination of enantioenriched (*S*)-**2a** (obtained as reported previously^8^) with DAST (**1**), one of the cheapest commercially available established deoxyfluorination agents.^11^ As outlined in Table 1, all reactions were carried out at room temperature using dry CH_2_Cl_2_ as the solvent. Other solvents (*e.g*. THF or toluene) were tested too, but did not allow for any reasonable conversion and non-reproducible results were obtained thereby. First experiments where DAST (**1**) was added to a solution of **2a** showed that conversion is mainly depending on the amount of **1** (compare entries 1–3) as longer reaction times were not beneficial in case of the slowly converting experiments. These results suggest that DAST partially decomposes under the conditions. Noteworthy however, all the reactions turned out to be very clean and “spot-to-spot”, with no other fluorinated products being formed. In each case the reaction proceeded with high levels of stereospecificity, giving the configurationally inverted (*R*)-**3a**. Absolute configuration of starting material (*S*)-**2a** and product (*R*)-**3a** were assigned by comparison of optical rotation and HPLC retention time orders with previous reports.^7,8,12,13^ The stereochemical course (inversion or retention of configuration) of DAST-mediated deoxyfluorinations has been a matter of discussion^4,5^ and mostly depends on the nature of the substrate. For our target transformation the observed inversion suggests a clean S_N_2-type mechanism^14^ and as can be seen from all the results summarized in Table 1, the observed levels of enantiospecificity were always satisfying (see Scheme 2 for the proposed mechanistic scenario). In order to improve the conversion, we next changed the order of addition (entries 4–6). Adding substrate **2a** to DAST (add. order B) had a beneficial effect, allowing for high conversion by using just two equivalents of reagent **1** (NMR yields determined by addition of an internal standard were in the same range as the conversion of **2a** and the isolated yields after column chromatography were higher than 70% in all these cases).

Interestingly, when carrying out the reaction with other deoxyfluorination methods, like the above mentioned CuF_2_ protocol^6*g*^ or by using PyFluor,^6*c*^ absolutely no formation of product **3a** could be achieved under otherwise identical conditions. Unfortunately, the reactions with two equivalents of **1** often stalled at around 90% conversion (and more restricted conversions were later observed during the investigation of the application scope as well). Thus, we finally used a slightly larger excess of **1** (entry 6, add. order C), which allowed for robust and reproducible reaction conditions with very high levels of enantiospecifity (repeating the reaction several times always resulted in >98.0% es).

With reliable and highly stereospecific conditions at hand, we next investigated the application scope of this methodology (Scheme 2). A variety of differently substituted indanone-based α-hydroxy-β-ketoesters **2** were well tolerated, giving the corresponding configurationally inverted products **3** in satisfying yields and with high levels of enantiospecificity in most cases. Interestingly, the fluorine containing **3d** and **3h** were obtained with slightly lower es values, but still in an acceptable range. In sharp contrast to the other substrates, the 5-methoxy-substituted **2i** performed very slow only, and even adding additional amounts of DAST did not allow for a higher conversion and yield of product **3i**. Most likely the strong electron donating effect of the methoxy group *para* to the carbonyl group leads to an increased contribution of the enolate resonance structure, which as a result leads to a lower nucleophilicity of the α-OH group and thus slows down the deoxyfluorination process. Unfortunately, when using tetralone-based starting material **2o**, even large excesses of DAST did not allow for any product formation and resulted in more or less quantitative recovery of alcohol **2o**. A possible explanation maybe that the nucleophilic fluoride attack to the *in situ* activated alcohol has to proceed *via* a pseudo-axial trajectory on the 6-ring system, where 1,2- and 1,3-(pseudo)-diaxial interactions are much stronger than on the indanone-based 5-ring systems.

In conclusion, we have developed an operationally simple method for the stereospecific deoxyfluorination of enantio-enriched α-hydroxy-β-ketoesters **2**, by reaction with DAST (**1**). This protocol works well for a variety of indanone-based ketoesters **2**, without any occurrence of carbonyl-group deoxofluorination or any other side reactions.

## General reaction procedure

A solution of α-hydroxy-β-ketoester **2** (0.1 mmol) in dry CH_2_Cl_2_ (2.5 mL) is added dropwise over 15 min to a stirred solution of DAST (**1**; 200 μL, 1 M in CH_2_Cl_2_) in 2.5 mL dry CH_2_Cl_2_ at room temperature (Ar-atmosphere). After stirring for 20 h, another portion of DAST (**1**; 200 μL, 1 M in CH_2_Cl_2_) is added and the mixture is stirred for additional 20 h (40 h total reaction time). The reaction is quenched with 5 mL saturated aq. NaHCO_3_, and the aqueous phase is extracted with dichloromethane (3 × 5 mL). The combined organic phases are dried over anhydrous Na_2_SO_4_, filtered and the solvent is removed *in vacuo*. The residue is purified by silica gel column chromatography (heptanes/EtOAc) to afford the targeted fluorinated products **3** in the reported yields and enantiospecificities.

## Supplementary Material

†Electronic supplementary information (ESI) available: Full experimental details and analytical data. See DOI: 10.1039/d0ob02152k

SI

## Figures and Tables

**Scheme 1:**
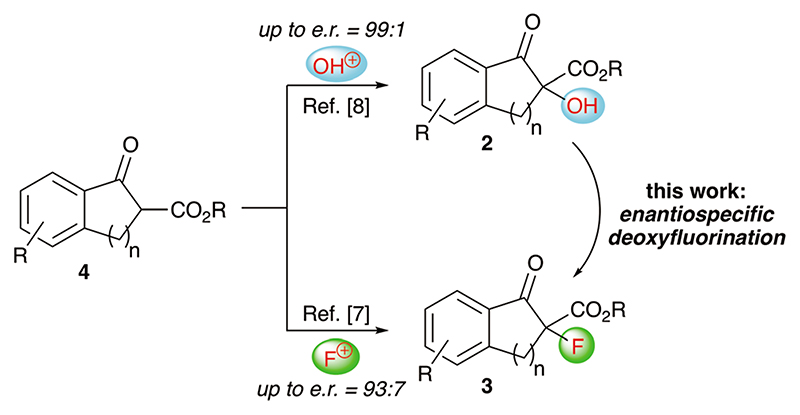
Our previously developed α-hydroxylation and α-fluorination of β-ketoesters **4** and the herein investigated deoxyfluorination of alcohols **2** to access α-F-β-ketoesters **3**.

**Scheme 2:**
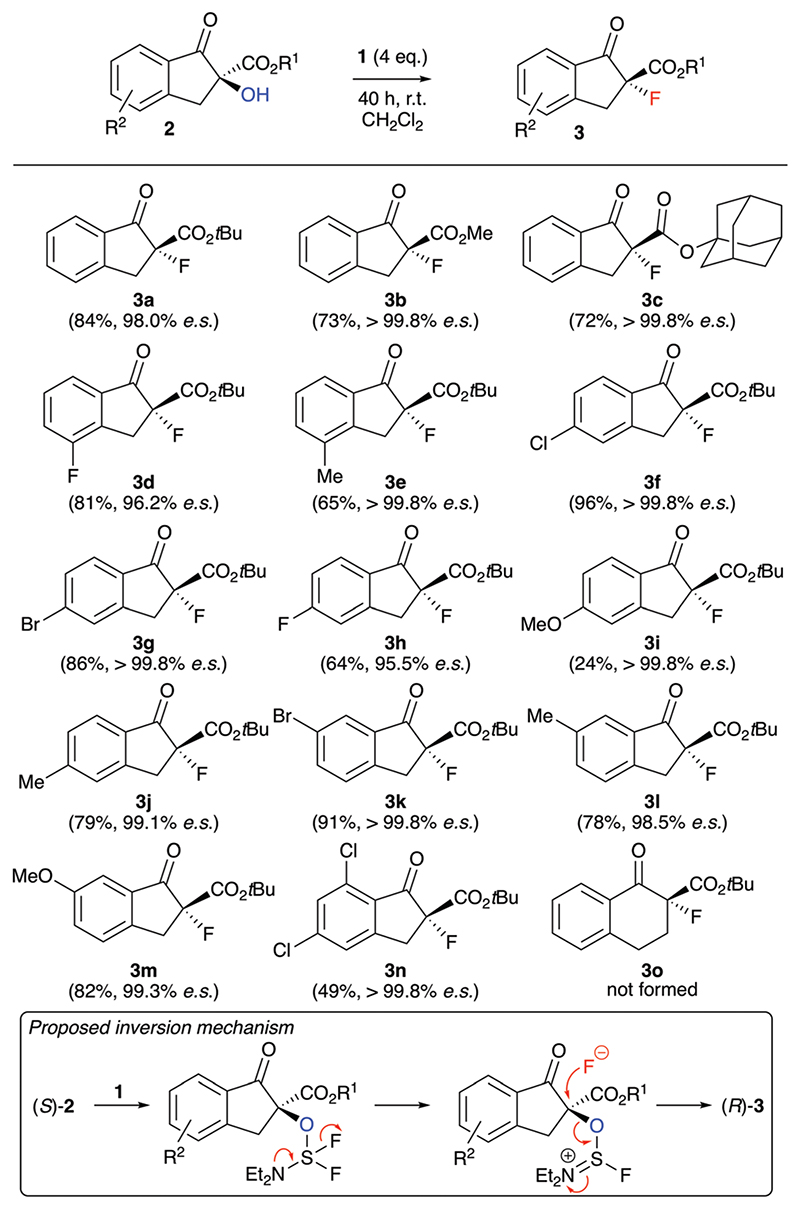
Application scope employing the conditions shown in Table 1, entry 6 (all reactions were run using 0.05–0.1 mmol **2**) and the proposed stereospecific inversion mechanism.

**Table 1:** Identification of the best-suited conditions for the deoxyfluorination of 2a ^*a*^

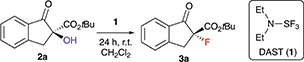						
Entry	1 (eq.)	Addition order^*b*^	Conv.^*c*^	ee (2a)^*d*^	ee (3a)^*d*^	es^*e*^[%]
1	1.1	A	0	—	—	—
2	4	A	85	90.2	88.9	98.5
3	8	A	95	90.2	89.5	99.2
4	1.1	B	84	88.5	88.1	99.6
5	2	B	91	90.2	89.0	98.6
6	4	C	>95	95.3	93.1	98.0

a All reactions were run using 0.05 mmol **2a** in a total volume of 2.5 mL CH_2_Cl_2_ under Ar.

b Addition order A: Dropwise addition of **1** in CH_2_Cl_2_ (1.25 mL) to **2a** in CH_2_Cl_2_ (1.25 mL) over 15 min; B: Dropwise addition of **2a** in CH_2_Cl_2_ (1.25 mL) to **1** in CH_2_Cl_2_ (1.25 mL) over 15 min; C: Dropwise addition of **2a** in CH_2_Cl_2_ (1.25 mL) to 2 eq. **1** in CH_2_Cl_2_ (1.25 mL) over 15 min followed by stirring for 20 h and addition of another 2 eq. of **1** and stirring for further 20 h (40 h total reaction time).

c Determined by ^1^H NMR of the crude reaction mixture.

d Determined by HPLC using a chiral stationary phase.

e 100 × ee (**3a**)/ee (**2a**); absolute configuration was assigned as described previously.^7,8,12,13^
