# Supplementary material for: Enantiospecific deoxyfluorination of cyclic α-OH-β-ketoesters†
Source: Org Biomol Chem. Author manuscript; Available in PMC 2021 Feb 2. (PMC7116657; doi:10.1039/d0ob02152k)

Electronic Supplementary Material (ESI) for Organic & Biomolecular Chemistry.
This journal is © The Royal Society of Chemistry 2020

# SUPPORTING INFORMATION

## Enantiospecific Deoxyfluorination of Cyclic α-OH-β-Ketoesters

**Christopher Mairhofer,[a] Victoria Haider,[a] Thomas Bögl[b] and Mario Waser[a*]**

*a) Institute of Organic Chemistry, Johannes Kepler University Linz, Altenbergerstraße 69,*
*4040 Linz, Austria.*
*Fax: +43 732 2468 5411; Tel: +43 732 2468 5402;*
*E-mail: mario.waser@jku.at*

*b) Institute of Analytical Chemistry, Johannes Kepler University Linz, Altenbergerstraße 69,*
*4040 Linz, Austria.*

1. General Information: ..... 1
2. Characterization Data of *α*-Deoxyfluorinated Products ..... 3
3. Synthesis of Racemic Fluorinated Products: ..... 9
4. Copies of NMR Spectra of Deoxyfluorination Products: ..... 11
5. HPLC Traces of Hydroxylation/Deoxyfluorination Products: ..... 25
6. HRMS Data Report ..... 32

# 1. General Information:

***Spectroscopy:*** NMR spectra were recorded on a Bruker Avance III 300 MHz spectrometer with a broad band observe probe and a sample changer for 16 samples, on a Bruker Avance DRX 500 MHz spectrometer, and on a Bruker Avance III 700 MHz spectrometer with an Ascend magnet and TCI cryoprobe which are property to the Austro Czech NMR Research Center "RERI uasb". Chemical shifts (δ) are given in parts per million (ppm), coupling constants (*J*) are given in Hertz (Hz). All NMR spectra were referenced on the solvent residual peak ($CDCl_3$: δ 7.26 ppm for $^1$H NMR and δ 77.16 ppm for $^{13}$C NMR). $^1$H NMR spectra are reported as follows: chemical shift (δ ppm) (multiplicity, coupling constants, number of protons, assignment). Peak multiplicities are denoted as: s = singlet, d = doublet, t = triplet, q = quartet, m = multiplet, br = broad, dd = doublet of doublet, etc. ***Mass spectrometry:*** High resolution mass spectra (HRMS) were recorded on a Thermo Fisher Scientific LTQ Orbitrap XL hybrid FT mass spectrometer with an ESI source and an Agilent G1607A coaxial sprayer. ***Polarimetry**:* Optical rotations ($[\alpha]_\lambda^{temp}$) were measured on a Schmidt+Haensch Unipol L 100 polarimeter and data is reported as follows: [α]-values are listed in $deg \cdot cm^3 \cdot g^{-1} \cdot dm^{-1}$, concentration (*c* in g/100 mL), and solvent. ***Melting Points:*** Melting points (MP) are reported in degrees Celsius (°C), using a Büchi M-560 apparatus and are reported uncorrected. ***Chromatography:*** Preparative column chromatography was carried out using Davisil LC 60A 70– 200 MICRON silica gel. Thin layer chromatography was performed on Macherey-Nagel pre-coated TLC plates (silica gel, 60 $F_{254}$, 0.20 mm, ALUGRAM® Xtra SIL). TLC plates were visualized under 254 nm UV lamp and using permanent staining methods (*p*-anisaldehyde: 2.5 mL *p*-anisaldehyde, 93 mL absolute EtOH, 3.5 mL conc. $H_2SO_4$ and 1 mL glacial AcOH). The enantiomeric excesses (ee) were determined by HPLC analysis using a Dionex Summit HPLC system with CHIRALCEL OD-H (4.6 × 250 mm, 5 µm), OJ-H (4.6 × 250 mm, 5 µm), CHIRALPAK AD-H (4.6 × 250 mm, 5 µm) and a YMC Chiral ART Amylose SA (4.6 × 250 mm, 5 µm) chiral stationary phase. The enantiospecificity (e.s.) of the reaction is calculated as follows: % e.s. = 100 × [% ee of product]/[% ee of starting material].[1] Determination of the absolute configuration of α-fluorinated product **3a** has been reported by *Sodeoka* et al.[2] and assignment of the herein prepared **3a** was carried out by comparison of our analytical data with those literature values. All other derivatives were assigned in analogy. The starting α-hydroxylated compounds **2** were prepared in enantioenriched forms as reported recently (*vide infra*) and their absolute configuration was assigned in accordance with previous publications.[3] ***Naming of compounds:*** Compound names are those generated by ChemBioDraw® 18.2 software (PerkinElmer), following IUPAC nomenclature. ***Solvents and reagents:*** Anhydrous dichloromethane was provided by the Institute of Catalysis (JKU Linz, Austria) and was dried using a purification column composed of

(1) (a) S. E. Denmark, T. Vogler, *Chem. Eur. J.* **2009**, *15*, 11737. (b) S. E. Denmark, M. T, Burk, A. J. Hoover, *J. Am. Chem. Soc.* **2010**, *132*, 1232.
(2) Y. Hamashima, K. Yagi, H. Takano, L. Tamas; M. Sodeoka, *J. Am. Chem. Soc.* **2002**, *124*, 14530.
(3) (a) F. A. Davis, H. Liu, B.-C. Chen, P. Zhou, *Tetrahedron* **1998**, *54*, 10481. (b) S. F. McCann,G. D. Annis, R. Shapiro, D. W. Piotrowski, D. G. P. Lahm, J. K. Long, K. C. Lee, M. M. Hughes, B. J. Myers, S. M. Griswold, B. M. Reeves, R. W. March, P. L. Sharpe, P. Lowder, W. E. Barnette, K. D. Wing, K, *Pest. Manag. Sci.* **2001**, *57*, 153. (c) M. R. Acocella, O. G. Mancheño, M. Bella, K. A. Jørgensen, *J. Org. Chem.* **2004**, *69*, 8165. (d) T. Ishimaru, N. Shibata, J. Nagai, S. Nakamura, T. Toru, S. Kanemasa, *J. Am. Chem. Soc.* **2006**, *128*, 16488.

activated alumina and was stored over activated 3 Å molecular sieves. All chemicals were purchased from commercial suppliers and used without further purification unless otherwise stated. All reactions were carried out under an argon atmosphere using flame-dried glassware. β-Ketoesters were prepared according to literature-known methods.[4] Starting enantioenriched α-hydroxy-β-ketoesters **2** were prepared as reported previously.[5]

**CAUTION:** Reactions in the presence of *N*,*N*-diethylaminosulfur trifluoride (DAST) should not be conducted at temperatures >50 °C, due to safety issues. DAST is known to be thermally unstable, prone to detonation when heated >90 °C and undergoes catastrophic decomposition at ≈140 °C.[6] Moreover, explosive decomposition of DAST upon contact with water, generating tissue damaging hydrofluoric acid, has also been reported. [7]

(4) (a) T. A. Moss, D. R. Fenwick, D. J. Dixon, *J. Am. Chem. Soc.* **2008**, *130*, 10076. (b) D. Y. Kim, E. J. Park, *Org. Lett.* **2002**, *4*, 545. (c) X. Wang, Q. Lan, S. Shirakawa, K. Maruoka, *Chem. Commun.* **2010**, *46*, 321. (d) E.-M. Tanzer, W. B. Schweizer, M.-O. Ebert, R. Gilmour, *Chem. Eur. J.* **2012**, *18*, 2006. (e) M. Lian, J. Du, Q. Meng, Z. Gao, *Eur. J. Org. Chem*. **2010**, *34*, 6525.
(5) (a) J. Novacek, J. A. Izzo, M. J. Vetticatt and M. Waser, *Chem. Eur. J.* **2016**, *22*, 17339. (b) C. Mairhofer, J. Novacek and M. Waser, *Org. Lett.* **2020**, *22*, 6138.
(6) (a) W. J. Middleton, Explosive hazards with DAST *Chem. Eng. News* **1979**, *57*, 21, 43. (b) P. A. Messina, K. C. Mange, W. J. Middleton, *J. Fluorine Chem.* **1989**, *42*, 137. (c) G. S. Lal, G. P. Pez, R. J. Pesaresi, F. M. Prozonic, H. J. Cheng, *Org. Chem.* **1999**, *64*, 7048. (d) G. S. Lal, G. P. Pez, R. J. Pesaresi, F. M. Prozonic, *Chem. Commun.* **1999**, *2*, 215.
(7) J. Cochran, Laboratory explosions *Chem. Eng. News* **1979**, *57*, 12, 4.

## 2. Characterization Data of *α*-Deoxyfluorinated Products

**_tert_-Butyl (*R*)-2-fluoro-1-oxo-2,3-dihydro-1H-indene-2-carboxylate (3a):**

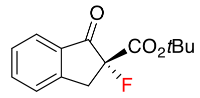

**3a**

*Enantiospecificity:* 98.0% e.s. Prepared following the general procedure on a 0.1 mmol (24.8 mg of (*S*)-**2a**, 94.6% ee) scale. Purification by silica gel column chromatography using heptanes/EtOAc (15:1 to 8:1) provided the title compound (*R*)-**3a** as a white crystalline solid (21.0 mg, 84% yield, 92.8% ee). **MP**: 41.6−43.0 °C (EtOAc/heptane). **TLC** (30% EtOAc/heptane): $R_f$ = 0.43 (UV, *p*-anisaldehyde). Analytical data are in accordance with those reported in the literature.[8] $[\alpha]_D^{23.3}$ = +3.8 (*c* 0.81, $CHCl_3$, 91.0% ee). **$^1$H NMR** (500 MHz, $CDCl_3$, 298 K) δ 7.82 (d, *J* = 7.7 Hz, 1H, Ar***H***), 7.68 (t, *J* = 7.5 Hz, 1H, Ar***H***), 7.49 (d, *J* = 7.7 Hz, 1H, Ar***H***), 7.45 (t, *J* = 7.5 Hz, 1H, Ar***H***), 3.72 (dd, *J* = 17.4, 10.8 Hz, 1H, C***H***H), 3.39 (dd, *J* = 22.9, 17.5 Hz, 1H, CH***H***), 1.42 (s, 9H, C$\boldsymbol{H}_3$). **$^{13}$C NMR** (126 MHz, $CDCl_3$, 298 K) δ 195.9 (d, *J* = 18.2 Hz, 1C, ***C***=O), 166.4 (d, *J* = 27.8 Hz, 1C, ***C***$O_2$R), 151.1 (d, *J* = 3.8 Hz, 1C, $\boldsymbol{C}_{Ar}$), 136.6 (1C, $\boldsymbol{C}_{Ar}$), 133.7 (d, *J* = 1.3 Hz, 1C, $\boldsymbol{C}_{Ar}$), 128.6 (1C, $\boldsymbol{C}_{Ar}$), 126.6 (d, *J* = 1.5 Hz, 1C, $\boldsymbol{C}_{Ar}$), 125.6 (d, *J* = 1.2 Hz, 1C, $\boldsymbol{C}_{Ar}$), 94.5 (d, *J* = 201.7 Hz, 1C, ***C***qF), 84.3 (1C, ***C***q$Me_3$), 38.5 (d, *J* = 24.2 Hz, 1C, ***C***$H_2$), 27.9 (3C, ***C***$H_3$). **$^{19}$F NMR** (471 MHz, $CDCl_3$, 298 K) δ −164.0 (dd, *J* = 22.8, 10.7 Hz). **HRMS** (ESI-Orbitrap, MeOH) *m*/*z*: $[M + NH_4]^+$ calcd for $C_{14}H_{19}FO_3N$, 268.1343; found, 268.1334. **HPLC**: Chiralpak AD-H (*n*-hexane:*i*-PrOH = 200:1, flow rate 0.75 mL/min, 10 °C, λ = 240 nm), retention times $t_R$(minor) = 25.6 min, $t_R$(major) = 31.2 min.

**Methyl (*R*)-2-fluoro-1-oxo-2,3-dihydro-1H-indene-2-carboxylate (3b):**

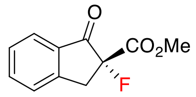

**3b**

*Enantiospecificity:* >99.8% e.s. Prepared following the general procedure on a 70 µmol (14.4 mg of (*S*)-**2b**, 68.0% ee) scale. Purification by silica gel column chromatography using heptanes/EtOAc (12:1 to 6:1) provided the title compound (*R*)-**3b** as a white crystalline solid (10.6 mg, 51 µmol, 73% yield, 68.3% ee). **MP**: 99.2−101.6 °C (EtOAc/heptane). **TLC** (30% EtOAc/heptane): $R_f$ = 0.29 (UV, *p*-anisaldehyde). Analytical data are in accordance with those reported in the literature.[8] $[\alpha]_D^{22.9}$ = −18.3 (*c* 0.50, $CH_2Cl_2$). **$^1$H NMR** (500 MHz, $CDCl_3$, 298 K) δ 7.85 (d, *J* = 7.7 Hz, 1H, Ar***H***), 7.71 (t, *J* = 7.5 Hz, 1H, Ar***H***), 7.53 – 7.44 (m, 2H, Ar***H***), 3.82 (s, 3H, C$\boldsymbol{H}_3$), 3.80 (dd, *J* = 17.6, 11.4 Hz, 1H, C***H***H), 3.45 (dd, *J* = 23.3, 17.6 Hz, 1H, CH***H***). **$^{13}$C NMR** (126 MHz, $CDCl_3$, 298 K) δ 195.3 (d, *J* = 18.1 Hz, 1C, ***C***=O), 167.9 (d, *J* = 27.8 Hz, 1C, ***C***$O_2$R), 151.0 (d, *J* = 3.5 Hz, 1C, $\boldsymbol{C}_{Ar}$), 136.9 (1C, $\boldsymbol{C}_{Ar}$), 133.4 (1C, $\boldsymbol{C}_{Ar}$), 128.8 (1C, $\boldsymbol{C}_{Ar}$), 126.7 (d, *J* = 1.5 Hz, (1C, $\boldsymbol{C}_{Ar}$), 125.9 (1C, $\boldsymbol{C}_{Ar}$), 94.8 (d, *J* = 201.8 Hz, 1C, ***C***qF), 53.4 (1C, ***C***$H_2$), 38.4 (d, *J* = 23.7 Hz, 1C, ***C***$H_3$). **$^{19}$F NMR** (471 MHz, $CDCl_3$, 298 K) δ −164.5 (dd, *J* = 23.3, 11.2 Hz). **HRMS** (ESI-Orbitrap, MeOH) *m*/*z*: $[M + H]^+$ calcd for $C_{11}H_{10}FO_3$, 209.0608; found, 209.0604. **HPLC**: Chiralcel OD-H (*n*-hexane:*i*-PrOH = 95:5, flow rate 0.75 mL/min, 10 °C, λ = 270 nm), retention times $t_R$(major) = 22.6 min, $t_R$(minor) = 28.1 min.

(8) J. Novacek, M. Waser, *Eur. J. Org. Chem.* **2014**, 802.

**Adamantan-1-yl (*R*)-2-fluoro-1-oxo-2,3-dihydro-1H-indene-2-carboxylate (3c):**

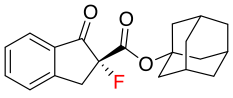


**3c**

*Enantiospecificity:* >99.8% e.s. Prepared following the general procedure on a 80 µmol (26.1 mg of (*S*)-**2c**, 80.4% ee) scale. Purification by silica gel column chromatography using heptanes/EtOAc (20:1 to 10:1) provided the title compound (*R*)-**3c** as a white crystalline solid (18.9 mg, 58 µmol, 72% yield, 80.6% ee). **MP**: 80.2−81.3 °C (EtOAc/heptane). **TLC** (30% EtOAc/heptane): $R_f = 0.51$ (UV, *p*-anisaldehyde). Analytical data are in accordance with those reported in the literature.[8] $[\alpha]_D^{23.0} = -1.4$ (*c* 0.82, $CH_2Cl_2$). **$^1$H NMR** (500 MHz, $CDCl_3$, 298 K) δ 7.80 (d, *J* = 7.7 Hz, 1H, Ar***H***), 7.67 (t, *J* = 7.5 Hz, 1H, Ar***H***), 7.48 (d, *J* = 7.8 Hz, 1H, Ar***H***), 7.43 (t, *J* = 7.5 Hz, 1H, Ar***H***), 3.72 (dd, *J* = 17.4, 10.5 Hz, 1H, C***H***H), 3.38 (dd, *J* = 22.8, 17.5 Hz, 1H, CH***H***), 2.12 (s, 3H, C***H***), 2.03 (s, 6H, C$\boldsymbol{H}_2$), 1.60 (s, 6H, C$\boldsymbol{H}_2$). **$^{13}$C NMR** (126 MHz, $CDCl_3$, 298 K) δ 196.0 (d, *J* = 18.5 Hz, 1C, ***C***=O), 165.9 (d, *J* = 27.8 Hz, 1C, ***C***$O_2$R), 151.1 (d, *J* = 4.0 Hz, 1C, $\boldsymbol{C}_{Ar}$), 136.5 (1C, $\boldsymbol{C}_{Ar}$), 133.7 (d, *J* = 1.3 Hz, 1C, $\boldsymbol{C}_{Ar}$), 128.5 (1C, $\boldsymbol{C}_{Ar}$), 126.5 (d, *J* = 1.4 Hz, 1C, $\boldsymbol{C}_{Ar}$), 125.5 (d, *J* = 1.3 Hz, 1C, $\boldsymbol{C}_{Ar}$), 94.4 (d, *J* = 201.5 Hz, 1C, ***C***qF), 84.2 (1C, ***C***qAd), 41.1 (3C, $\boldsymbol{C}_{Ad}H_2$), 38.5 (d, *J* = 24.0 Hz, 1C, ***C***$H_2$), 36.0 (3C, $\boldsymbol{C}_{Ad}$H), 30.9 (3C, $\boldsymbol{C}_{Ad}H_2$). **$^{19}$F NMR** (471 MHz, $CDCl_3$, 298 K) δ −164.1 (dd, *J* = 22.8, 10.4 Hz). **HRMS** (ESI-Orbitrap, MeOH) *m*/*z*: $[M + NH_4]^+$ calcd for $C_{20}H_{25}FNO_3N$, 346.1813; found, 346.1801. **HPLC**: Chiralpak AD-H (*n*-hexane:*i*-PrOH = 99:1, flow rate 0.7 mL/min, 10 °C, λ = 270 nm), retention times $t_R$(minor) = 32.7 min, $t_R$(major) = 46.0 min.

***tert*-Butyl (*R*)-2,4-difluoro-1-oxo-2,3-dihydro-1H-indene-2-carboxylate (3d):**

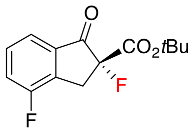


**3d**

*Enantiospecificity:* 96.2% e.s. Prepared following the general procedure on a 65 µmol (17.3 mg of (*S*)-**2d**, 85.0% ee) scale. Purification by silica gel column chromatography using heptanes/EtOAc (20:1 to 9:1) provided the title compound (*R*)-**3d** as a white crystalline solid (14.1 mg, 52 µmol, 81% yield, 81.8% ee). **MP**: 91.2–91.9 °C (EtOAc/heptane). **TLC** (30% EtOAc/heptane): $R_f = 0.53$ (UV, *p*-anisaldehyde). $[\alpha]_D^{23.5} = +2.5$ (*c* 0.69, $CH_2Cl_2$). **$^1$H NMR** (500 MHz, $CDCl_3$, 298 K) δ 7.64 (d, *J* = 7.6 Hz, 1H, Ar***H***), 7.51 – 7.43 (m, 1H, Ar***H***), 7.38 (t, *J* = 8.3 Hz, 1H, Ar***H***), 3.75 (dd, *J* = 17.8, 10.9 Hz, 1H, C***H***H), 3.37 (dd, *J* = 22.6, 17.8 Hz, 1H, CH***H***), 1.44 (s, 9H, C$\boldsymbol{H}_3$). **$^{13}$C NMR** (126 MHz, $CDCl_3$, 298 K) δ 194.8 (dd, *J* = 18.8, 3.0 Hz, 1C, ***C***=O), 165.9 (d, *J* = 27.4 Hz, 1C, ***C***$O_2$R), 159.7 (dd, *J* = 251.6, 1.2 Hz, 1C, $\boldsymbol{C}_{Ar}$), 137.0 (dd, *J* = 19.5, 4.0 Hz, 1C, $\boldsymbol{C}_{Ar}$), 136.2 (dd, *J* = 4.8, 1.4 Hz, 1C, $\boldsymbol{C}_{Ar}$), 130.6 (d, *J* = 6.3 Hz, 1C, $\boldsymbol{C}_{Ar}$), 122.7 (d, *J* = 19.8 Hz, 1C, $\boldsymbol{C}_{Ar}$), 121.3 (dd, *J* = 4.2, 1.2 Hz, 1C, $\boldsymbol{C}_{Ar}$), 94.0 (d, *J* = 203.0 Hz, 1C, ***C***qF), 84.7 (1C, ***C***q$Me_3$), 34.4 (d, *J* = 25.3 Hz, 1C, ***C***$H_2$), 27.9 (3C, ***C***$H_3$). **$^{19}$F NMR** (471 MHz, $CDCl_3$, 298 K) δ −118.1 (m), −163.3 (dd, *J* = 22.6, 10.6 Hz). **HRMS** (ESI-Orbitrap, MeOH) *m*/*z*: $[M + NH_4]^+$ calcd for $C_{14}H_{18}F_2O_3N$, 286.1249; found, 286.1238. **HPLC**: Chiralpak AD-H (*n*-hexane:*i*-PrOH = 95:5, flow rate 0.75 mL/min, 10 °C, λ = 240 nm), retention times $t_R$(minor) = 8.0 min, $t_R$(major) = 11.7 min.

**tert-Butyl (R)-2-fluoro-4-methyl-1-oxo-2,3-dihydro-1H-indene-2-carboxylate (3e):**

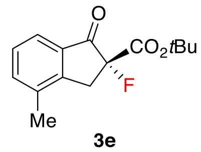


**3e**

*Enantiospecificity:* >99.8% e.s. Prepared following the general procedure on a 80 µmol (21.0 mg of (*S*)-**2e**, 78.1% ee) scale. Purification by silica gel column chromatography using heptanes/EtOAc (20:1 to 10:1) provided the title compound (*R*)-**3e** as a yellow crystalline solid (13,7 mg, 52 µmol, 65% yield, 78.4% ee). **MP**: 63.7−64.6 °C (EtOAc/heptane). **TLC** (30% EtOAc/heptane): $R_f$ = 0.45 (UV, *p*-anisaldehyde). $[\alpha]_D^{23.1}$ = −23.8 (*c* 0.68, $CH_2Cl_2$). **$^1$H NMR** (500 MHz, $CDCl_3$, 298 K) δ 7.66 (d, *J* = 7.6 Hz, 1H, Ar***H***), 7.49 (d, *J* = 7.3 Hz, 1H, Ar***H***), 7.36 (t, *J* = 7.5 Hz, 1H, Ar***H***), 3.61 (dd, *J* = 17.5, 11.3 Hz, 1H, C***H***H), 3.26 (dd, *J* = 23.2, 17.5 Hz, 1H, CH***H***), 2.36 (s, 3H, ArC$\boldsymbol{H}_3$), 1.44 (s, 9H, C$\boldsymbol{H}_3$). **$^{13}$C NMR** (126 MHz, $CDCl_3$, 298 K) δ 196.2 (d, *J* = 18.2 Hz, 1C, ***C***=O), 166.6 (d, *J* = 27.4 Hz, 1C, ***C***$O_2$R), 150.2 (d, *J* = 3.4 Hz, 1C, $\boldsymbol{C}_{Ar}$), 137.1 (1C, $\boldsymbol{C}_{Ar}$), 135.9 (d, *J* = 1.3 Hz, 1C, $\boldsymbol{C}_{Ar}$), 133.5 (d, *J* = 1.2 Hz, 1C, $\boldsymbol{C}_{Ar}$), 128.8 (1C, $\boldsymbol{C}_{Ar}$), 123.0 (d, *J* = 1.2 Hz, 1C, $\boldsymbol{C}_{Ar}$), 94.5 (d, *J* = 201.3 Hz, 1C, ***C***qF), 84.3 (1C, ***C***q$Me_3$), 37.4 (d, *J* = 24.1 Hz, 1C, ***C***$H_2$), 28.0 (3C, ***C***$H_3$), 17.9 (1C, Ar***C***$H_3$). **$^{19}$F NMR** (471 MHz, $CDCl_3$, 298 K) δ −163.3 (dd, *J* = 23.2, 11.4 Hz). **HRMS** (ESI-Orbitrap, MeOH) *m/z*: $[M + NH_4]^+$ calcd for $C_{15}H_{21}FO_3N$, 282.1500; found, 282.1490. **HPLC**: Chiralpak AD-H (*n*-hexane:*i*-PrOH = 99:1, flow rate 1.0 mL/min, 10 °C, λ = 240 nm), retention times $t_R$(minor) = 10.7 min, $t_R$(major) = 13.4 min.

**tert-Butyl (R)-5-chloro-2-fluoro-1-oxo-2,3-dihydro-1H-indene-2-carboxylate (3f):**

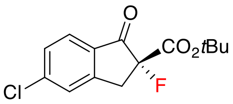


**3f**

*Enantiospecificity:* >99.8% e.s. Prepared following the general procedure on a 80 µmol (22.6 mg of (*S*)-**2f**, 84.6% ee) scale. Purification by silica gel column chromatography using heptanes/EtOAc (20:1 to 10:1) provided the title compound (*R*)-**3f** as a white crystalline solid (21.9 mg, 77 µmol, 96% yield, 84.9% ee). **MP**: 100.8−101.9 °C (EtOAc/heptane). **TLC** (30% EtOAc/heptane): $R_f$ = 0.56 (UV, *p*-anisaldehyde). Analytical data are in accordance with those reported in the literature.[9] $[\alpha]_D^{23.3}$ = −31.4 (*c* 1.00, $CH_2Cl_2$). **$^1$H NMR** (500 MHz, $CDCl_3$, 298 K) δ 7.75 (d, *J* = 8.2 Hz, 1H, Ar***H***), 7.49 (s, 1H, Ar***H***), 7.43 (dd, *J* = 8.1, 1.9 Hz, 1H, Ar***H***), 3.70 (dd, *J* = 17.7, 10.6 Hz, 1H, C***H***H), 3.37 (dd, *J* = 22.6, 17.7 Hz, 1H, CH***H***), 1.43 (s, 9H, C$\boldsymbol{H}_3$). **$^{13}$C NMR** (126 MHz, $CDCl_3$, 298 K) δ 194.5 (d, *J* = 18.5 Hz, 1C, ***C***=O), 166.0 (d, *J* = 27.7 Hz, 1C, ***C***$O_2$R), 152.4 (d, *J* = 3.9 Hz, 1C, $\boldsymbol{C}_{Ar}$), 143.3 (1C, $\boldsymbol{C}_{Ar}$), 132.1 (d, *J* = 1.6 Hz, 1C, $\boldsymbol{C}_{Ar}$), 129.5 (1C, $\boldsymbol{C}_{Ar}$), 126.9 (d, *J* = 1.6 Hz, 1C, $\boldsymbol{C}_{Ar}$), 126.6 (d, *J* = 1.2 Hz, 1C, $\boldsymbol{C}_{Ar}$), 94.3 (d, *J* = 202.8 Hz, 1C, ***C***qF), 84.6 (1C, ***C***q$Me_3$), 38.1 (d, *J* = 24.5 Hz, 1C, ***C***$H_2$), 27.9 (3C, ***C***$H_3$). **$^{19}$F NMR** (471 MHz, $CDCl_3$, 298 K) δ −163.32 (dd, *J* = 22.6, 10.5 Hz). (ESI-Orbitrap, MeOH) *m/z*: $[M + NH_4]^+$ calcd for $C_{14}H_{18}ClFO_3N$, 302.0954; found, 302.0942. **HPLC**: Chiralpak AD-H (*n*-hexane:*i*-PrOH = 99:1, flow rate 1.0 mL/min, 10 °C, λ = 220 nm), retention times $t_R$(minor) = 13.8 min, $t_R$(major) = 18.1 min.

(9) X. Gu, Y. Zhang, Z.-J. Xu, C.-M. Che, *Chem. Commun.* **2014**, *50*, 7870.

**tert-Butyl (R)-5-bromo-2-fluoro-1-oxo-2,3-dihydro-1H-indene-2-carboxylate (3g):**

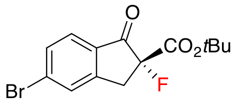


**3g**

*Enantiospecificity:* >99.8% e.s. Prepared following the general procedure on a 70 µmol (22.9 mg of (*S*)-**2g**, 90.6% ee) scale. Purification by silica gel column chromatography using heptanes/EtOAc (20:1 to 9:1) provided the title compound (*R*)-**3g** as a pale yellow crystalline solid (19.8 mg, 60 µmol, 86% yield, 92.4% ee). **MP**: 121.9–122.5 °C (EtOAc/heptane). **TLC** (30% EtOAc/heptane): $R_f$ = 0.51 (UV, *p*-anisaldehyde). Analytical data are in accordance with those reported in the literature.[8] $[\alpha]_D^{23.2}$ = −25.3 (*c* 1.00, $CH_2Cl_2$). **$^1$H NMR** (500 MHz, $CDCl_3$, 298 K) δ 7.70 – 7.66 (m, 1H, Ar***H***), 7.60 (d, *J* = 8.1 Hz, 2H, Ar***H***), 3.70 (dd, *J* = 17.7, 10.6 Hz, 1H, C***H***H), 3.38 (dd, *J* = 22.6, 17.6 Hz, 1H, CH***H***), 1.43 (s, 9H, ***C***$H_3$). **$^{13}$C NMR** (126 MHz, $CDCl_3$, 298 K) δ 194.7 (d, *J* = 18.5 Hz, 1C, ***C***=O), 166.0 (d, *J* = 27.4 Hz, 1C, ***C***$O_2$R), 152.5 (d, *J* = 4.1 Hz, 1C, ***C***$_{Ar}$), 132.5 (d, *J* = 1.3 Hz, 1C, ***C***$_{Ar}$), 132.4 (1C, ***C***$_{Ar}$), 132.2 (1C, ***C***$_{Ar}$), 130.0 (d, *J* = 1.5 Hz, 1C, ***C***$_{Ar}$), 126.6 (d, *J* = 1.2 Hz, 1C, ***C***$_{Ar}$), 94.2 (d, *J* = 202.8 Hz, 1C, ***C***qF), 84.6 (1C, ***C***q$Me_3$), 38.1 (d, *J* = 24.5 Hz, 1C, ***C***$H_2$), 28.0 (3C, ***C***$H_3$). **$^{19}$F NMR** (471 MHz, $CDCl_3$, 298 K) δ −163.4 (dd, *J* = 22.9, 10.5 Hz). **HRMS** (ESI-Orbitrap, MeOH) *m*/*z*: $[M + NH_4]^+$ calcd for $C_{14}H_{18}BrFO_3N$, 346.0449; found, 346.0436. **HPLC**: Chiralcel OD-H (*n*-hexane:*i*-PrOH = 95:5, flow rate 0.75 mL/min, 10 °C, λ = 220 nm), retention times $t_R$(major) = 11.2 min, $t_R$(minor) = 13.5 min.

**tert-Butyl (R)-2,5-difluoro-1-oxo-2,3-dihydro-1H-indene-2-carboxylate (3h):**

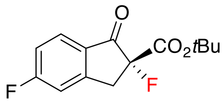


**3h**

*Enantiospecificity:* 95.5% e.s. Prepared following the general procedure on a 75 µmol (20.0 mg of (*S*)-**2h**, 85.5% ee) scale. Purification by silica gel column chromatography using heptanes/EtOAc (20:1 to 9:1) provided the title compound (*R*)-**3h** as a white crystalline solid (12.9 mg, 48 µmol, 64% yield, 81.7% ee). **MP**: 96.7−97.6 °C (EtOAc/heptane). **TLC** (30% EtOAc/heptane): $R_f$ = 0.46 (UV, *p*-anisaldehyde). Analytical data are in accordance with those reported in the literature.[8] $[\alpha]_D^{23.4}$ = −3.9 (*c* 0.57, $CH_2Cl_2$). **$^1$H NMR** (500 MHz, $CDCl_3$, 298 K) δ 7.88 – 7.80 (m, 1H, Ar***H***), 7.21 – 7.10 (m, 2H, Ar***H***), 3.71 (dd, *J* = 17.7, 10.5 Hz, 1H, C***H***H), 3.38 (dd, *J* = 22.5, 17.7 Hz, 1H, CH***H***), 1.43 (s, 9H, C***H***$_3$). **$^{13}$C NMR** (126 MHz, $CDCl_3$, 298 K) δ 194.0 (d, *J* = 18.5 Hz, 1C, ***C***=O), 168.1 (d, *J* = 259.8 Hz, 1C, ***C***$_{Ar}$F), 166.1 (d, *J* = 27.7 Hz, 1C, ***C***$O_2$R), 154.1 (dd, *J* = 10.6, 4.1 Hz, 1C, ***C***$_{Ar}$), 130.1 (t, *J* = 1.7 Hz, 1C, ***C***$_{Ar}$), 128.1 (dd, *J* = 10.7, 1.2 Hz, 1C, ***C***$_{Ar}$), 117.1 (d, *J* = 24.0 Hz, 1C, ***C***$_{Ar}$), 113.5 (dd, *J* = 23.0, 1.4 Hz, 1C, ***C***$_{Ar}$), 94.5 (d, *J* = 202.7 Hz, 1C, ***C***qF), 84.5 (1C, ***C***q$Me_3$), 38.3 (dd, *J* = 24.7, 2.1 Hz, 1C, ***C***$H_2$), 27.9 (3C, ***C***$H_3$). **$^{19}$F NMR** (471 MHz, $CDCl_3$, 298 K) δ −98.8 (m), −163.2 (dd, *J* = 22.5, 10.5 Hz). **HRMS** (ESI-Orbitrap, MeOH) *m*/*z*: $[M + NH_4]^+$ calcd for $C_{14}H_{18}F_2O_3N$, 286.1249; found, 286.1239. **HPLC**: Chiralpak AD-H (*n*-hexane:*i*-PrOH = 95:5, flow rate 0.75 mL/min, 10 °C, λ = 240 nm), retention times $t_R$(minor) = 10.9 min, $t_R$(major) = 12.6 min.

**tert-Butyl (R)-2-fluoro-5-methoxy-1-oxo-2,3-dihydro-1H-indene-2-carboxylate (3i):**

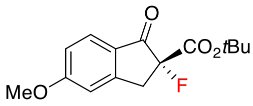


**3i**

*Enantiospecificity:* >99.8% e.s. Prepared following the general procedure on a 80 µmol (22.3 mg of (*S*)-**2i**, 88.6% ee) scale. Purification by silica gel column

chromatography using heptanes/EtOAc (12:1 to 8:1) provided the title compound (*R*)-**3i** as a white crystalline solid (5.4 mg, 19 µmol, 24% yield, 88.7% ee). **MP**: 151.9−153.4 °C (EtOAc/heptane). **TLC** (30% EtOAc/heptane): $R_f$ = 0.32 (UV, *p*-anisaldehyde). Analytical data are in accordance with those reported in the literature.[10] $[\alpha]_D^{23.4}$ = +34.1 (*c* 0.27, $CH_2Cl_2$). **$^1$H NMR** (500 MHz, $CDCl_3$, 298 K) δ 7.74 (d, *J* = 8.6 Hz, 1H, Ar***H***), 6.95 (d, *J* = 8.6 Hz, 1H, Ar***H***), 6.89 (s, 1H, Ar***H***), 3.90 (s, 3H, ArOC$\boldsymbol{H}_3$), 3.66 (dd, *J* = 17.5, 10.8 Hz, 1H, C***H***H), 3.31 (dd, *J* = 22.7, 17.5 Hz, 1H, CH***H***), 1.42 (s, 9H, C$\boldsymbol{H}_3$). **$^{13}$C NMR** (126 MHz, $CDCl_3$, 298 K) δ 193.8 (d, *J* = 18.5 Hz, 1C, ***C***=O), 166.7 (1C, $\boldsymbol{C}O_2R$), 166.5 (1C, $\boldsymbol{C}_{Ar}$), 154.3 (d, *J* = 3.8 Hz, 1C, $\boldsymbol{C}_{Ar}$), 127.4 (d, *J* = 1.2 Hz, 1C, $\boldsymbol{C}_{Ar}$), 126.7 (d, *J* = 1.3 Hz, 1C, $\boldsymbol{C}_{Ar}$), 116.6 (1C, $\boldsymbol{C}_{Ar}$), 109.8 (d, *J* = 1.5 Hz, 1C, $\boldsymbol{C}_{Ar}$), 94.9 (d, *J* = 201.1 Hz, 1C, ***C***qF), 84.0 (1C, ***C***q$Me_3$), 56.0 (1C, O***C***$H_3$), 38.4 (d, *J* = 24.4 Hz, 1C, $\boldsymbol{C}H_2$), 27.9 (3C, $\boldsymbol{C}H_3$). **$^{19}$F NMR** (471 MHz, $CDCl_3$) δ −162.9 (dd, *J* = 22.9, 11.4 Hz). **HRMS** (ESI-Orbitrap, MeOH) *m*/*z*: $[M + NH_4]^+$ calcd for $C_{15}H_{21}FO_4N$, 298.1449; found, 298.1437. **HPLC**: Chiralcel OJ-H (*n*-hexane:*i*-PrOH = 80:20, flow rate 0.9 mL/min, 10 °C, λ = 270 nm), retention times $t_R$(major) = 17.0 min, $t_R$(minor) = 18.7 min.

***tert*-Butyl (*R*)-2-fluoro-5-methyl-1-oxo-2,3-dihydro-1H-indene-2-carboxylate (3j):**

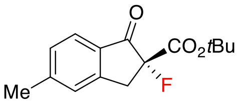


**3j**

*Enantiospecificity:* 99.1% e.s. Prepared following the general procedure on a 80 µmol (21.0 mg of (*S*)-**2j**, 89.3% ee) scale. Purification by silica gel column chromatography using heptanes/EtOAc (15:1 to 9:1) provided the title compound (*R*)-**3j** as a yellow crystalline solid (16.7 mg, 63 µmol, 79% yield, 88.5% ee). **MP**: 81.9−82.8 °C (EtOAc/heptane). **TLC** (30% EtOAc/heptane): $R_f$ = 0.41 (UV, *p*-anisaldehyde). $[\alpha]_D^{22.1}$ = −11.7 (*c* 0.92, $CH_2Cl_2$). **$^1$H NMR** (500 MHz, $CDCl_3$, 298 K) δ 7.70 (d, *J* = 7.9 Hz, 1H, Ar***H***), 7.27 (s, 1H, Ar***H***), 7.24 (d, *J* = 7.9 Hz, 1H, Ar***H***), 3.66 (dd, *J* = 17.5, 10.8 Hz, 1H, C***H***H), 3.32 (dd, *J* = 22.9, 17.5 Hz, 1H, CH***H***), 2.46 (s, 3H, ArC$\boldsymbol{H}_3$), 1.42 (s, 9H, C$\boldsymbol{H}_3$). **$^{13}$C NMR** (126 MHz, $CDCl_3$, 298 K) δ 195.3 (d, *J* = 18.2 Hz, 1C, ***C***=O), 166.5 (d, *J* = 27.5 Hz, 1C, $\boldsymbol{C}O_2R$), 151.6 (d, *J* = 3.8 Hz, 1C, $\boldsymbol{C}_{Ar}$), 148.3 (1C, $\boldsymbol{C}_{Ar}$), 131.4 (d, *J* = 1.2 Hz, 1C, $\boldsymbol{C}_{Ar}$), 129.9 (1C, $\boldsymbol{C}_{Ar}$), 126.9 (d, *J* = 1.3 Hz, 1C, $\boldsymbol{C}_{Ar}$), 125.4 (d, *J* = 1.2 Hz, 1C, $\boldsymbol{C}_{Ar}$), 94.8 (d, *J* = 201.5 Hz, 1C, ***C***qF), 84.1 (1C, ***C***q$Me_3$), 38.3 (d, *J* = 24.3 Hz, 1C, $\boldsymbol{C}H_2$), 27.9 (3C, $\boldsymbol{C}H_3$), 22.4 (1C, Ar***C***$H_3$). **$^{19}$F NMR** (471 MHz, $CDCl_3$, 298 K) δ −163.6 (dd, *J* = 22.9, 10.8 Hz). **HRMS** (ESI-Orbitrap, MeOH) *m*/*z*: $[M + NH_4]^+$ calcd for $C_{15}H_{21}FO_3N$, 282.1500; found, 282.1492. **HPLC**: Chiralcel OD-H (*n*-hexane:*i*-PrOH = 99:1, flow rate 1.0 mL/min, 10 °C, λ = 240 nm), retention times $t_R$(major) = 12.3 min, $t_R$(minor) = 13.2 min.

***tert*-Butyl (*R*)-6-bromo-2-fluoro-1-oxo-2,3-dihydro-1H-indene-2-carboxylate (3k):**

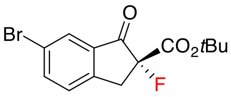


**3k**

*Enantiospecificity:* >99.8% e.s. Prepared following the general procedure on a 70 µmol (22.9 mg of (*S*)-**2k**, 84.5% ee) scale. Purification by silica gel column chromatography using heptanes/EtOAc (20:1 to 9:1) provided the title compound (*R*)-**3k** as a white crystalline solid (21.0 mg, 64 µmol, 91% yield, 85.1% ee). **MP**: 87.4−88.4 °C (EtOAc/heptane). **TLC** (30% EtOAc/heptane): $R_f$ = 0.48 (UV, *p*-anisaldehyde).

(10) X. Wang, Q. Lan, S. Shirakawa, K. Maruoka, *Chem. Commun.* **2010**, *46*, 321.

**[α]$_D$$^{23.6}$** = +12.0 (*c* 0.88, $CH_2Cl_2$, 81.3% ee). **$^1$H NMR** (500 MHz, $CDCl_3$, 298 K) δ 7.94 (s, 1H, Ar***H***), 7.78 (dd, *J* = 8.1, 2.0 Hz, 1H, Ar***H***), 7.38 (d, *J* = 8.1 Hz, 1H, Ar***H***), 3.67 (dd, *J* = 17.6, 10.4 Hz, 1H, C***H***H), 3.33 (dd, *J* = 22.5, 17.6 Hz, 1H, CH***H***), 1.43 (s, 9H, C***H***$_3$). **$^{13}$C NMR** (126 MHz, $CDCl_3$, 298 K) δ 194.6 (d, *J* = 18.6 Hz, 1C, ***C***=O), 165.9 (d, *J* = 27.6 Hz, 1C, ***C***$O_2$R), 149.6 (d, *J* = 3.8 Hz, 1C, ***C***$_{Ar}$), 139.3 (1C, ***C***$_{Ar}$), 135.4 (d, *J* = 1.3 Hz, 1C, ***C***$_{Ar}$), 128.3 (d, *J* = 1.2 Hz, 1C, ***C***$_{Ar}$), 128.1 (d, *J* = 1.4 Hz, 1C, ***C***$_{Ar}$), 122.7 (1C, ***C***$_{Ar}$), 94.5 (d, *J* = 203.2 Hz, 1C, ***C***qF), 84.6 (1C, ***C***q$Me_3$), 38.1 (d, *J* = 24.4 Hz, 1C, ***C***$H_2$), 27.9 (3C, ***C***$H_3$). **$^{19}$F NMR** (471 MHz, $CDCl_3$, 298 K) δ −163.4 (dd, *J* = 22.5, 10.5 Hz). **HRMS** (ESI-Orbitrap, MeOH) *m/z*: [M + $NH_4$]$^+$ calcd for $C_{14}H_{18}BrFO_3N$, 346.0449; found, 346.0453. **HPLC**: Chiralcel OD-H (*n*-hexane:*i*-PrOH = 95:5, flow rate 0.75 mL/min, 10 °C, λ = 220 nm), retention times $t_R$(minor) = 11.1 min, $t_R$(major) = 12.4 min.

***tert*-Butyl (*R*)-2-fluoro-6-methyl-1-oxo-2,3-dihydro-1H-indene-2-carboxylate (3l):**

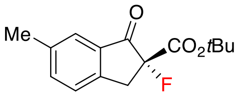


**3l**

*Enantiospecificity:* 98.5% e.s. Prepared following the general procedure on a 75 µmol (19.7 mg of (*S*)-**2l**, 93.4% ee) scale. Purification by silica gel column chromatography using heptanes/EtOAc (20:1 to 9:1) provided the title compound (*R*)-**3l** as a white crystalline solid (15.5 mg, 59 µmol, 78% yield, 91.9% ee). **MP**: 60.3−61.6 °C (EtOAc/heptane). **TLC** (30% EtOAc/heptane): $R_f$ = 0.49 (UV, *p*-anisaldehyde). Analytical data are in accordance with those reported in the literature.[9] **[α]$_D$$^{23.2}$** = +3.4 (*c* 1.00, $CH_2Cl_2$). **$^1$H NMR** (500 MHz, $CDCl_3$, 298 K) δ 7.60 (s, 1H, Ar***H***), 7.48 (d, *J* = 7.8 Hz, 1H, Ar***H***), 7.36 (d, *J* = 7.9 Hz, 1H, Ar***H***), 3.66 (dd, *J* = 17.3, 10.7 Hz, 1H, C***H***H), 3.32 (dd, *J* = 22.9, 17.4 Hz, 1H, CH***H***), 2.40 (s, 3H, ArC***H***$_3$), 1.41 (s, 9H, C***H***$_3$). **$^{13}$C NMR** (126 MHz, $CDCl_3$, 298 K) δ 195.9 (d, *J* = 18.5 Hz, 1C, ***C***=O), 166.5 (d, *J* = 27.7 Hz, 1C, ***C***$O_2$R), 148.5 (d, *J* = 3.9 Hz, 1C, ***C***$_{Ar}$), 138.7 (1C, ***C***$_{Ar}$), 137.9 (1C, ***C***$_{Ar}$), 133.8 (d, *J* = 1.3 Hz, 1C, ***C***$_{Ar}$), 126.2 (d, *J* = 1.5 Hz, 1C, ***C***$_{Ar}$), 125.3 (d, *J* = 1.3 Hz, 1C, ***C***$_{Ar}$), 94.8 (d, *J* = 201.5 Hz, 1C, ***C***qF), 84.1 (1C, ***C***q$Me_3$), 38.1 (d, *J* = 24.1 Hz, 1C, ***C***$H_2$), 27.9 (3C, ***C***$H_3$), 21.2 (1C, Ar***C***$H_3$). **$^{19}$F NMR** (471 MHz, $CDCl_3$, 298 K) δ −163.7 (dd, *J* = 23.4, 11.2 Hz). **HRMS** (ESI-Orbitrap, MeOH) *m/z*: [M + $NH_4$]$^+$ calcd for $C_{15}H_{21}FO_3N$, 282.1500; found, 282.1491. **HPLC**: Chiralpak AD-H (*n*-hexane:*i*-PrOH = 99:1, flow rate 1.0 mL/min, 10 °C, λ = 240 nm), retention times $t_R$(minor) = 12.9 min, $t_R$(major) = 21.4 min.

***tert*-Butyl (*R*)-2-fluoro-6-methoxy-1-oxo-2,3-dihydro-1H-indene-2-carboxylate (3m):**

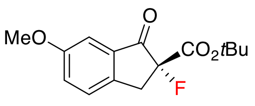


**3m**

*Enantiospecificity:* 99.3% e.s. Prepared following the general procedure on a 70 µmol (19.5 mg of (*S*)-**2m**, 91.1% ee) scale. Purification by silica gel column chromatography using heptanes/EtOAc (15:1 to 8:1) provided the title compound (*R*)-**3m** as a white crystalline solid (16.1 mg, 57 µmol, 82% yield, 90.5% ee). **MP**: 62.4−63.2 °C (EtOAc/heptane). **TLC** (30% EtOAc/heptane): $R_f$ = 0.39 (UV, *p*-anisaldehyde). Analytical data are in accordance with those reported in the literature.[9] **[α]$_D$$^{23.6}$** = +11.0 (*c* 1.00, $CH_2Cl_2$). **$^1$H NMR** (500 MHz, $CDCl_3$, 298 K) δ 7.38 (d, *J* = 8.4 Hz, 1H, Ar***H***), 7.31 – 7.25 (m, 1H, Ar***H***), 7.24 (s, 1H, Ar***H***), 3.86 (s, 3H, OC***H***$_3$), 3.65 (dd, *J* = 17.1, 10.3 Hz, 1H, C***H***H), 3.32 (dd, *J* =

22.6, 17.1 Hz, 1H, CH***H***), 1.44 (s, 9H, ***C***$H_3$). **$^{13}$C NMR** (126 MHz, $CDCl_3$, 298 K) δ 195.9 (d, *J* = 18.5 Hz, 1C, ***C***=O), 166.4 (d, *J* = 27.8 Hz, 1C, ***C***$O_2$R), 160.1 (1C, ***C***$_{Ar}$), 144.1 (d, *J* = 3.9 Hz, 1C, ***C***$_{Ar}$), 134.8 (d, *J* = 1.3 Hz, 1C, ***C***$_{Ar}$), 127.3 (d, *J* = 1.3 Hz, 1C, ***C***$_{Ar}$), 126.0 (1C, ***C***$_{Ar}$), 106.4 (d, *J* = 1.2 Hz, 1C, ***C***$_{Ar}$), 95.1 (d, *J* = 201.9 Hz, 1C, ***C***qF), 84.2 (1C, ***C***q$Me_3$), 55.8 (1C, O***C***$H_3$), 37.8 (d, *J* = 24.0 Hz, 1C, ***C***$H_2$), 27.9 (3C, ***C***$H_3$). **$^{19}$F NMR** (471 MHz, $CDCl_3$, 298 K) δ −163.5 (dd, *J* = 22.6, 10.3 Hz). **HRMS** (ESI-Orbitrap, MeOH) *m*/*z*: $[M + NH_4]^+$ calcd for $C_{15}H_{21}FO_4N$, 298.1449; found, 298.1439. **HPLC**: Chiralpak AD-H (*n*-hexane:*i*-PrOH = 99:1, flow rate 1.0 mL/min, 10 °C, λ = 250 nm), retention times $t_R$(minor) = 17.8 min, $t_R$(major) = 19.4 min.

***tert*-Butyl (*R*)-5,7-dichloro-2-fluoro-1-oxo-2,3-dihydro-1H-indene-2-carboxylate (3n):**

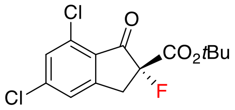

**3n**

*Enantiospecificity:* >99.8% e.s. Prepared following the general procedure on a 40 µmol (12.7 mg of (*S*)-**2n**, 75.5% ee) scale. Purification by silica gel column chromatography using heptanes/EtOAc (20:1 to 10:1) provided the title compound (*R*)-**3n** as a white amorphous solid (6.3 mg, 20 µmol, 49% yield, 75.5% ee). **TLC** (30% EtOAc/heptane): $R_f$ = 0.50 (UV, *p*-anisaldehyde). $[\alpha]_D^{23.7}$ = +2.0 (*c* 0.55, $CH_2Cl_2$). $^1$H NMR (500 MHz, $CDCl_3$, 298 K) δ 7.42 (s, 1H, Ar***H***), 7.39 (s, 1H, Ar***H***), 3.67 (dd, *J* = 17.7, 11.1 Hz, 1H, C***H***H), 3.34 (dd, *J* = 22.5, 17.8 Hz, 1H, CH***H***), 1.46 (s, 9H, C***H***$_3$). **$^{13}$C NMR** (126 MHz, $CDCl_3$, 298 K) δ 191.6 (d, *J* = 19.0 Hz, 1C, ***C***=O), 165.7 (d, *J* = 27.3 Hz, 1C, ***C***$O_2$R), 153.9 (d, *J* = 3.8 Hz, 1C, ***C***$_{Ar}$), 143.0 (1C, ***C***$_{Ar}$), 134.6 (1C, ***C***$_{Ar}$), 130.5 (1C, ***C***$_{Ar}$), 128.7 (1C, ***C***$_{Ar}$), 125.3 (1C, ***C***$_{Ar}$), 94.4 (d, *J* = 203.7 Hz, 1C, ***C***qF), 84.9 (1C, ***C***q$Me_3$), 37.5 (d, *J* = 24.4 Hz, 1C, ***C***$H_2$), 28.0 (3C, ***C***$H_3$). **$^{19}$F NMR** (471 MHz, $CDCl_3$, 298 K) δ −161.8 (dd, *J* = 22.5, 11.2 Hz). **HRMS** (ESI-Orbitrap, MeOH) *m*/*z*: $[M + NH_4]^+$ calcd for $C_{14}H_{17}Cl_2FO_3N$, 336.0564; found, 336.0575. **HPLC**: YMC Chiral ART Amylose SA (*n*-hexane:*i*-PrOH = 99:1, flow rate 1.0 mL/min, 10 °C, λ = 220 nm), retention times $t_R$(major) = 10.4 min, $t_R$(minor) = 24.1 min.

## 3. Synthesis of Racemic Fluorinated Products:

### General Procedure

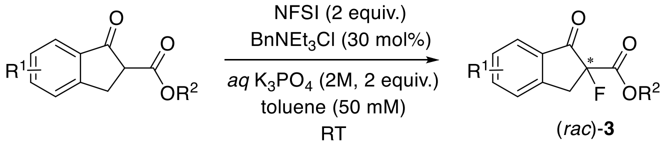


Racemic fluorinated products (**3**) were prepared according to a modified literature procedure[8]: Aqueous $K_3PO_4$ (2M, 2 equiv.) was added to a mixture of β-ketoester and benzyltriethylammonium chloride (BTEAC, 30 mol%) in toluene (50 mM concerning substrate) at room temperature under an atmosphere of argon. Then, *N*-fluorobenzene sulfonimide (NFSI, 2 equiv.) was added portionwise over 5 min under heavy stirring. The reaction was monitored by TLC using heptanes/EtOAc (7:3) as mobile phase. After stirring for 18 h, the resulting mixture was quenched by addition of *aq.* sat. $NH_4Cl$ (25 mL per mmol substrate), the organic phase was separated and the aqueous phase was extracted

with dichloromethane (3 × 50 mL per mmol substrate). The combined organic phases were dried over anhydrous $Na_2SO_4$, filtered and the solvents were removed *in vacuo*. The residue was purified by silica gel column chromatography (eluent: heptanes/EtOAc).

# 4. Copies of NMR Spectra of Deoxyfluorination Products:

NMR spectra of compound **3a**

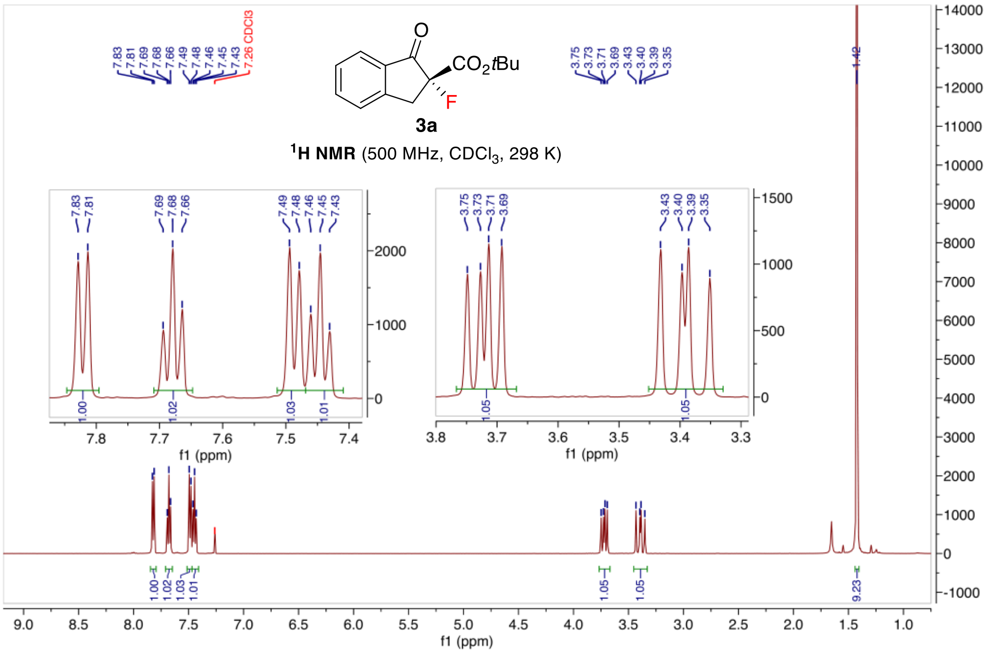


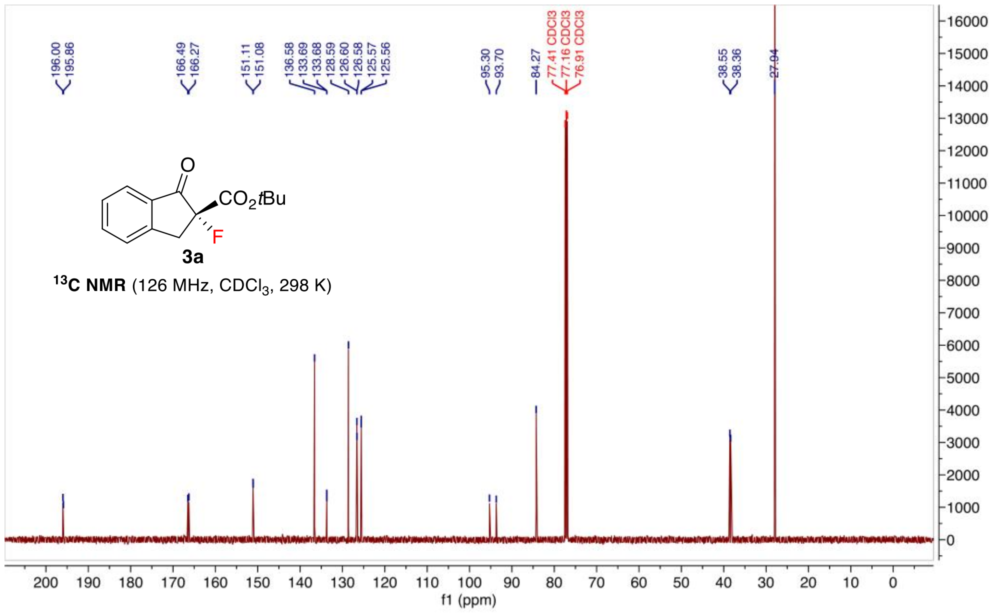


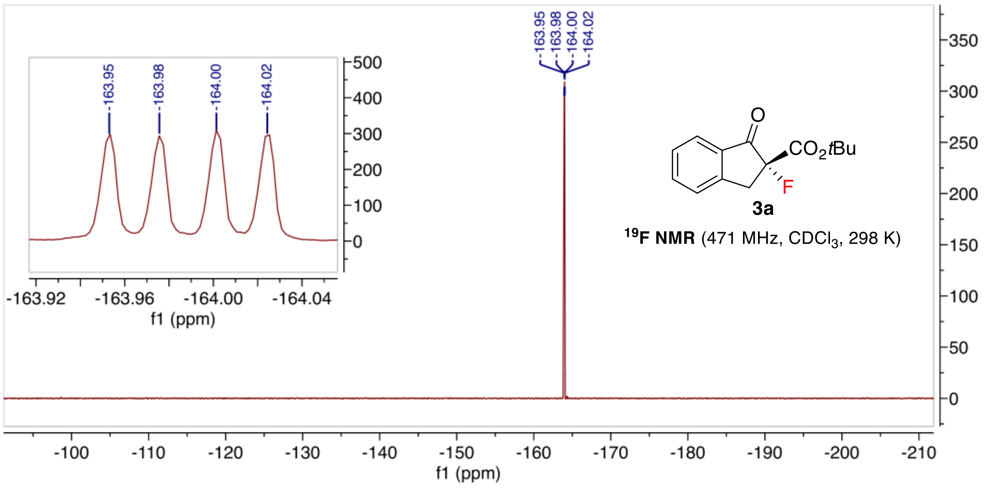

# NMR spectra of compound **3b**

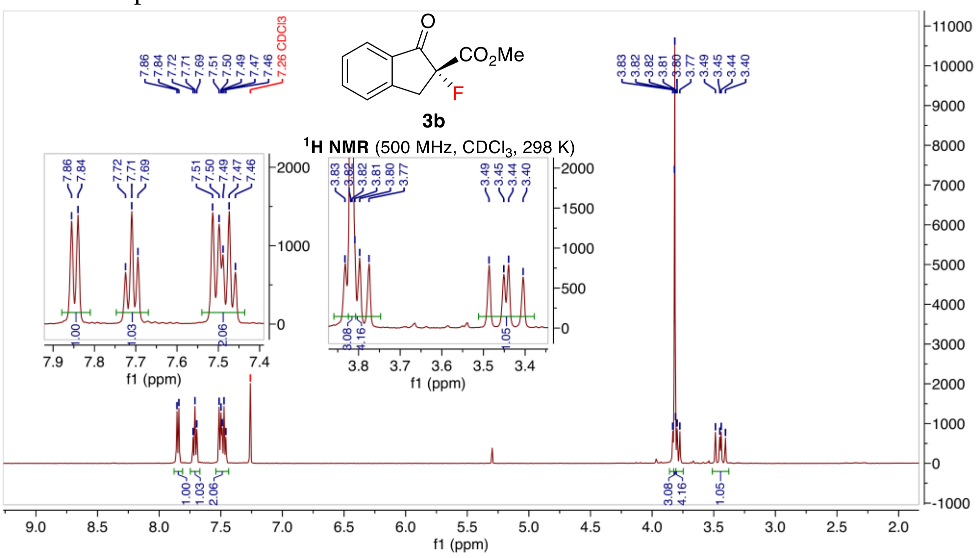


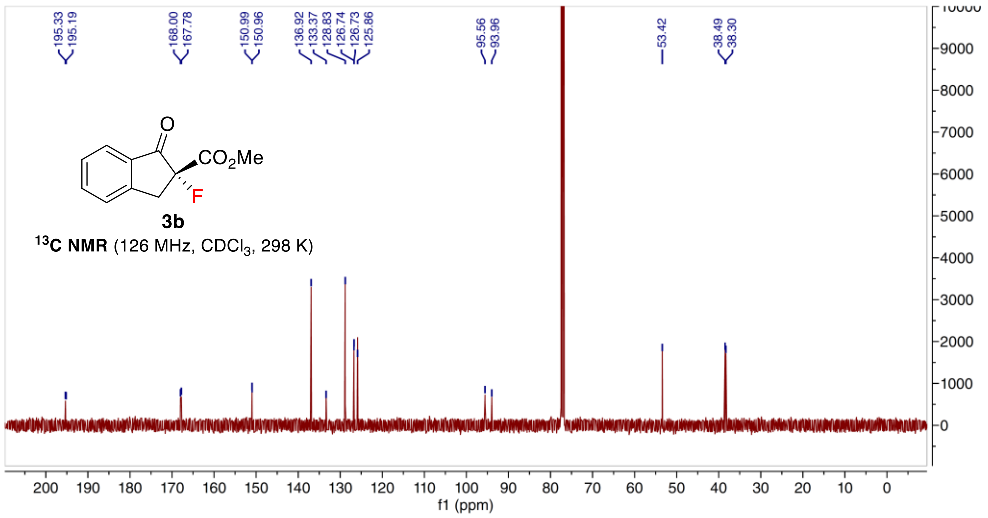


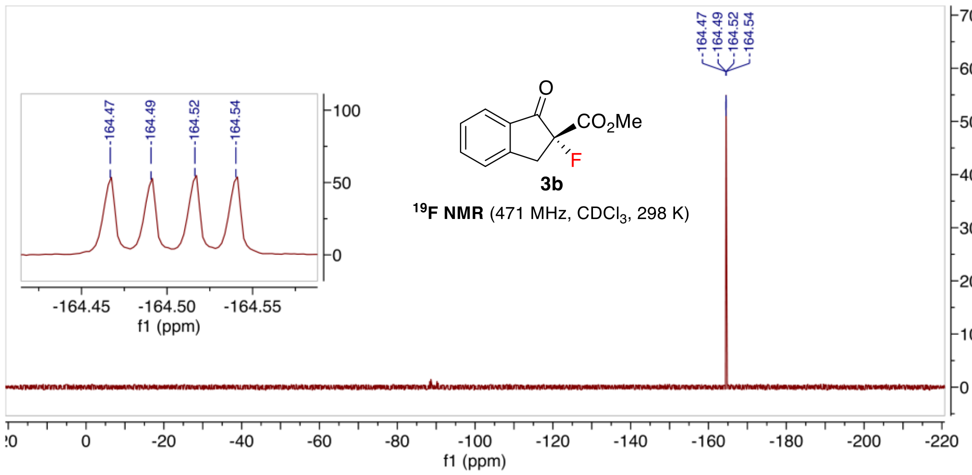

# NMR spectra of compound **3c**

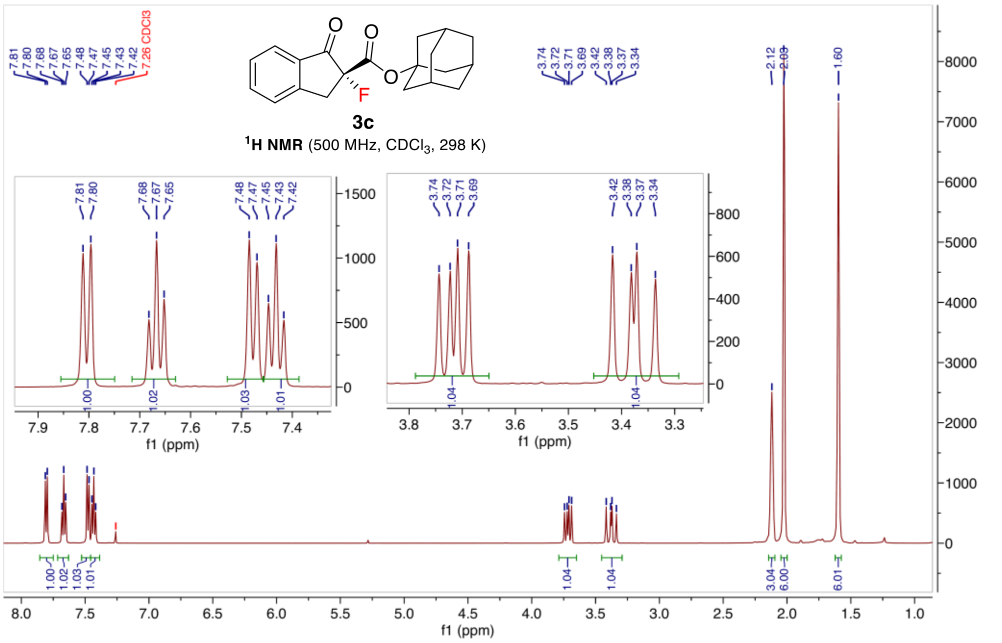


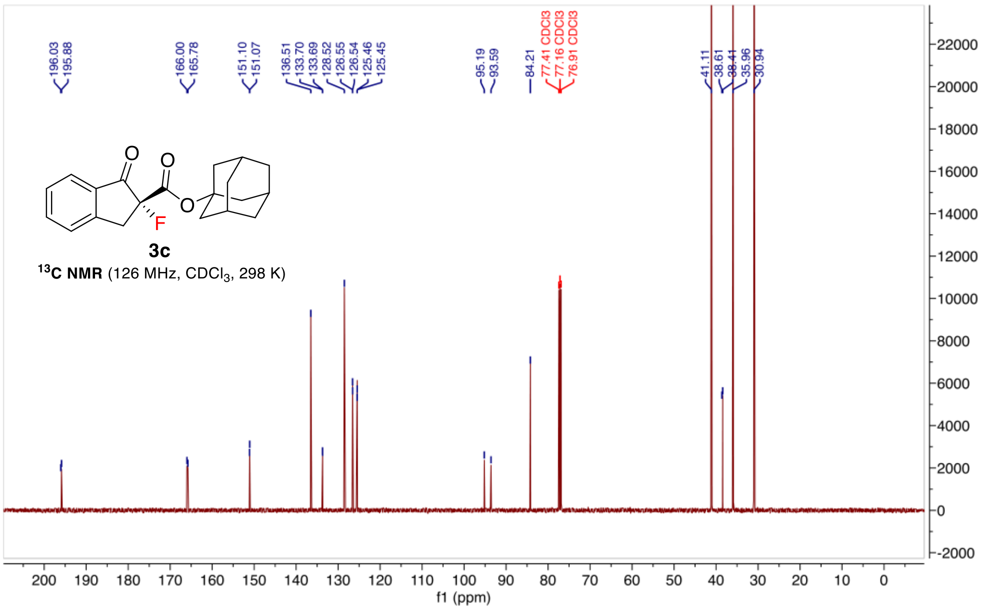


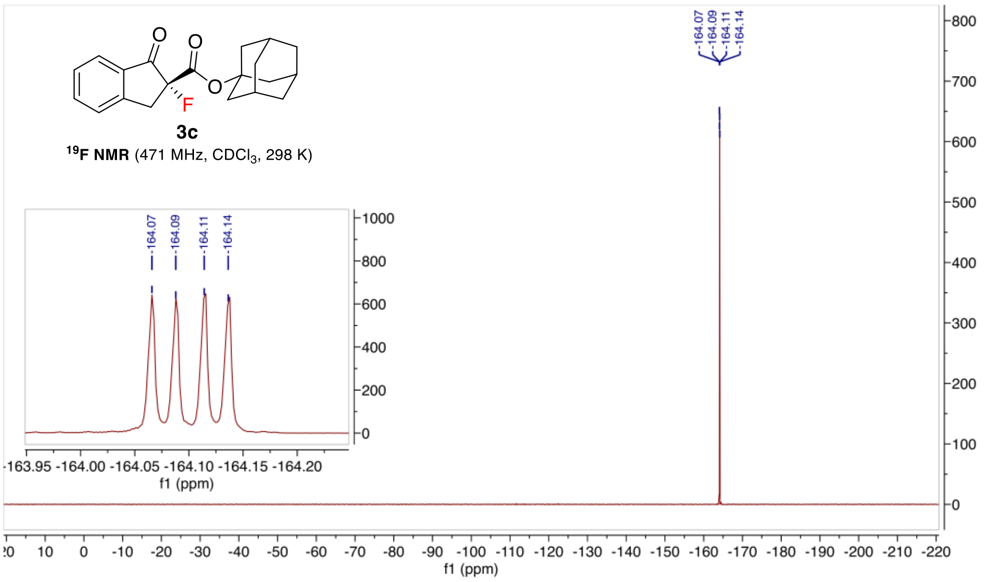

NMR spectra of compound **3d**

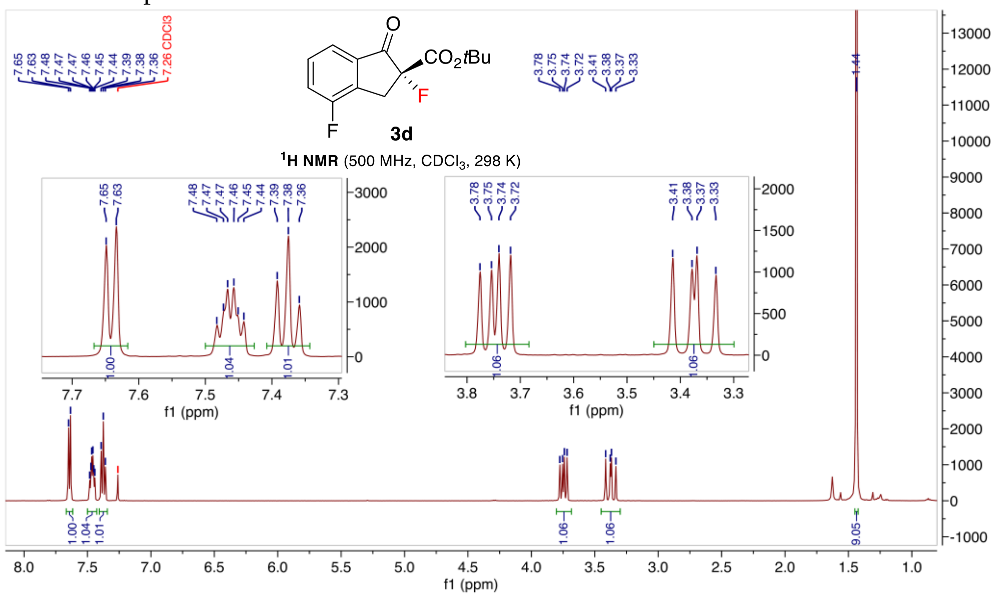


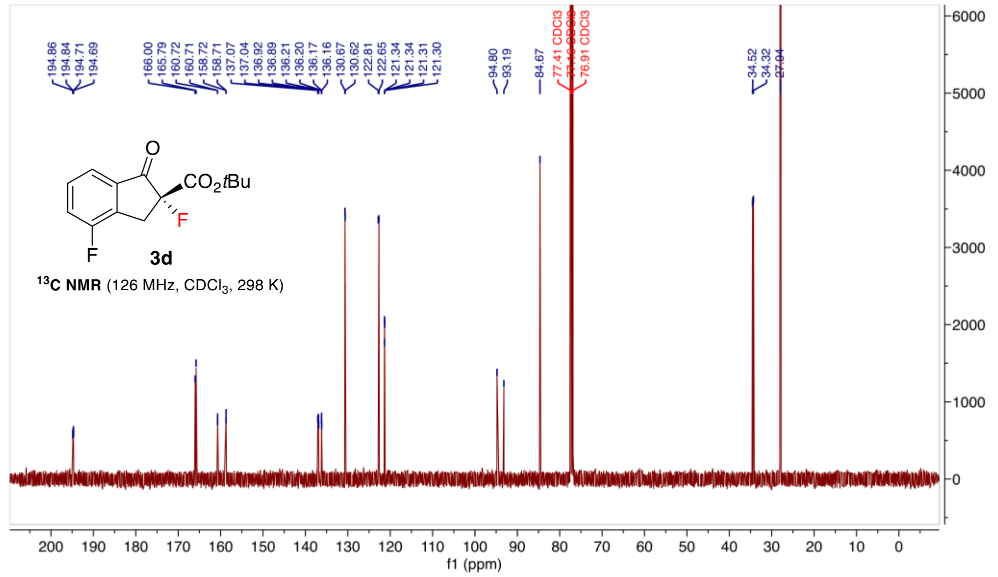


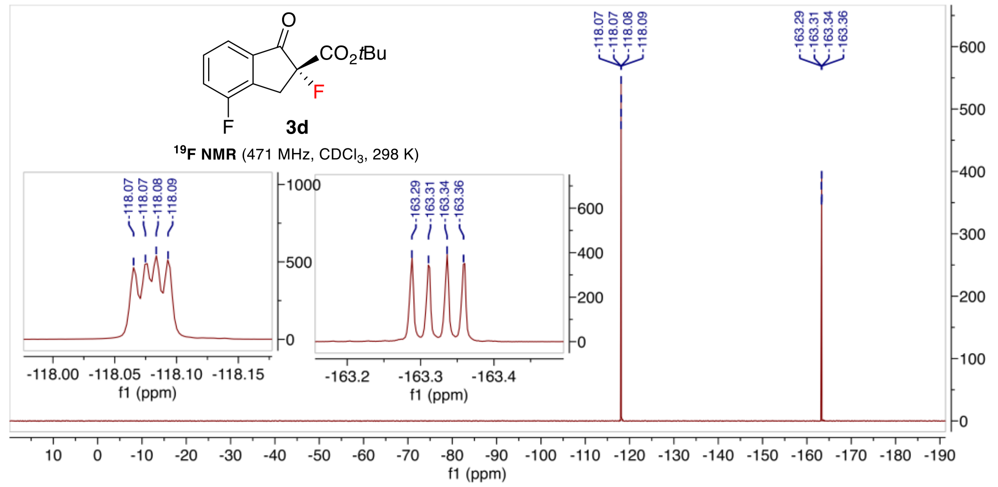

NMR spectra of compound **3e**

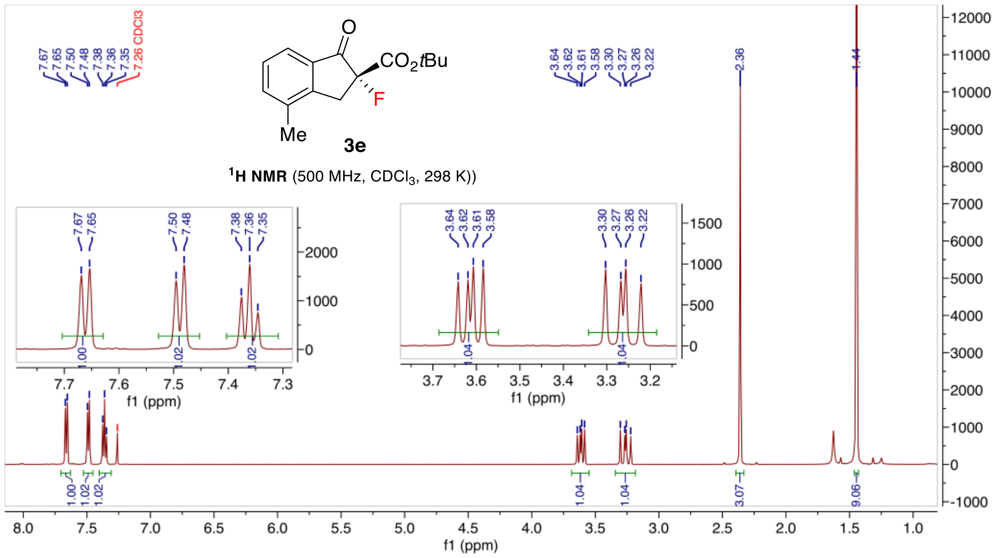


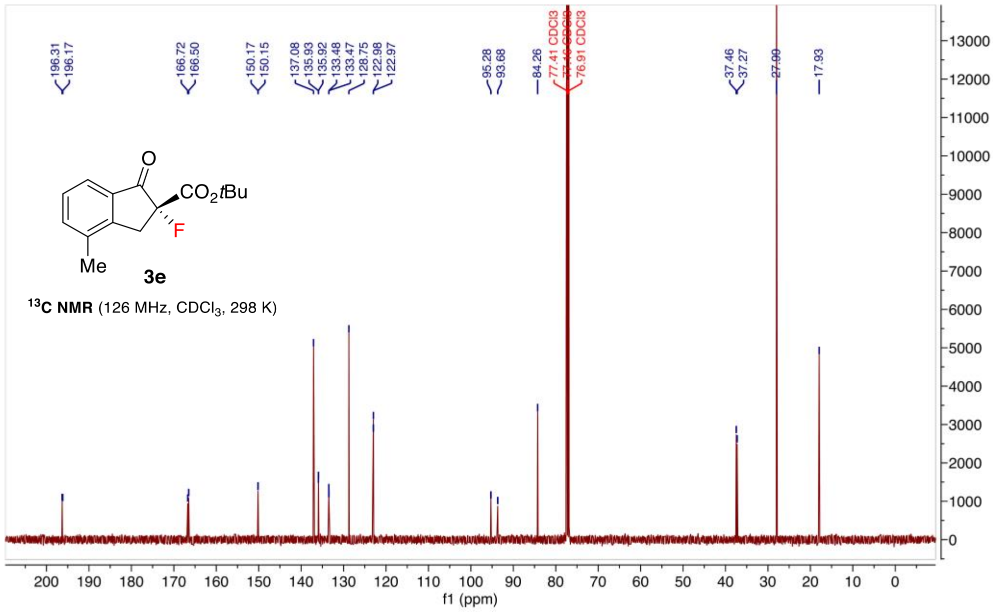


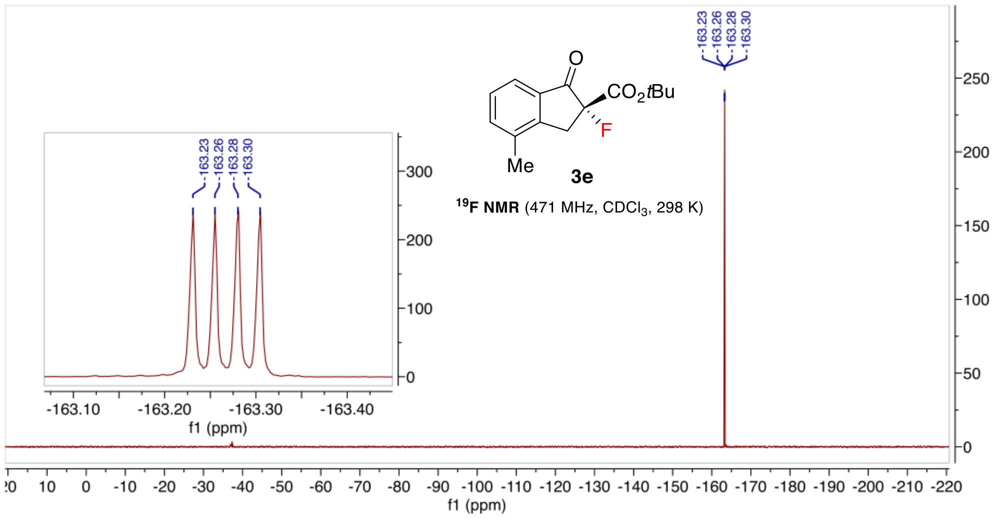

# NMR spectra of compound **3f**

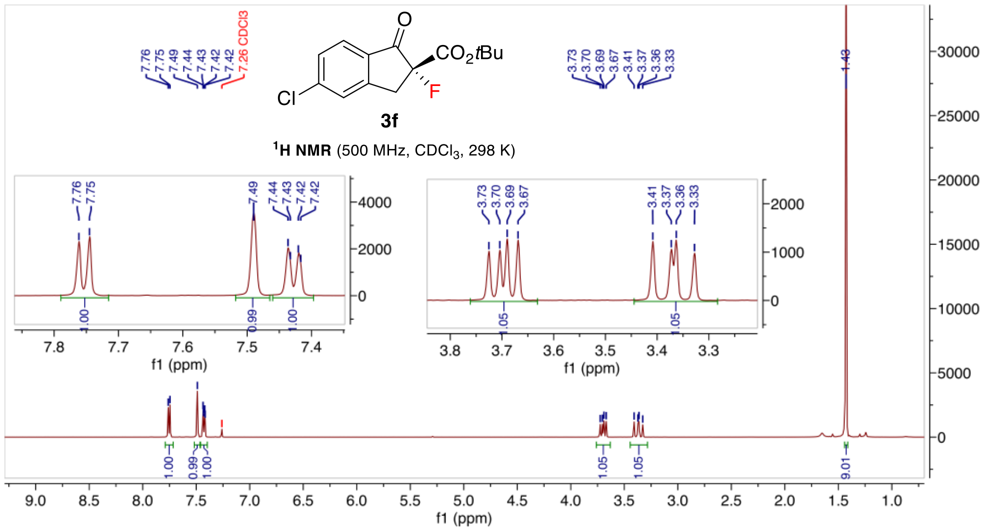


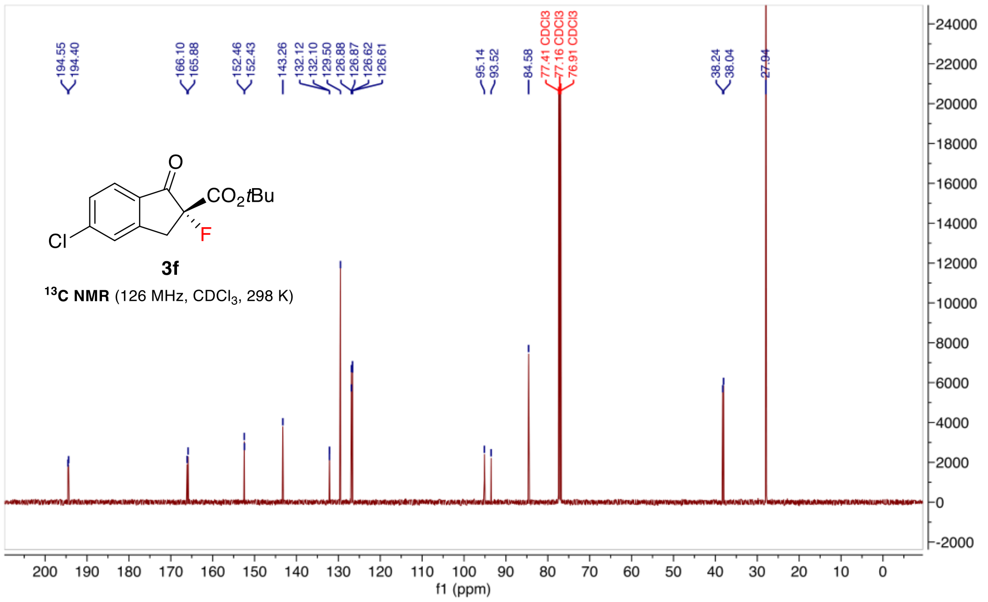


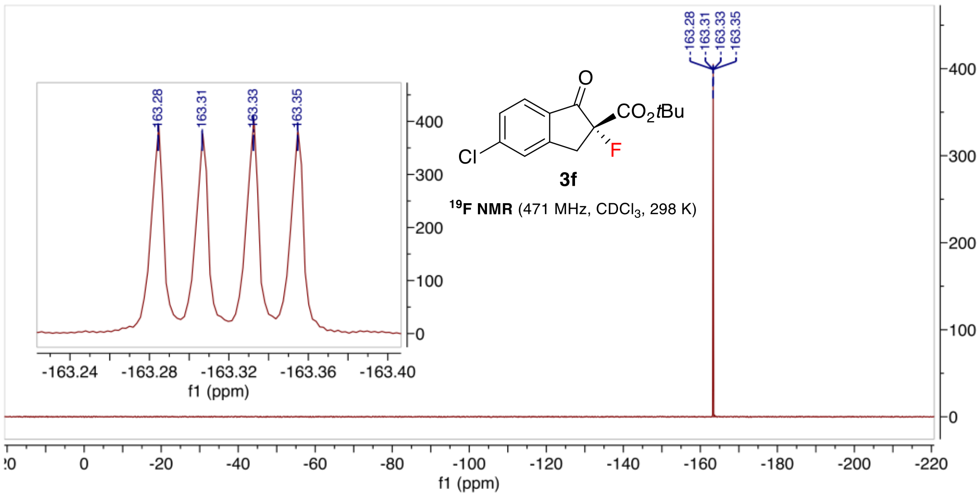

NMR spectra of compound **3g**

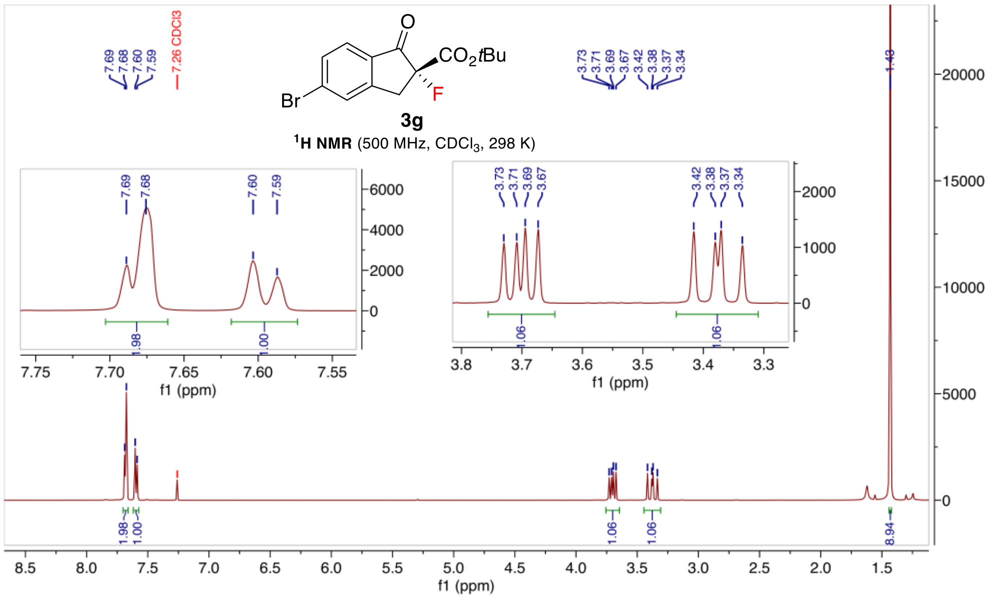


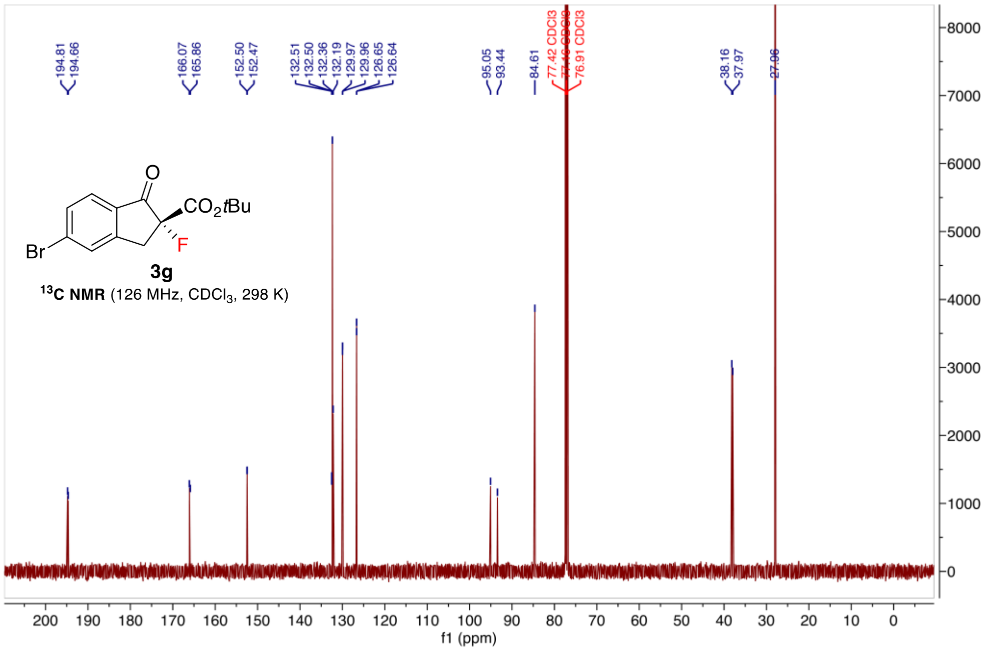


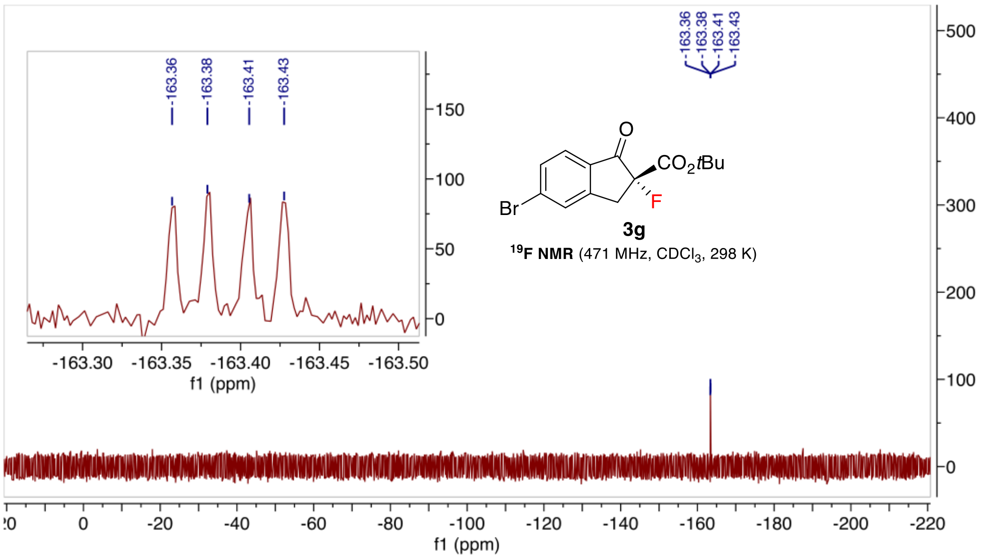

# NMR spectra of compound **3h**

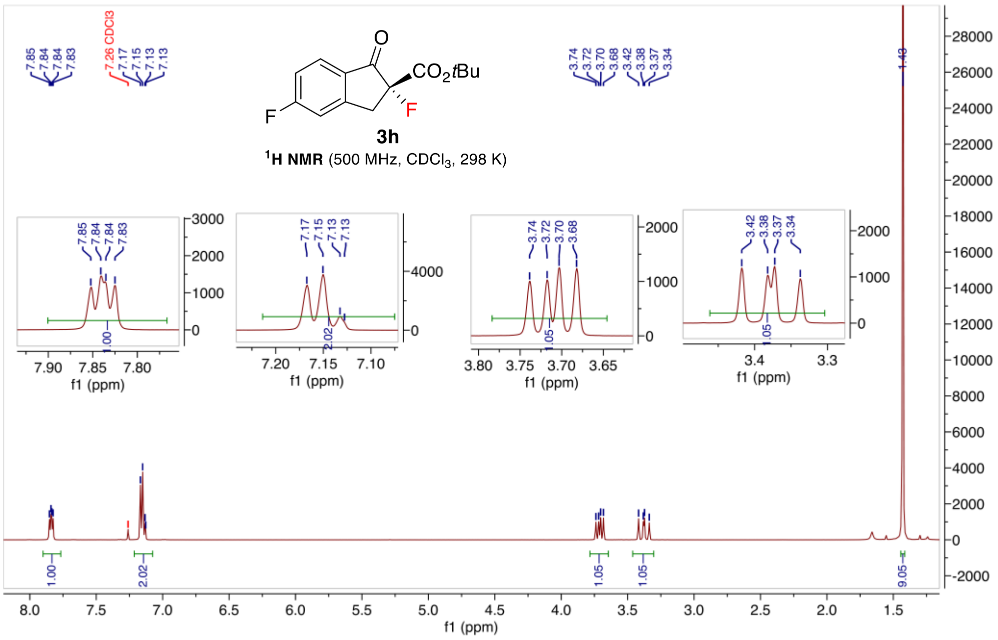


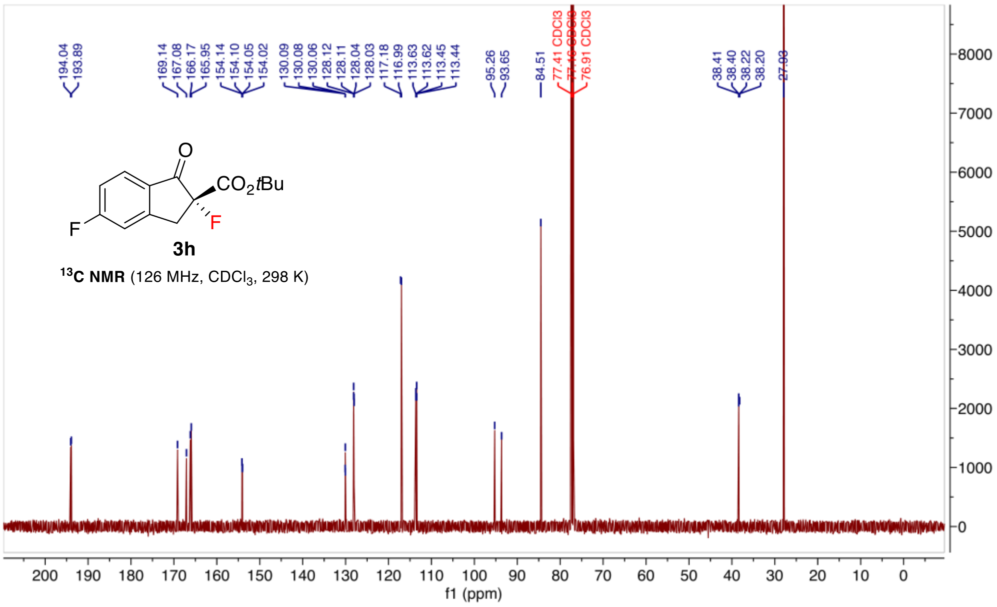


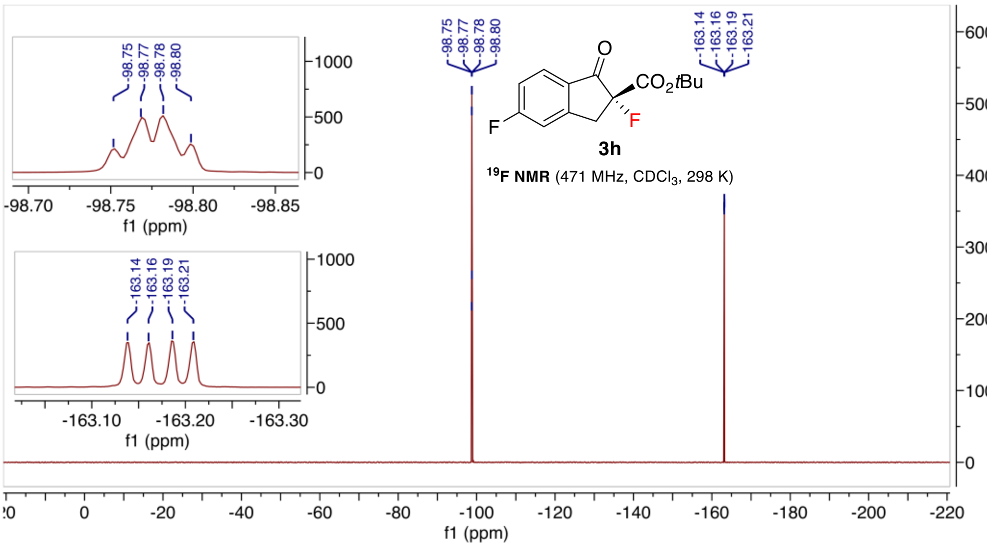

NMR spectra of compound **3i**

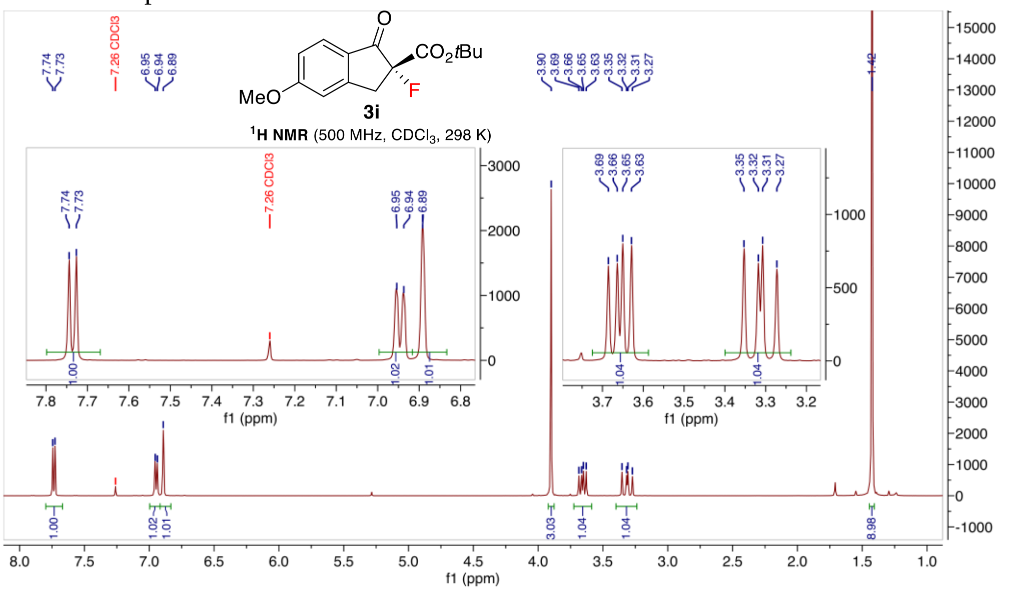


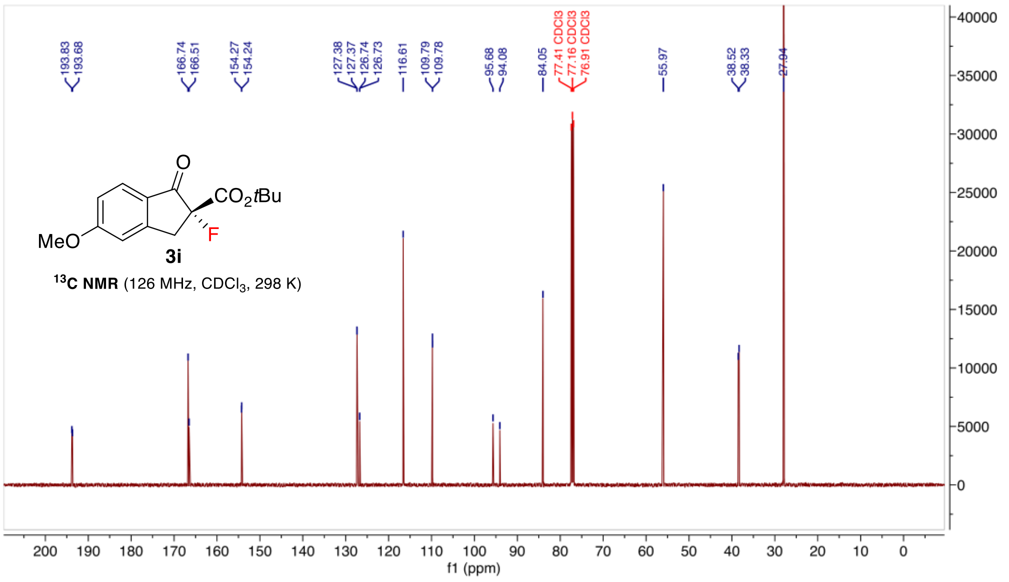


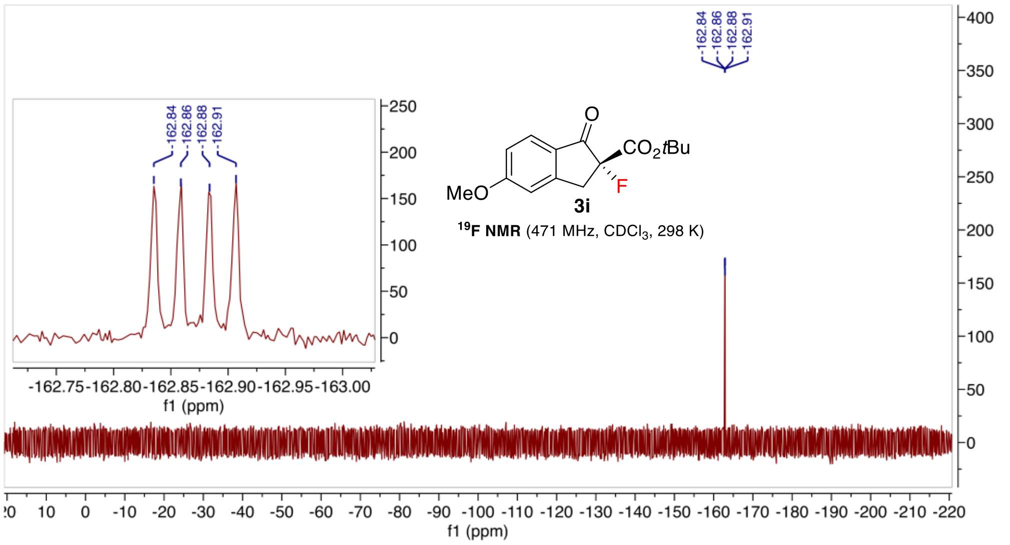

# NMR spectra of compound **3j**

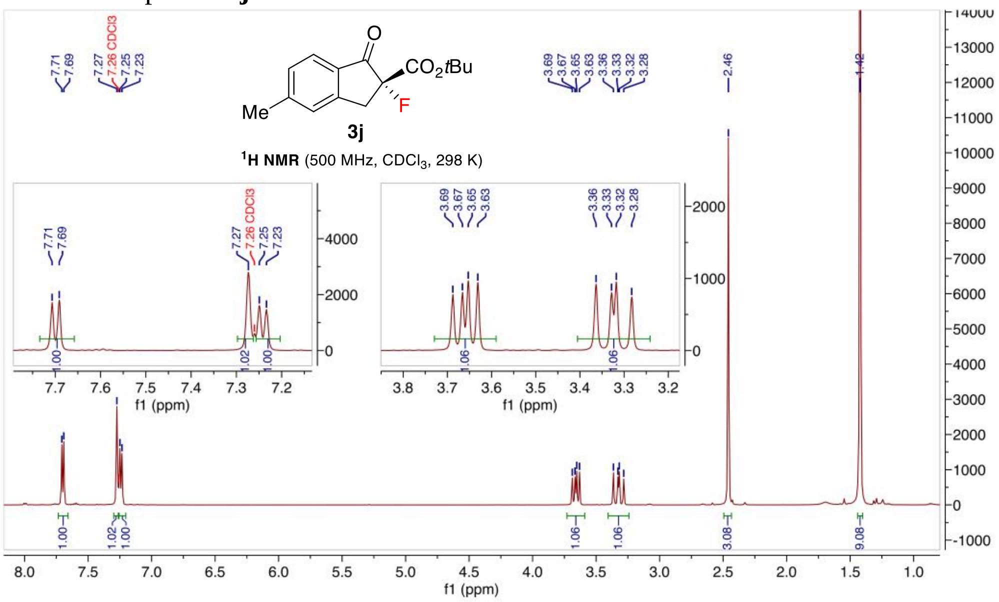


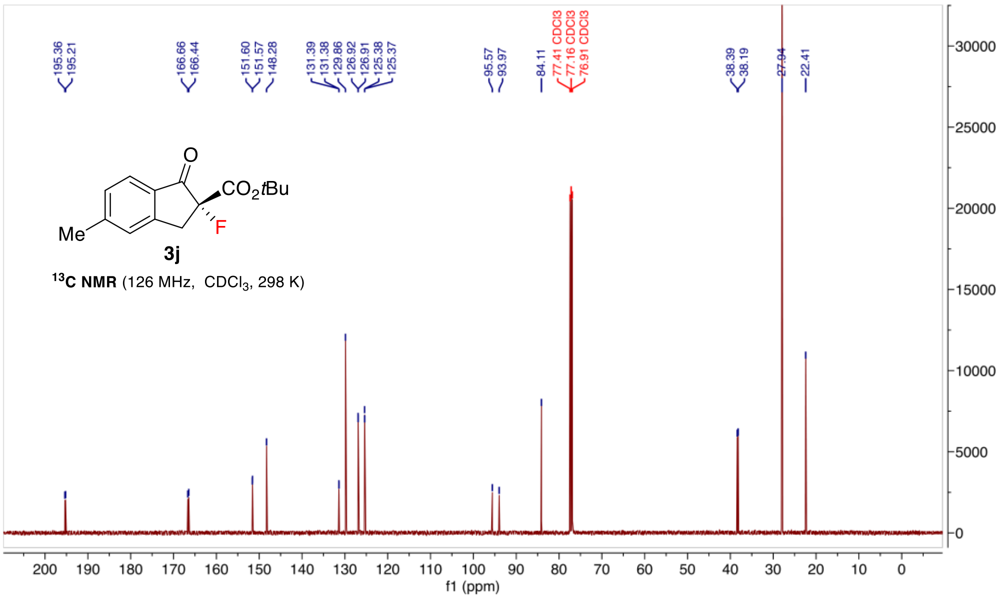


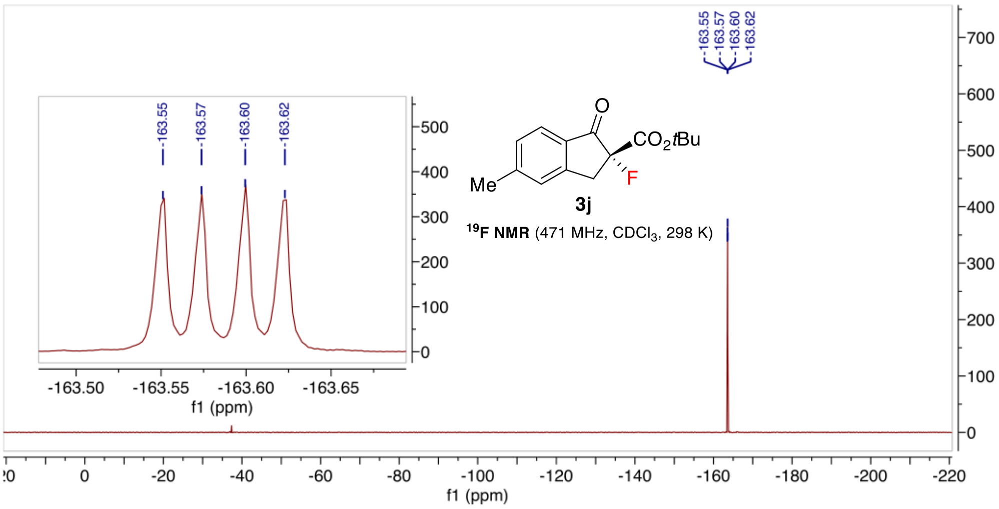

NMR spectra of compound **3k**

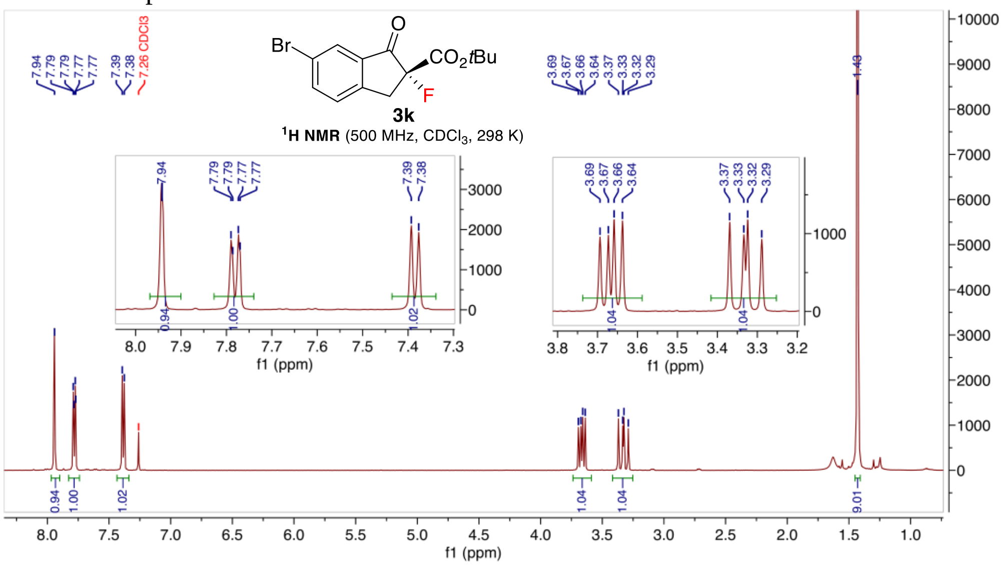

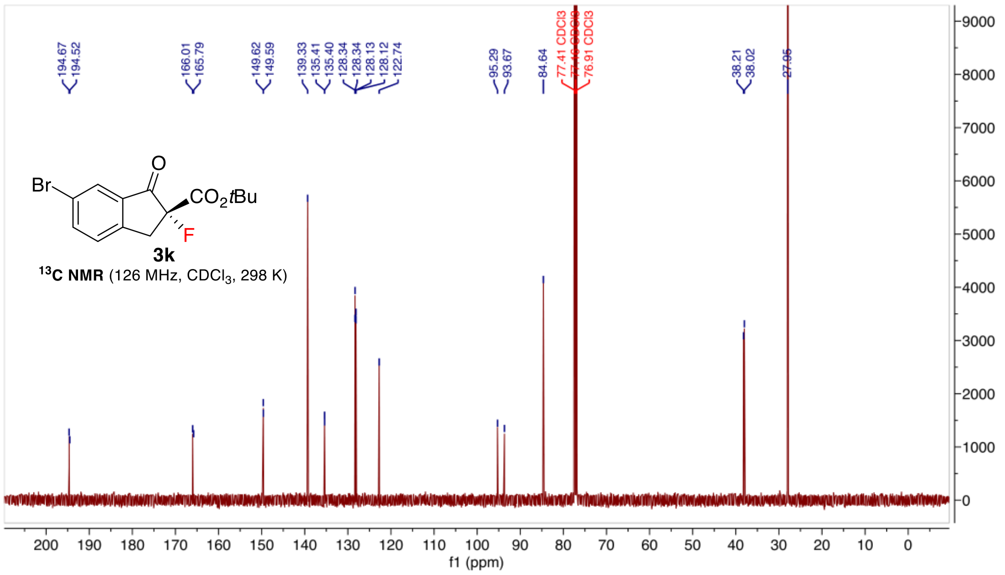

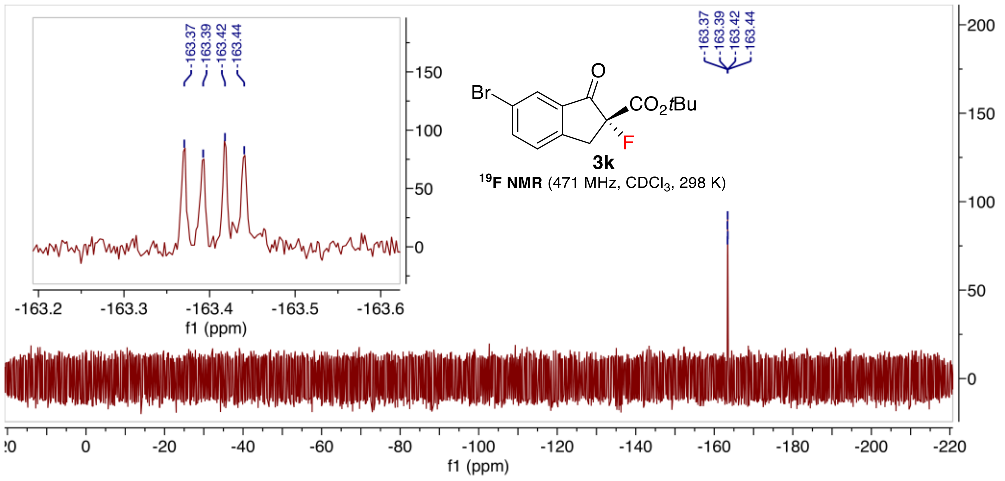

NMR spectra of compound **3l**

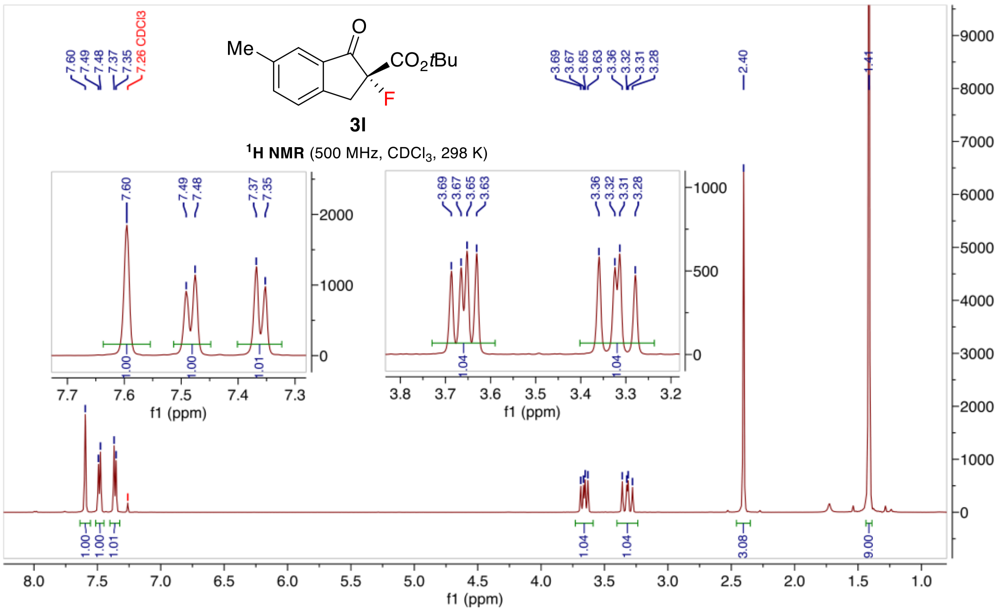


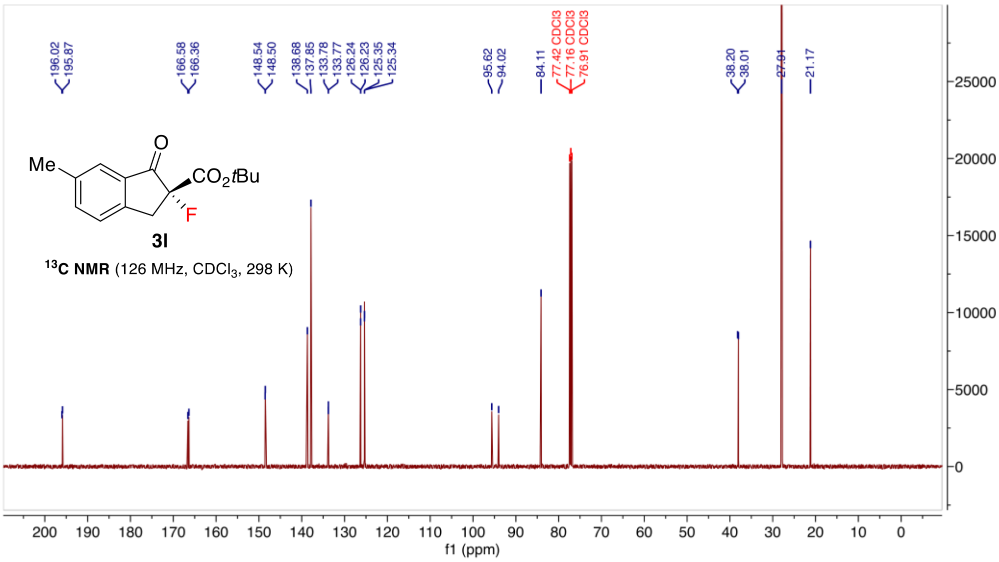


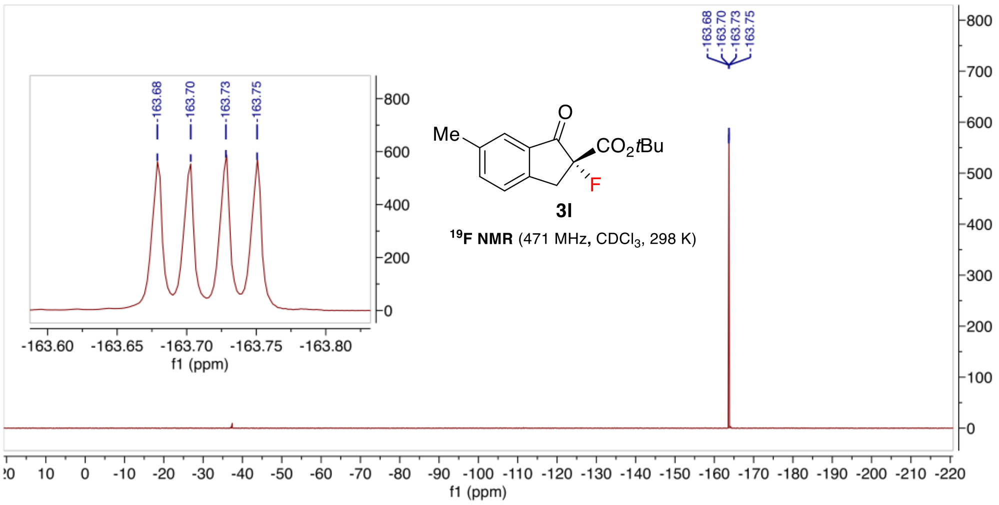

# NMR spectra of compound **3m**

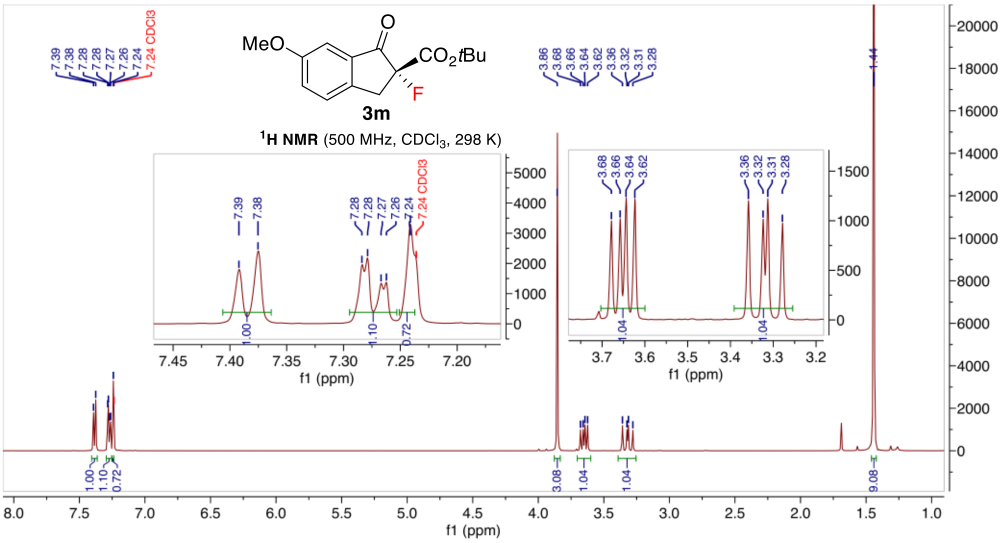


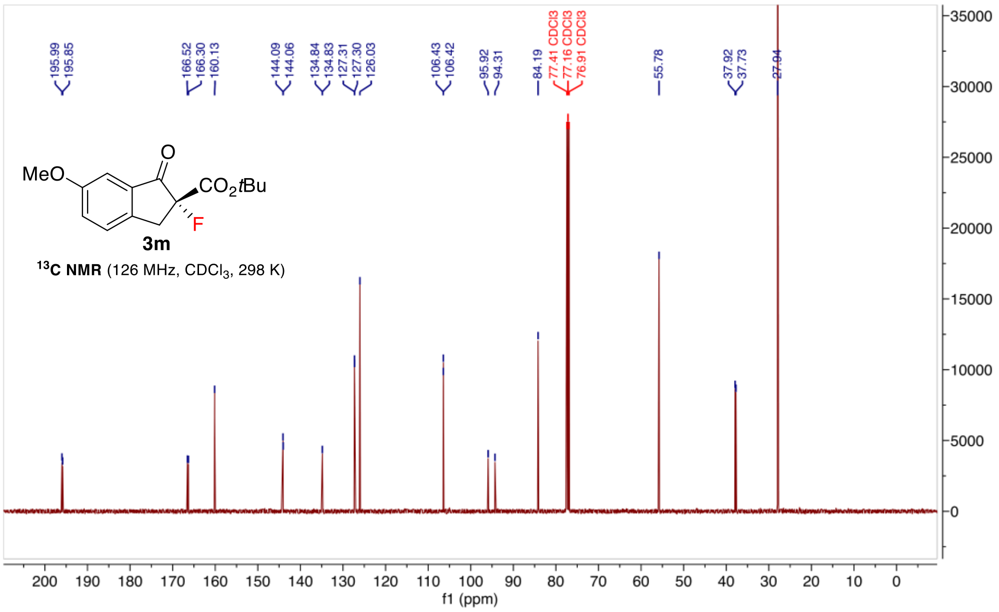


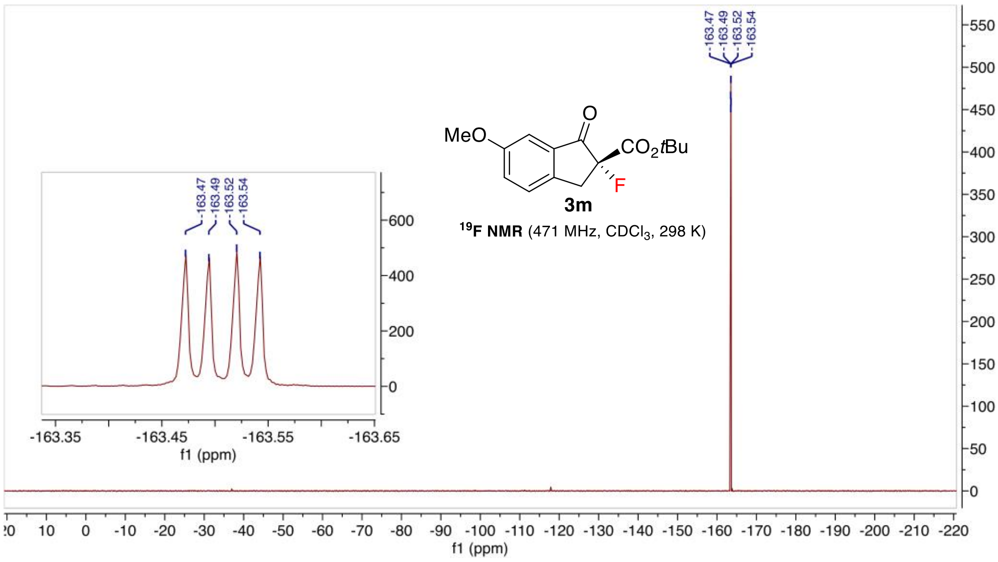

NMR spectra of compound **3n**

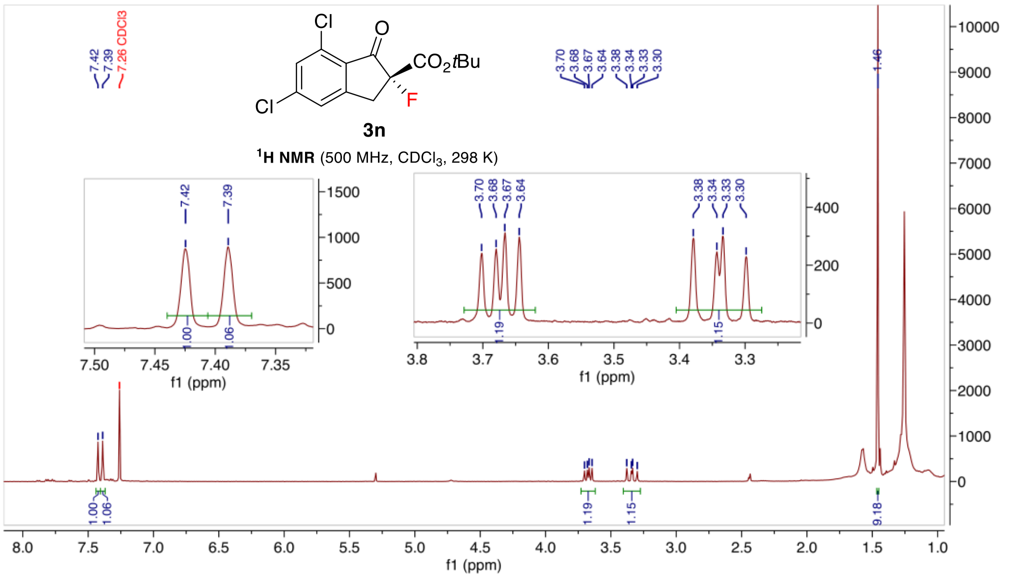


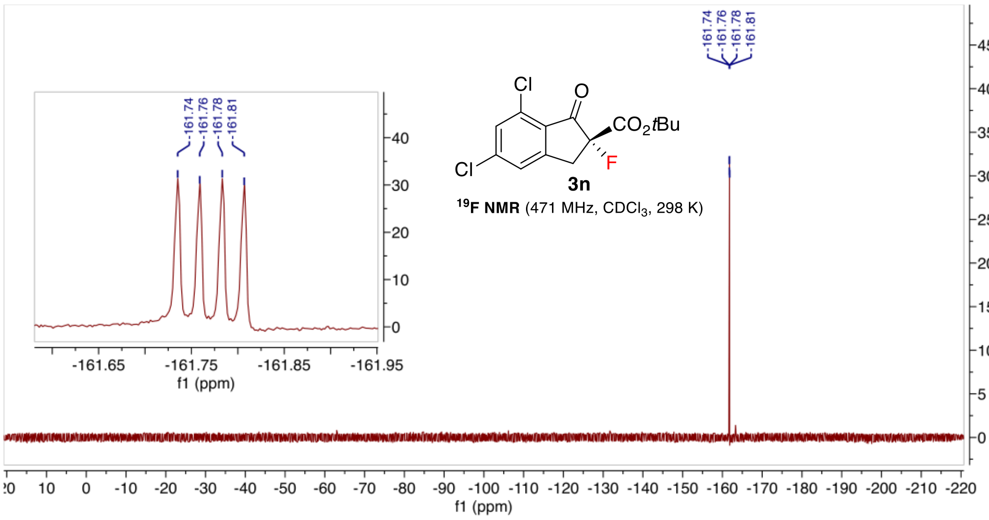

# 5. HPLC Traces of Hydroxylation/Deoxyfluorination Products:

HPLC chromatogram of (*rac*)-**2a**:

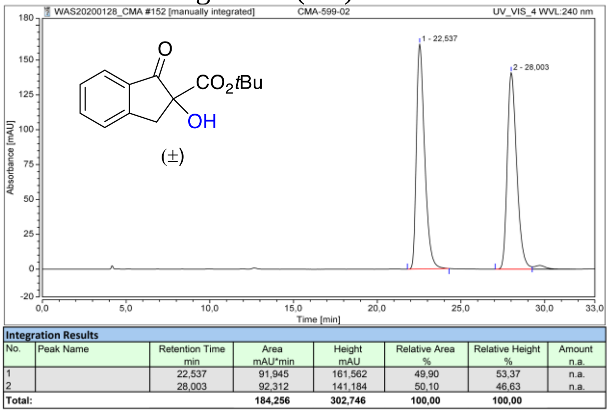


Integration Results

| No. | Peak Name | Retention Time min | Area mAU*min | Height mAU | Relative Area % | Relative Height % | Amount n.a. |
|---|---|---|---|---|---|---|---|
| 1 | | 22,537 | 91,945 | 161,562 | 49,90 | 53,37 | n.a. |
| 2 | | 28,003 | 92,312 | 141,184 | 50,10 | 46,63 | n.a. |
| Total: | | | 184,256 | 302,746 | 100,00 | 100,00 | |

HPLC chromatogram of (*rac*)-**3a**:

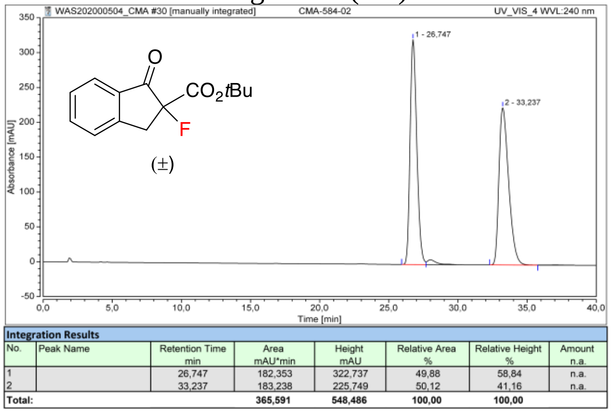


Integration Results

| No. | Peak Name | Retention Time min | Area mAU*min | Height mAU | Relative Area % | Relative Height % | Amount n.a. |
|---|---|---|---|---|---|---|---|
| 1 | | 26,747 | 182,353 | 322,737 | 49,88 | 58,84 | n.a. |
| 2 | | 33,237 | 183,238 | 225,749 | 50,12 | 41,16 | n.a. |
| Total: | | | 365,591 | 548,486 | 100,00 | 100,00 | |

HPLC chromatogram of enantioenriched **2a**:

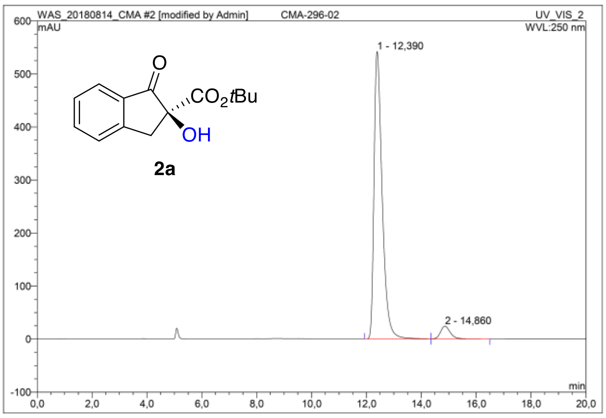


| No. | Ret.Time min | Peak Name | Height mAU | Area mAU*min | Rel.Area % | Amount | Type |
|---|---|---|---|---|---|---|---|
| 1 | 12,39 | n.a. | 542,205 | 186,382 | 94,83 | n.a. | BM * |
| 2 | 14,86 | n.a. | 24,208 | 10,151 | 5,17 | n.a. | MB* |
| Total: | | | 566,413 | 196,533 | 100,00 | 0,000 | |

HPLC chromatogram of enantioenriched **3a**:

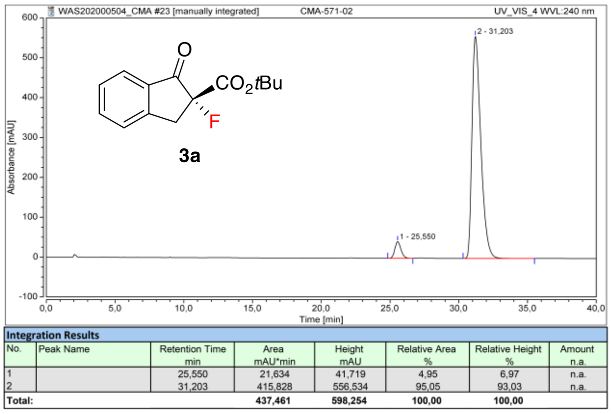


Integration Results

| No. | Peak Name | Retention Time min | Area mAU*min | Height mAU | Relative Area % | Relative Height % | Amount n.a. |
|---|---|---|---|---|---|---|---|
| 1 | | 25,550 | 21,634 | 41,719 | 4,95 | 6,97 | n.a. |
| 2 | | 31,203 | 415,828 | 556,534 | 95,05 | 93,03 | n.a. |
| Total: | | | 437,461 | 598,254 | 100,00 | 100,00 | |

---

HPLC chromatogram of (*rac*)-**2b**:

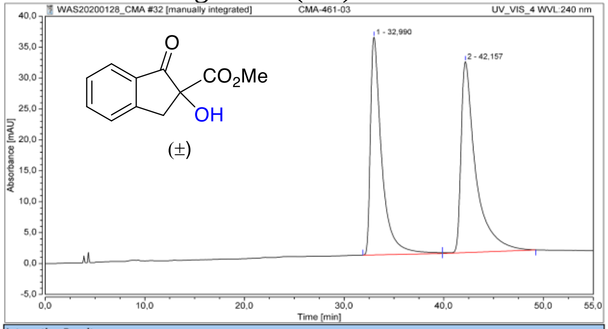


Integration Results

| No. | Peak Name | Retention Time min | Area mAU*min | Height mAU | Relative Area % | Relative Height % | Amount n.a. |
|---|---|---|---|---|---|---|---|
| 1 | | 32,990 | 46,202 | 35,227 | 46,62 | 53,27 | n.a. |
| 2 | | 42,157 | 52,912 | 30,904 | 53,38 | 46,73 | n.a. |
| Total: | | | 99,114 | 66,131 | 100,00 | 100,00 | |

HPLC chromatogram of (*rac*)-**3b**:

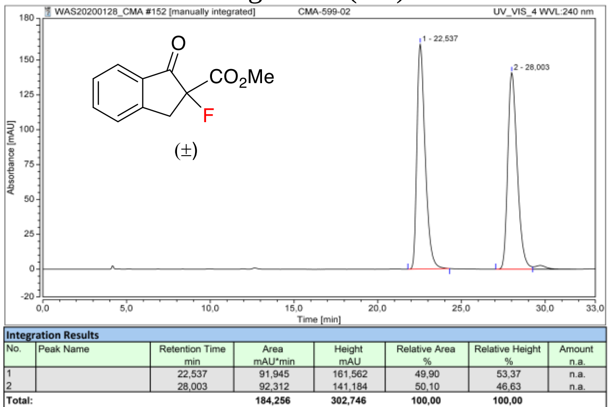


Integration Results

| No. | Peak Name | Retention Time min | Area mAU*min | Height mAU | Relative Area % | Relative Height % | Amount n.a. |
|---|---|---|---|---|---|---|---|
| 1 | | 22,537 | 91,945 | 161,562 | 49,90 | 53,37 | n.a. |
| 2 | | 28,003 | 92,312 | 141,184 | 50,10 | 46,63 | n.a. |
| Total: | | | 184,256 | 302,746 | 100,00 | 100,00 | |

HPLC chromatogram of enantioenriched **2b**:

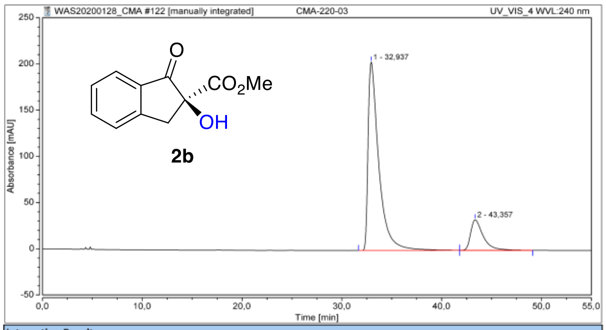


Integration Results

| No. | Peak Name | Retention Time min | Area mAU*min | Height mAU | Relative Area % | Relative Height % | Amount n.a. |
|---|---|---|---|---|---|---|---|
| 1 | | 32,937 | 254,356 | 203,877 | 84,01 | 86,06 | n.a. |
| 2 | | 43,357 | 48,408 | 33,021 | 15,99 | 13,94 | n.a. |
| Total: | | | 302,764 | 236,898 | 100,00 | 100,00 | |

HPLC chromatogram of enantioenriched **3b**:

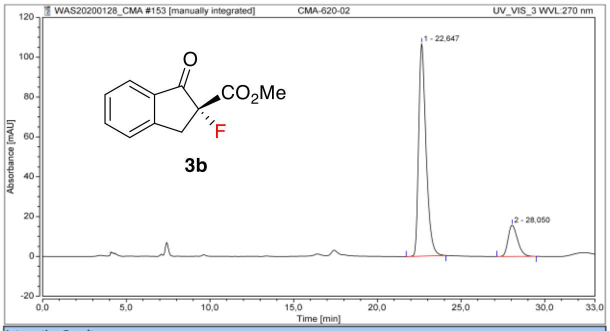


Integration Results

| No. | Peak Name | Retention Time min | Area mAU*min | Height mAU | Relative Area % | Relative Height % | Amount n.a. |
|---|---|---|---|---|---|---|---|
| 1 | | 22,647 | 54,698 | 106,683 | 84,15 | 87,18 | n.a. |
| 2 | | 28,050 | 10,301 | 15,694 | 15,85 | 12,82 | n.a. |
| Total: | | | 64,999 | 122,377 | 100,00 | 100,00 | |

HPLC chromatogram of (*rac*)-**2c**:

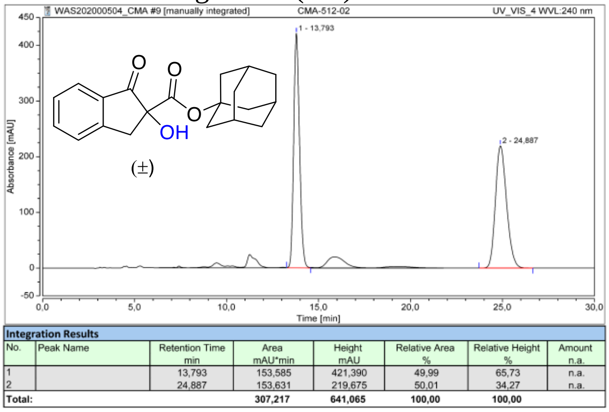


| Integration Results | | | | | | | |
|---|---|---|---|---|---|---|---|
| No. | Peak Name | Retention Time min | Area mAU*min | Height mAU | Relative Area % | Relative Height % | Amount n.a. |
| 1 | | 13,793 | 153,585 | 421,390 | 49,99 | 65,73 | n.a. |
| 2 | | 24,887 | 153,631 | 219,675 | 50,01 | 34,27 | n.a. |
| Total: | | | 307,217 | 641,065 | 100,00 | 100,00 | |

HPLC chromatogram of (*rac*)-**3c**:

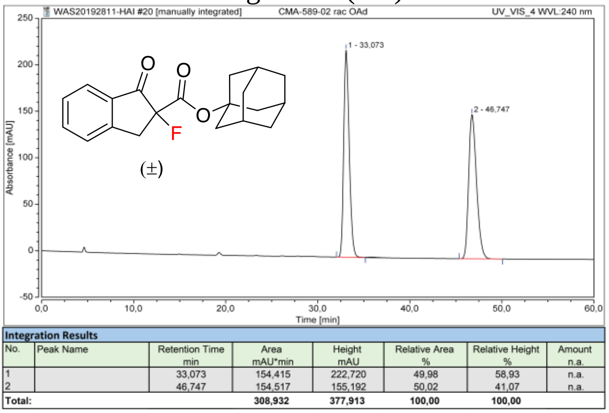


| Integration Results | | | | | | | |
|---|---|---|---|---|---|---|---|
| No. | Peak Name | Retention Time min | Area mAU*min | Height mAU | Relative Area % | Relative Height % | Amount n.a. |
| 1 | | 33,073 | 154,415 | 222,720 | 49,98 | 58,93 | n.a. |
| 2 | | 46,747 | 154,517 | 155,192 | 50,02 | 41,07 | n.a. |
| Total: | | | 308,932 | 377,913 | 100,00 | 100,00 | |

HPLC chromatogram of enantioenriched **2c**:

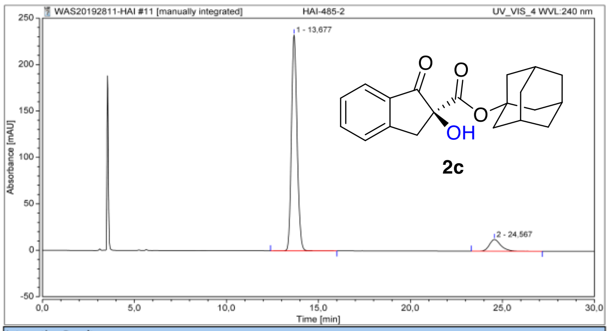


| Integration Results | | | | | | | |
|---|---|---|---|---|---|---|---|
| No. | Peak Name | Retention Time min | Area mAU*min | Height mAU | Relative Area % | Relative Height % | Amount n.a. |
| 1 | | 13,677 | 82,426 | 232,563 | 90,19 | 94,88 | n.a. |
| 2 | | 24,567 | 8,969 | 12,547 | 9,81 | 5,12 | n.a. |
| Total: | | | 91,395 | 245,111 | 100,00 | 100,00 | |

HPLC chromatogram of enantioenriched **3c**:

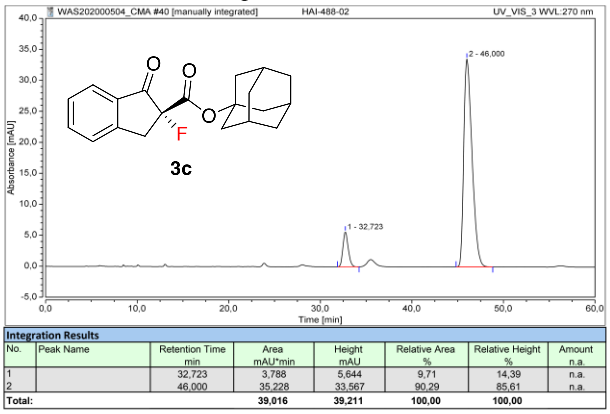


| Integration Results | | | | | | | |
|---|---|---|---|---|---|---|---|
| No. | Peak Name | Retention Time min | Area mAU*min | Height mAU | Relative Area % | Relative Height % | Amount n.a. |
| 1 | | 32,723 | 3,788 | 5,644 | 9,71 | 14,39 | n.a. |
| 2 | | 46,000 | 35,228 | 33,567 | 90,29 | 85,61 | n.a. |
| Total: | | | 39,016 | 39,211 | 100,00 | 100,00 | |

HPLC chromatogram of (*rac*)-**2d**:

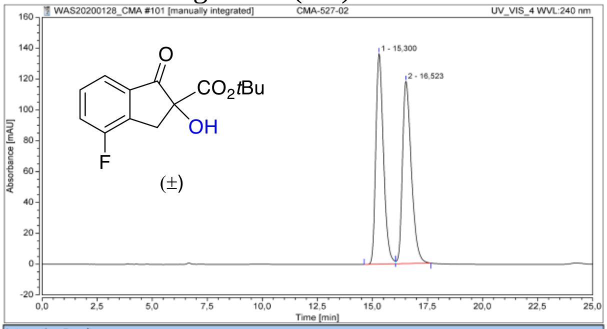


| Integration Results | | | | | | | |
|---|---|---|---|---|---|---|---|
| No. | Peak Name | Retention Time min | Area mAU*min | Height mAU | Relative Area % | Relative Height % | Amount n.a. |
| 1 | | 15,300 | 55,024 | 136,547 | 49,90 | 53,60 | n.a. |
| 2 | | 16,523 | 55,255 | 118,189 | 50,10 | 46,40 | n.a. |
| Total: | | | 110,279 | 254,735 | 100,00 | 100,00 | |

HPLC chromatogram of (*rac*)-**3d**:

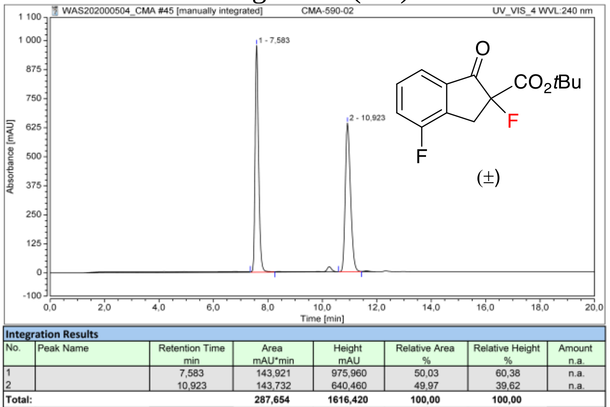


| Integration Results | | | | | | | |
|---|---|---|---|---|---|---|---|
| No. | Peak Name | Retention Time min | Area mAU*min | Height mAU | Relative Area % | Relative Height % | Amount n.a. |
| 1 | | 7,583 | 143,921 | 975,960 | 50,03 | 60,38 | n.a. |
| 2 | | 10,923 | 143,732 | 640,460 | 49,97 | 39,62 | n.a. |
| Total: | | | 287,654 | 1616,420 | 100,00 | 100,00 | |

HPLC chromatogram of enantioenriched **2d**:

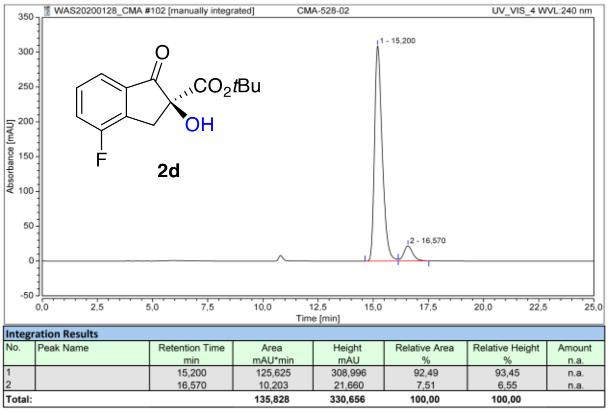


| Integration Results | | | | | | | |
|---|---|---|---|---|---|---|---|
| No. | Peak Name | Retention Time min | Area mAU*min | Height mAU | Relative Area % | Relative Height % | Amount n.a. |
| 1 | | 15,200 | 125,625 | 308,996 | 92,49 | 93,45 | n.a. |
| 2 | | 16,570 | 10,203 | 21,660 | 7,51 | 6,55 | n.a. |
| Total: | | | 135,828 | 330,656 | 100,00 | 100,00 | |

HPLC chromatogram of enantioenriched **3d**:

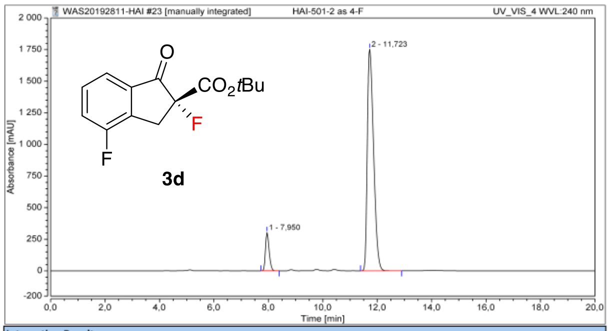


| Integration Results | | | | | | | |
|---|---|---|---|---|---|---|---|
| No. | Peak Name | Retention Time min | Area mAU*min | Height mAU | Relative Area % | Relative Height % | Amount n.a. |
| 1 | | 7,950 | 46,892 | 303,785 | 9,12 | 14,77 | n.a. |
| 2 | | 11,723 | 467,503 | 1752,719 | 90,88 | 85,23 | n.a. |
| Total: | | | 514,395 | 2056,504 | 100,00 | 100,00 | |

## HPLC chromatogram of (*rac*)-**2e**:

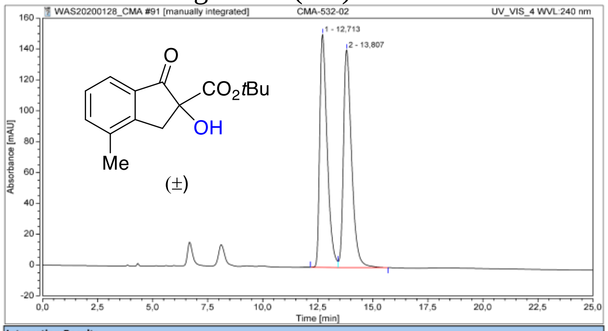


| Integration Results | | | | | | | |
|---|---|---|---|---|---|---|---|
| No. | Peak Name | Retention Time min | Area mAU*min | Height mAU | Relative Area % | Relative Height % | Amount n.a. |
| 1 | | 12,713 | 59,116 | 151,082 | 49,37 | 51,72 | n.a. |
| 2 | | 13,807 | 60,623 | 141,022 | 50,63 | 48,28 | n.a. |
| Total: | | | 119,740 | 292,103 | 100,00 | 100,00 | |

## HPLC chromatogram of (*rac*)-**3e**:

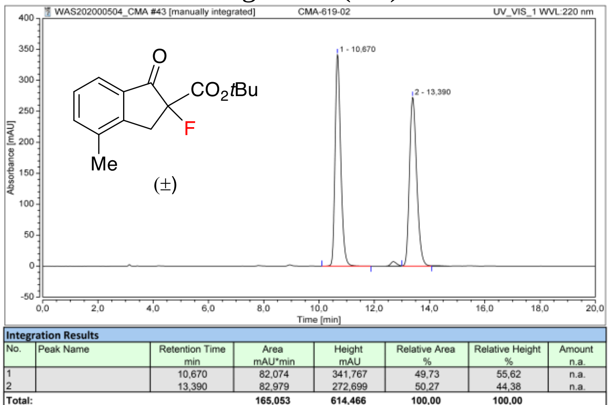


| Integration Results | | | | | | | |
|---|---|---|---|---|---|---|---|
| No. | Peak Name | Retention Time min | Area mAU*min | Height mAU | Relative Area % | Relative Height % | Amount n.a. |
| 1 | | 10,670 | 82,074 | 341,767 | 49,73 | 55,62 | n.a. |
| 2 | | 13,390 | 82,979 | 272,699 | 50,27 | 44,38 | n.a. |
| Total: | | | 165,053 | 614,466 | 100,00 | 100,00 | |

## HPLC chromatogram of enantioenriched **2e**:

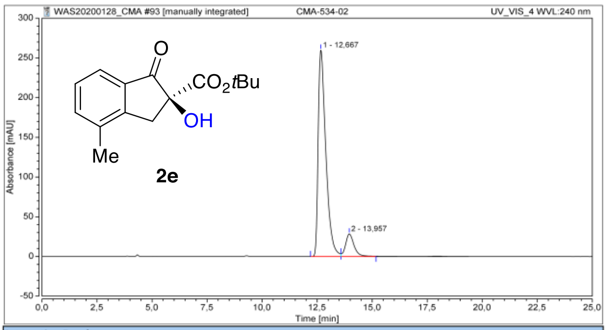


| Integration Results | | | | | | | |
|---|---|---|---|---|---|---|---|
| No. | Peak Name | Retention Time min | Area mAU*min | Height mAU | Relative Area % | Relative Height % | Amount n.a. |
| 1 | | 12,667 | 104,600 | 260,073 | 89,06 | 90,10 | n.a. |
| 2 | | 13,957 | 12,844 | 28,592 | 10,94 | 9,90 | n.a. |
| [illegible] | | | [illegible] | [illegible] | [illegible] | [illegible] | |

## HPLC chromatogram of enantioenriched **3e**:

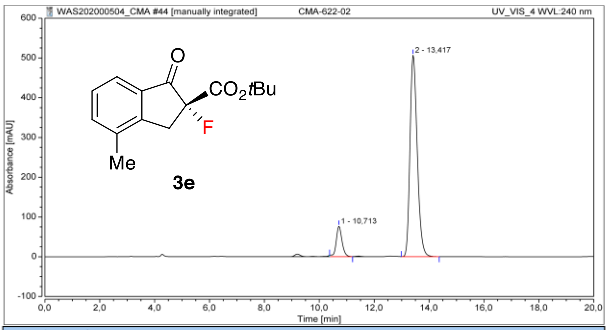


| Integration Results | | | | | | | |
|---|---|---|---|---|---|---|---|
| No. | Peak Name | Retention Time min | Area mAU*min | Height mAU | Relative Area % | Relative Height % | Amount n.a. |
| 1 | | 10,713 | 18,625 | 76,891 | 10,79 | 13,18 | n.a. |
| 2 | | 13,417 | 154,011 | 506,706 | 89,21 | 86,82 | n.a. |
| [illegible] | | | [illegible] | [illegible] | [illegible] | [illegible] | |

---

## HPLC chromatogram of (*rac*)-**2f**:

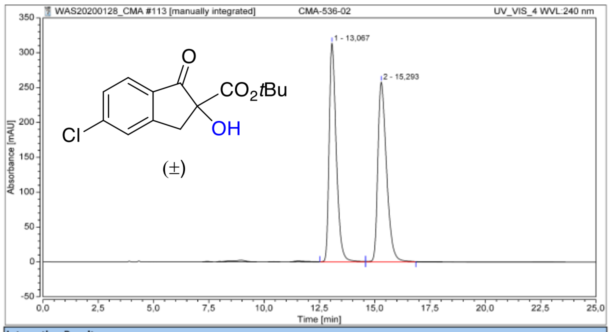


| Integration Results | | | | | | | |
|---|---|---|---|---|---|---|---|
| No. | Peak Name | Retention Time min | Area mAU*min | Height mAU | Relative Area % | Relative Height % | Amount n.a. |
| 1 | | 13,067 | 117,588 | 313,742 | 49,89 | 54,83 | n.a. |
| 2 | | 15,293 | 118,123 | 258,434 | 50,11 | 45,17 | n.a. |
| Total: | | | 235,711 | 572,176 | 100,00 | 100,00 | |

## HPLC chromatogram of (*rac*)-**3f**

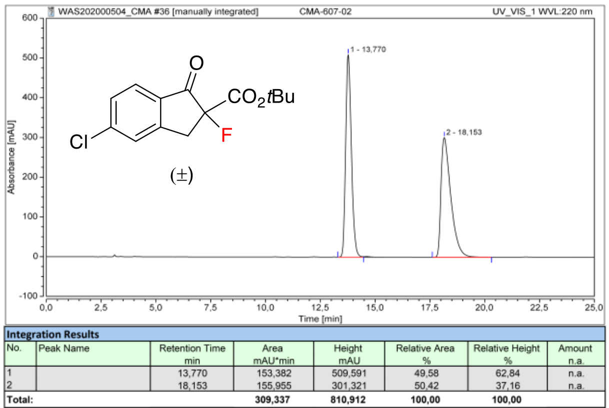


| Integration Results | | | | | | | |
|---|---|---|---|---|---|---|---|
| No. | Peak Name | Retention Time min | Area mAU*min | Height mAU | Relative Area % | Relative Height % | Amount n.a. |
| 1 | | 13,770 | 153,382 | 509,591 | 49,58 | 62,84 | n.a. |
| 2 | | 18,153 | 155,955 | 301,321 | 50,42 | 37,16 | n.a. |
| Total: | | | 309,337 | 810,912 | 100,00 | 100,00 | |

## HPLC chromatogram of enantioenriched **2f**:

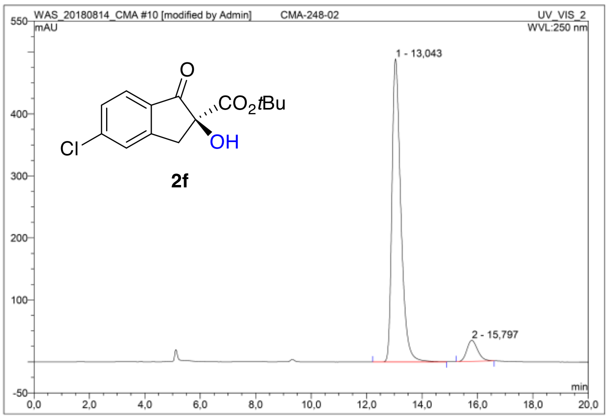


| No. | Ret.Time min | Peak Name | Height mAU | Area mAU*min | Rel.Area % | Amount | Type |
|---|---|---|---|---|---|---|---|
| 1 | 13,04 | n.a. | 488,885 | 181,909 | 92,29 | n.a. | BM * |
| 2 | 15,80 | n.a. | 33,769 | 15,207 | 7,71 | n.a. | BMB* |
| Total: | | | 522,654 | 197,116 | 100,00 | 0,000 | |

## HPLC chromatogram of enantioenriched **3f**:

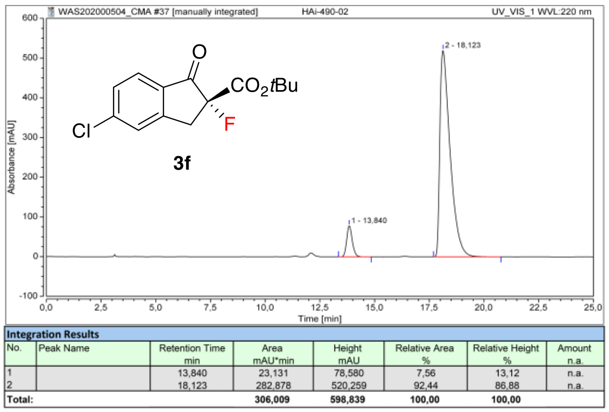


| Integration Results | | | | | | | |
|---|---|---|---|---|---|---|---|
| No. | Peak Name | Retention Time min | Area mAU*min | Height mAU | Relative Area % | Relative Height % | Amount n.a. |
| 1 | | 13,840 | 23,131 | 78,580 | 7,56 | 13,12 | n.a. |
| 2 | | 18,123 | 282,878 | 520,259 | 92,44 | 86,88 | n.a. |
| Total: | | | 306,009 | 598,839 | 100,00 | 100,00 | |

## HPLC chromatogram of (*rac*)-**2g**:

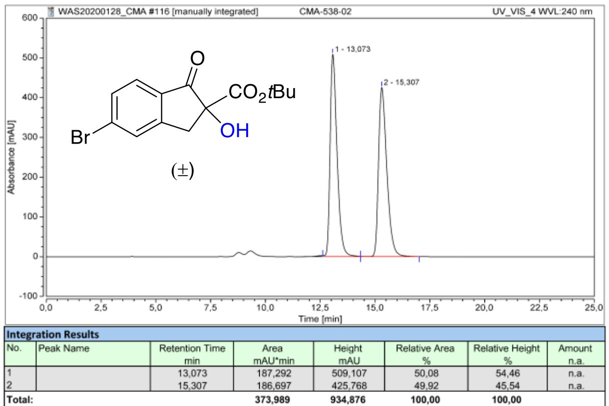


| No. | Peak Name | Retention Time min | Area mAU*min | Height mAU | Relative Area % | Relative Height % | Amount n.a. |
|---|---|---|---|---|---|---|---|
| 1 | | 13,073 | 187,292 | 509,107 | 50,08 | 54,46 | n.a. |
| 2 | | 15,307 | 186,697 | 425,768 | 49,92 | 45,54 | n.a. |
| Total: | | | 373,989 | 934,876 | 100,00 | 100,00 | |

## HPLC chromatogram of (*rac*)-**3g**

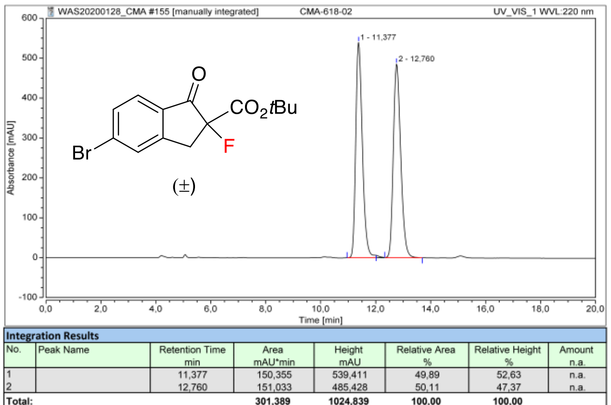


| No. | Peak Name | Retention Time min | Area mAU*min | Height mAU | Relative Area % | Relative Height % | Amount n.a. |
|---|---|---|---|---|---|---|---|
| 1 | | 11,377 | 150,355 | 539,411 | 49,89 | 52,63 | n.a. |
| 2 | | 12,760 | 151,033 | 485,428 | 50,11 | 47,37 | n.a. |
| Total: | | | 301,389 | 1024,839 | 100,00 | 100,00 | |

## HPLC chromatogram of enantioenriched **2g**:

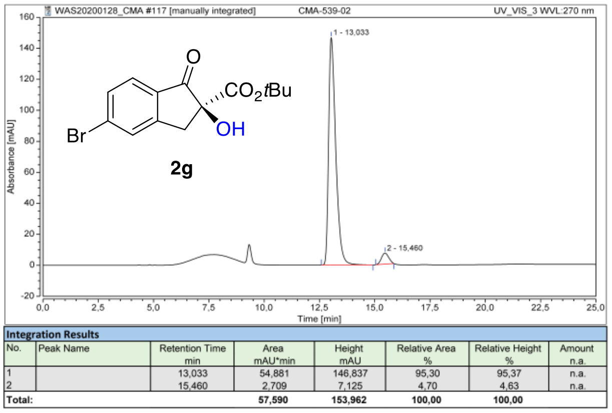


| No. | Peak Name | Retention Time min | Area mAU*min | Height mAU | Relative Area % | Relative Height % | Amount n.a. |
|---|---|---|---|---|---|---|---|
| 1 | | 13,033 | 54,881 | 146,837 | 95,30 | 95,37 | n.a. |
| 2 | | 15,460 | 2,709 | 7,125 | 4,70 | 4,63 | n.a. |
| Total: | | | 57,590 | 153,962 | 100,00 | 100,00 | |

## HPLC chromatogram of enantioenriched **3g**:

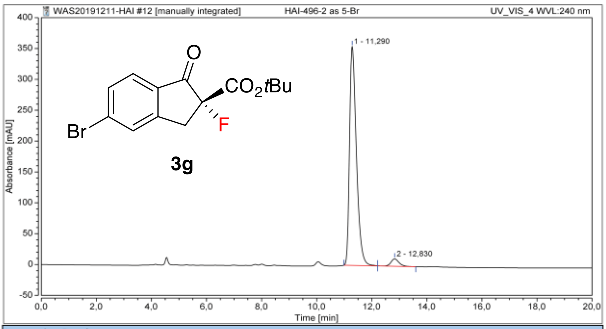


| No. | Peak Name | Retention Time min | Area mAU*min | Height mAU | Relative Area % | Relative Height % | Amount n.a. |
|---|---|---|---|---|---|---|---|
| 1 | | 11,290 | 102,179 | 354,780 | 96,18 | 96,74 | n.a. |
| 2 | | 12,830 | 4,053 | 11,960 | 3,82 | 3,26 | n.a. |
| Total: | | | 106,232 | 366,740 | 100,00 | 100,00 | |

## HPLC chromatogram of (*rac*)-**2h**:

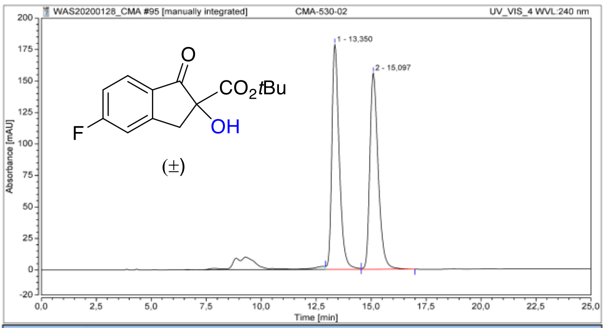


| No. | Peak Name | Retention Time min | Area mAU*min | Height mAU | Relative Area % | Relative Height % | Amount n.a. |
|---|---|---|---|---|---|---|---|
| 1 | | 13,350 | 69,566 | 178,840 | 50,23 | 53,41 | n.a. |
| 2 | | 15,097 | 68,938 | 156,002 | 49,77 | 46,59 | n.a. |
| Total: | | | 138,504 | 334,841 | 100,00 | 100,00 | |

## HPLC chromatogram of (*rac*)-**3h**

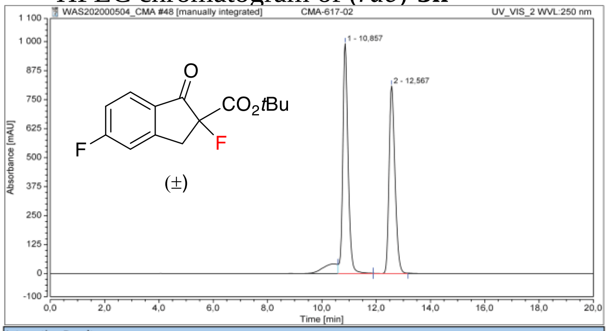


| No. | Peak Name | Retention Time min | Area mAU*min | Height mAU | Relative Area % | Relative Height % | Amount n.a. |
|---|---|---|---|---|---|---|---|
| 1 | | 10,857 | 223,344 | 991,753 | 52,10 | 55,07 | n.a. |
| 2 | | 12,567 | 205,345 | 809,052 | 47,90 | 44,93 | n.a. |
| Total: | | | 428,688 | 1800,806 | 100,00 | 100,00 | |

## HPLC chromatogram of enantioenriched **2h**:

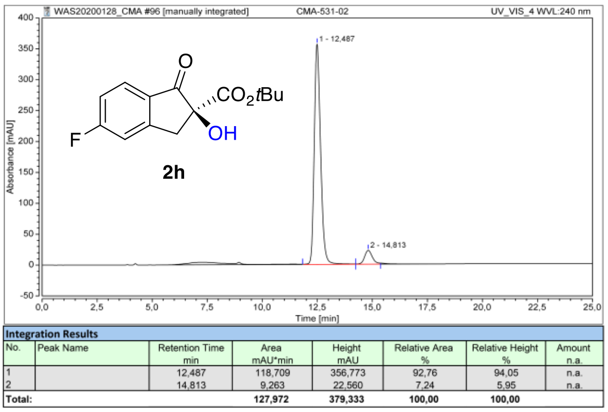


| No. | Peak Name | Retention Time min | Area mAU*min | Height mAU | Relative Area % | Relative Height % | Amount n.a. |
|---|---|---|---|---|---|---|---|
| 1 | | 12,487 | 118,709 | 356,773 | 92,76 | 94,05 | n.a. |
| 2 | | 14,813 | 9,263 | 22,560 | 7,24 | 5,95 | n.a. |
| Total: | | | 127,972 | 379,333 | 100,00 | 100,00 | |

## HPLC chromatogram of enantioenriched **3h**:

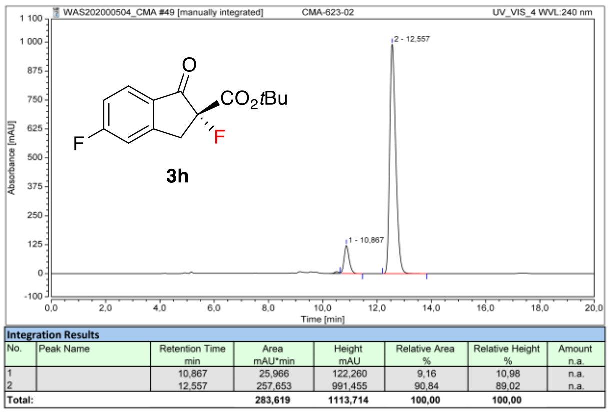


| No. | Peak Name | Retention Time min | Area mAU*min | Height mAU | Relative Area % | Relative Height % | Amount n.a. |
|---|---|---|---|---|---|---|---|
| 1 | | 10,867 | 25,966 | 122,260 | 9,16 | 10,98 | n.a. |
| 2 | | 12,557 | 257,653 | 991,455 | 90,84 | 89,02 | n.a. |
| Total: | | | 283,619 | 1113,714 | 100,00 | 100,00 | |

## HPLC chromatogram of (*rac*)-**2i**:

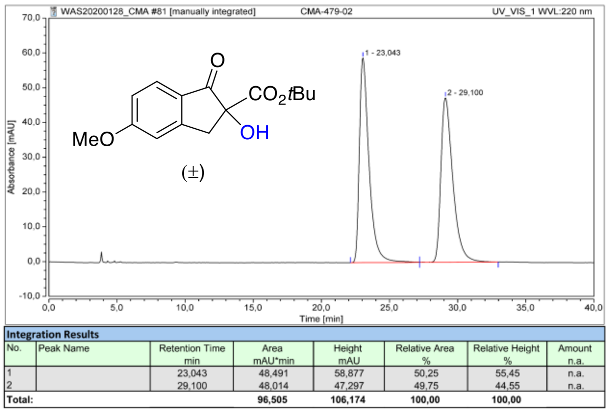


| Integration Results | | | | | | | |
|---|---|---|---|---|---|---|---|
| No. | Peak Name | Retention Time min | Area mAU*min | Height mAU | Relative Area % | Relative Height % | Amount n.a. |
| 1 | | 23,043 | 48,491 | 58,877 | 50,25 | 55,45 | n.a. |
| 2 | | 29,100 | 48,014 | 47,297 | 49,75 | 44,55 | n.a. |
| Total: | | | 96,505 | 106,174 | 100,00 | 100,00 | |

## HPLC chromatogram of (*rac*)-**3i**:

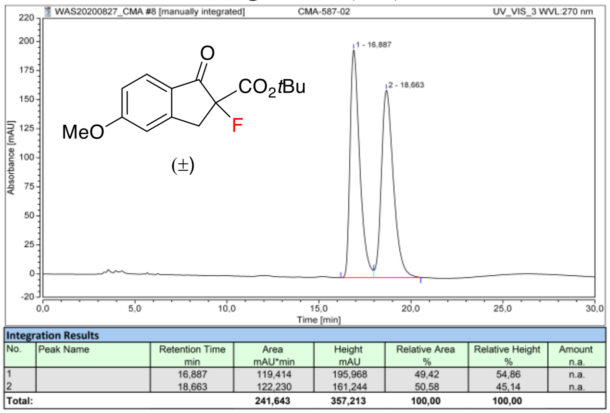


| Integration Results | | | | | | | |
|---|---|---|---|---|---|---|---|
| No. | Peak Name | Retention Time min | Area mAU*min | Height mAU | Relative Area % | Relative Height % | Amount n.a. |
| 1 | | 16,887 | 119,414 | 195,968 | 49,42 | 54,86 | n.a. |
| 2 | | 18,663 | 122,230 | 161,244 | 50,58 | 45,14 | n.a. |
| Total: | | | 241,643 | 357,213 | 100,00 | 100,00 | |

## HPLC chromatogram of enantioenriched **2i**:

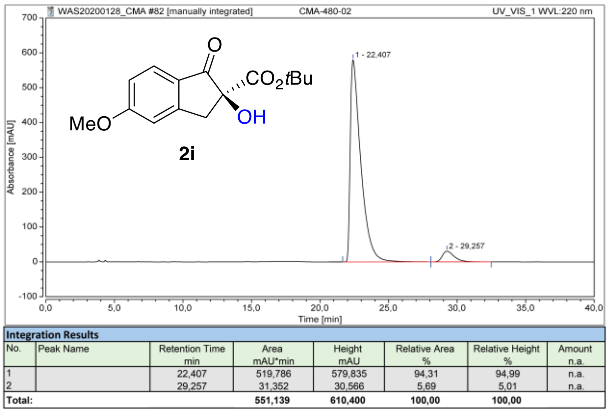


| Integration Results | | | | | | | |
|---|---|---|---|---|---|---|---|
| No. | Peak Name | Retention Time min | Area mAU*min | Height mAU | Relative Area % | Relative Height % | Amount n.a. |
| 1 | | 22,407 | 519,786 | 579,835 | 94,31 | 94,99 | n.a. |
| 2 | | 29,257 | 31,352 | 30,566 | 5,69 | 5,01 | n.a. |
| Total: | | | 551,139 | 610,400 | 100,00 | 100,00 | |

## HPLC chromatogram of enantioenriched **3i**:

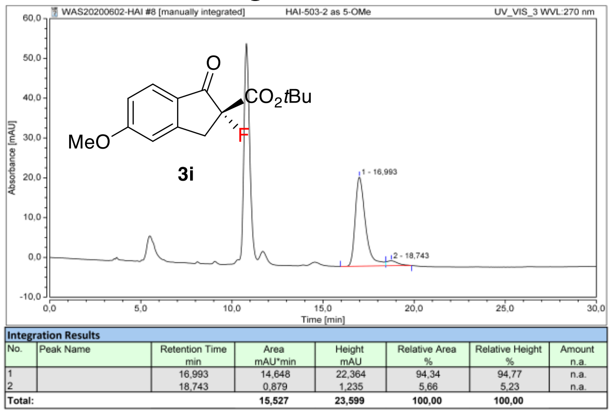


| Integration Results | | | | | | | |
|---|---|---|---|---|---|---|---|
| No. | Peak Name | Retention Time min | Area mAU*min | Height mAU | Relative Area % | Relative Height % | Amount n.a. |
| 1 | | 16,993 | 14,648 | 22,364 | 94,34 | 94,77 | n.a. |
| 2 | | 18,743 | 0,879 | 1,235 | 5,66 | 5,23 | n.a. |
| Total: | | | 15,527 | 23,599 | 100,00 | 100,00 | |

## HPLC chromatogram of (*rac*)-**2j**:

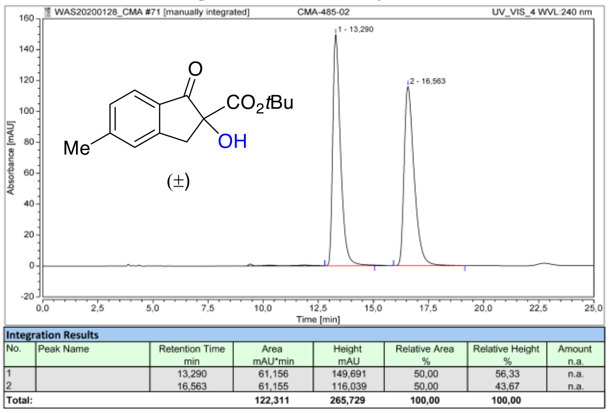


| Integration Results | | | | | | | |
|---|---|---|---|---|---|---|---|
| No. | Peak Name | Retention Time min | Area mAU*min | Height mAU | Relative Area % | Relative Height % | Amount n.a. |
| 1 | | 13,290 | 61,156 | 149,691 | 50,00 | 56,33 | n.a. |
| 2 | | 16,563 | 61,155 | 116,039 | 50,00 | 43,67 | n.a. |
| Total: | | | 122,311 | 265,729 | 100,00 | 100,00 | |

## HPLC chromatogram of (*rac*)-**3j**:

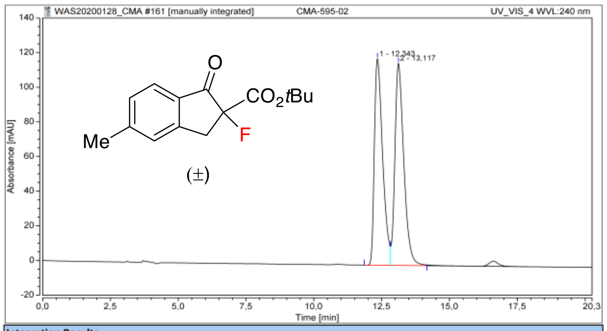


| Integration Results | | | | | | | |
|---|---|---|---|---|---|---|---|
| No. | Peak Name | Retention Time min | Area mAU*min | Height mAU | Relative Area % | Relative Height % | Amount n.a. |
| 1 | | 12,343 | 42,137 | 119,209 | 48,65 | 50,52 | n.a. |
| 2 | | 13,117 | 44,482 | 116,746 | 51,35 | 49,48 | n.a. |
| Total: | | | 86,619 | 235,955 | 100,00 | 100,00 | |

## HPLC chromatogram of enantioenriched **2j**:

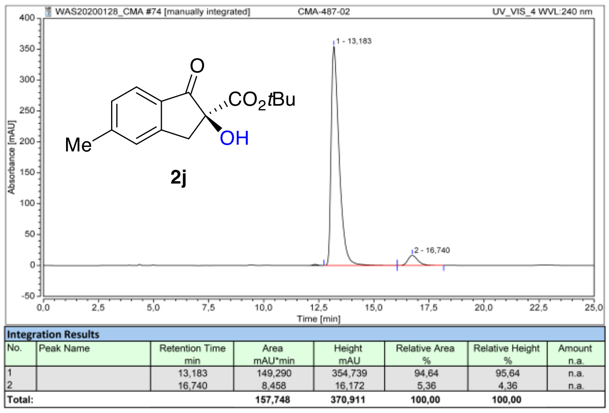


| Integration Results | | | | | | | |
|---|---|---|---|---|---|---|---|
| No. | Peak Name | Retention Time min | Area mAU*min | Height mAU | Relative Area % | Relative Height % | Amount n.a. |
| 1 | | 13,183 | 149,290 | 354,739 | 94,64 | 95,64 | n.a. |
| 2 | | 16,740 | 8,458 | 16,172 | 5,36 | 4,36 | n.a. |
| Total: | | | 157,748 | 370,911 | 100,00 | 100,00 | |

## HPLC chromatogram of enantioenriched **3j**:

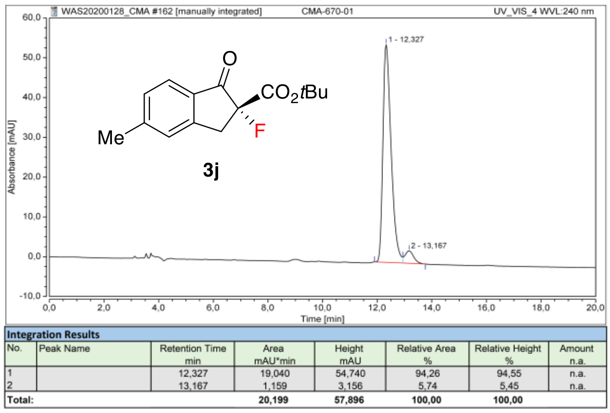


| Integration Results | | | | | | | |
|---|---|---|---|---|---|---|---|
| No. | Peak Name | Retention Time min | Area mAU*min | Height mAU | Relative Area % | Relative Height % | Amount n.a. |
| 1 | | 12,327 | 19,040 | 54,740 | 94,26 | 94,55 | n.a. |
| 2 | | 13,167 | 1,159 | 3,156 | 5,74 | 5,45 | n.a. |
| Total: | | | 20,199 | 57,896 | 100,00 | 100,00 | |

## HPLC chromatogram of (*rac*)-**2k**:

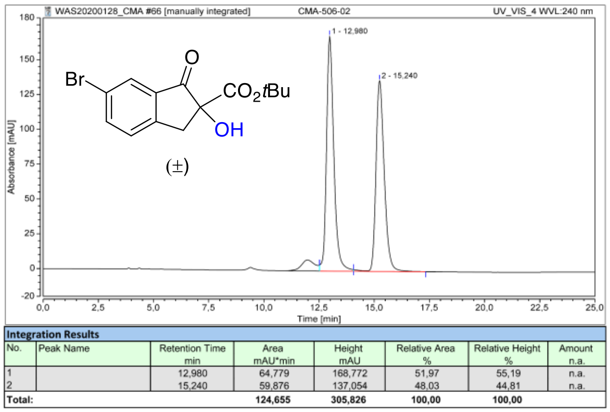


| Integration Results | | | | | | | |
|---|---|---|---|---|---|---|---|
| No. | Peak Name | Retention Time min | Area mAU*min | Height mAU | Relative Area % | Relative Height % | Amount n.a. |
| 1 | | 12,980 | 64,779 | 168,772 | 51,97 | 55,19 | n.a. |
| 2 | | 15,240 | 59,876 | 137,054 | 48,03 | 44,81 | n.a. |
| Total: | | | 124,655 | 305,826 | 100,00 | 100,00 | |

## HPLC chromatogram of (*rac*)-**3k**:

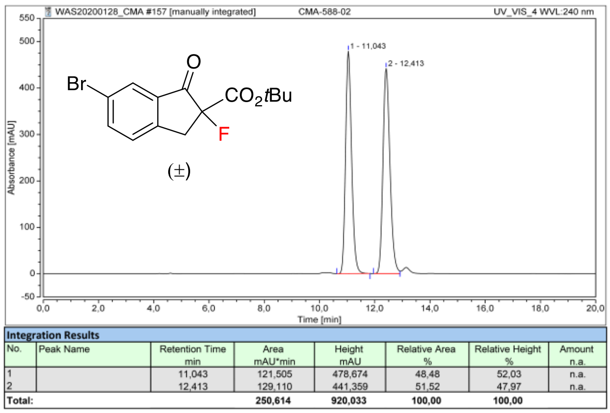


| Integration Results | | | | | | | |
|---|---|---|---|---|---|---|---|
| No. | Peak Name | Retention Time min | Area mAU*min | Height mAU | Relative Area % | Relative Height % | Amount n.a. |
| 1 | | 11,043 | 121,505 | 478,674 | 48,48 | 52,03 | n.a. |
| 2 | | 12,413 | 129,110 | 441,359 | 51,52 | 47,97 | n.a. |
| Total: | | | 250,614 | 920,033 | 100,00 | 100,00 | |

## HPLC chromatogram of enantioenriched **2k**:

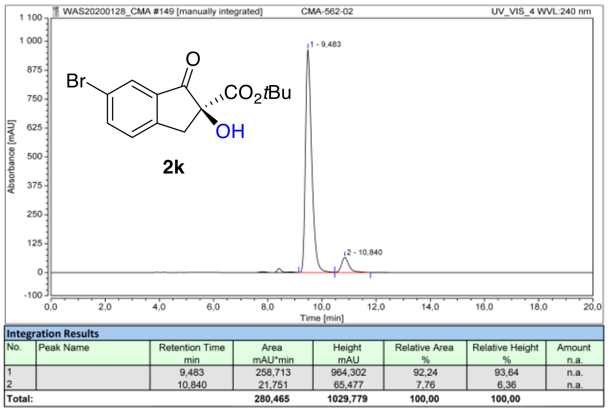


| Integration Results | | | | | | | |
|---|---|---|---|---|---|---|---|
| No. | Peak Name | Retention Time min | Area mAU*min | Height mAU | Relative Area % | Relative Height % | Amount n.a. |
| 1 | | 9,483 | 258,713 | 964,302 | 92,24 | 93,64 | n.a. |
| 2 | | 10,840 | 21,751 | 65,477 | 7,76 | 6,36 | n.a. |
| Total: | | | 280,465 | 1029,779 | 100,00 | 100,00 | |

## HPLC chromatogram of enantioenriched **3k**:

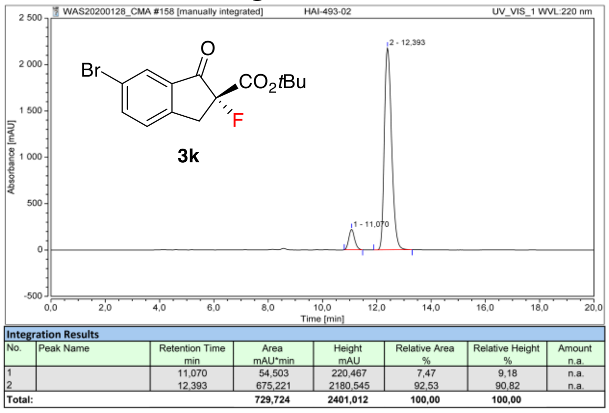


| Integration Results | | | | | | | |
|---|---|---|---|---|---|---|---|
| No. | Peak Name | Retention Time min | Area mAU*min | Height mAU | Relative Area % | Relative Height % | Amount n.a. |
| 1 | | 11,070 | 54,503 | 220,467 | 7,47 | 9,18 | n.a. |
| 2 | | 12,393 | 675,221 | 2180,545 | 92,53 | 90,82 | n.a. |
| Total: | | | 729,724 | 2401,012 | 100,00 | 100,00 | |

## HPLC chromatogram of (*rac*)-**2l**:

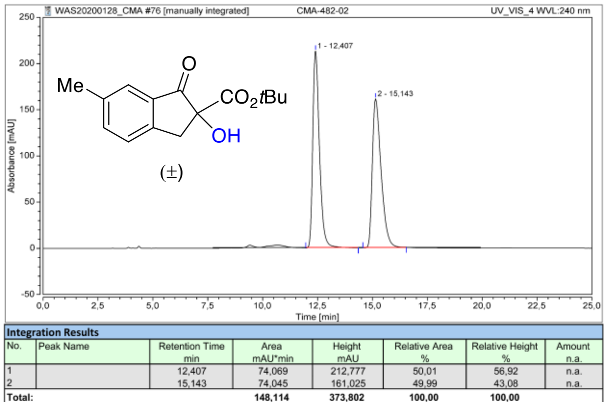


| Integration Results | | | | | | | |
|---|---|---|---|---|---|---|---|
| No. | Peak Name | Retention Time min | Area mAU*min | Height mAU | Relative Area % | Relative Height % | Amount n.a. |
| 1 | | 12,407 | 74,069 | 212,777 | 50,01 | 56,92 | n.a. |
| 2 | | 15,143 | 74,045 | 161,025 | 49,99 | 43,08 | n.a. |
| Total: | | | 148,114 | 373,802 | 100,00 | 100,00 | |

## HPLC chromatogram of (*rac*)-**3l**:

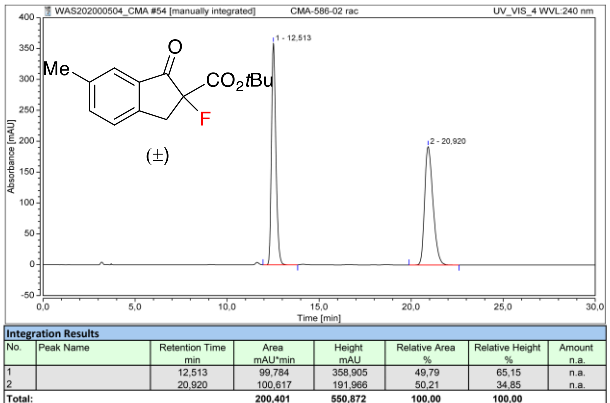


| Integration Results | | | | | | | |
|---|---|---|---|---|---|---|---|
| No. | Peak Name | Retention Time min | Area mAU*min | Height mAU | Relative Area % | Relative Height % | Amount n.a. |
| 1 | | 12,513 | 99,784 | 358,905 | 49,79 | 65,15 | n.a. |
| 2 | | 20,920 | 100,617 | 191,966 | 50,21 | 34,85 | n.a. |
| Total: | | | 200,401 | 550,872 | 100,00 | 100,00 | |

## HPLC chromatogram of enantioenriched **2l**:

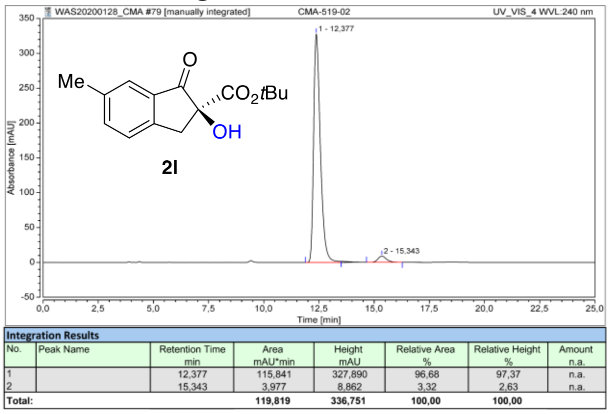


| Integration Results | | | | | | | |
|---|---|---|---|---|---|---|---|
| No. | Peak Name | Retention Time min | Area mAU*min | Height mAU | Relative Area % | Relative Height % | Amount n.a. |
| 1 | | 12,377 | 115,841 | 327,890 | 96,68 | 97,37 | n.a. |
| 2 | | 15,343 | 3,977 | 8,862 | 3,32 | 2,63 | n.a. |
| Total: | | | 119,819 | 336,751 | 100,00 | 100,00 | |

## HPLC chromatogram of enantioenriched **3l**:

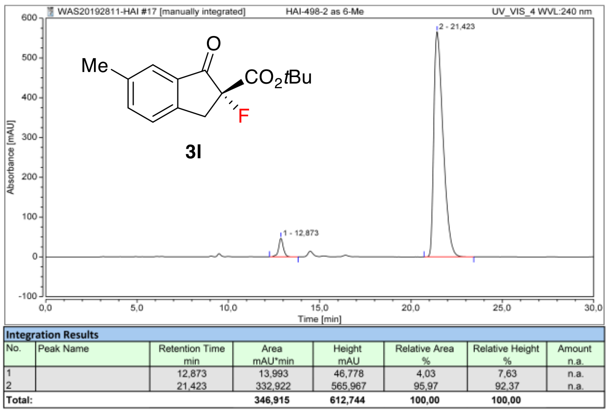


| Integration Results | | | | | | | |
|---|---|---|---|---|---|---|---|
| No. | Peak Name | Retention Time min | Area mAU*min | Height mAU | Relative Area % | Relative Height % | Amount n.a. |
| 1 | | 12,873 | 13,993 | 46,778 | 4,03 | 7,63 | n.a. |
| 2 | | 21,423 | 332,922 | 565,967 | 95,97 | 92,37 | n.a. |
| Total: | | | 346,915 | 612,744 | 100,00 | 100,00 | |

## HPLC chromatogram of (*rac*)-**2m**:

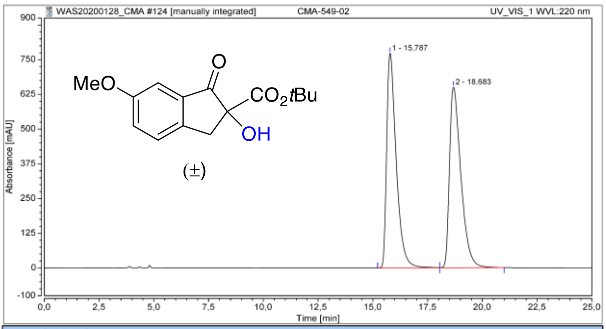


| Integration Results | | | | | | | |
|---|---|---|---|---|---|---|---|
| No. | Peak Name | Retention Time min | Area mAU*min | Height mAU | Relative Area % | Relative Height % | Amount n.a. |
| 1 | | 15,787 | 390,722 | 773,912 | 50,00 | 54,29 | n.a. |
| 2 | | 18,683 | 390,754 | 651,506 | 50,00 | 45,71 | n.a. |
| **Total:** | | | **781,476** | **1425,419** | **100,00** | **100,00** | |

## HPLC chromatogram of (*rac*)-**3m**:

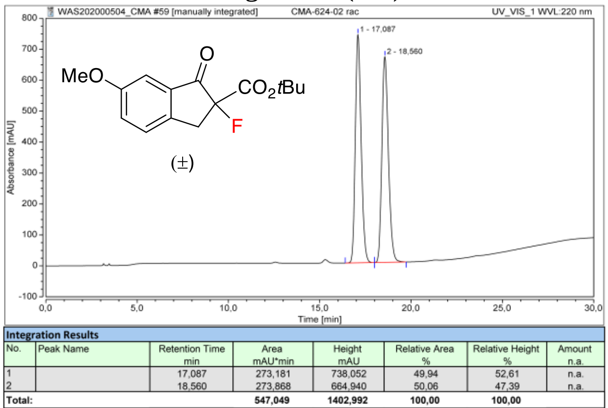


| Integration Results | | | | | | | |
|---|---|---|---|---|---|---|---|
| No. | Peak Name | Retention Time min | Area mAU*min | Height mAU | Relative Area % | Relative Height % | Amount n.a. |
| 1 | | 17,087 | 273,181 | 738,052 | 49,94 | 52,61 | n.a. |
| 2 | | 18,560 | 273,868 | 664,940 | 50,06 | 47,39 | n.a. |
| **Total:** | | | **547,049** | **1402,992** | **100,00** | **100,00** | |

## HPLC chromatogram of enantioenriched **2m**:

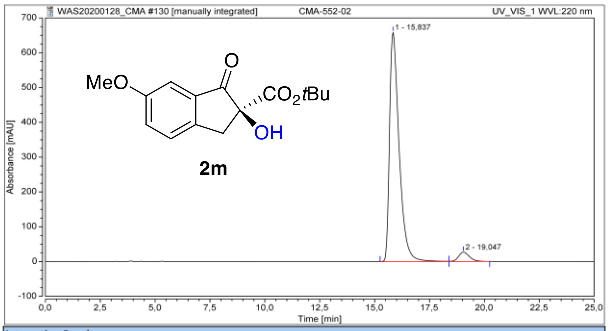


| Integration Results | | | | | | | |
|---|---|---|---|---|---|---|---|
| No. | Peak Name | Retention Time min | Area mAU*min | Height mAU | Relative Area % | Relative Height % | Amount n.a. |
| 1 | | 15,837 | 331,718 | 658,813 | 95,57 | 96,09 | n.a. |
| 2 | | 19,047 | 15,379 | 26,803 | 4,43 | 3,91 | n.a. |
| **Total:** | | | **347,097** | **685,617** | **100,00** | **100,00** | |

## HPLC chromatogram of enantioenriched **3m**:

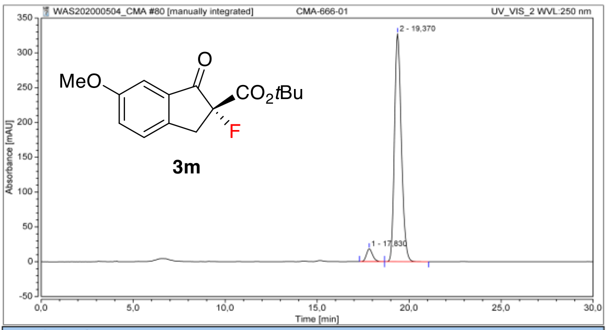


| Integration Results | | | | | | | |
|---|---|---|---|---|---|---|---|
| No. | Peak Name | Retention Time min | Area mAU*min | Height mAU | Relative Area % | Relative Height % | Amount n.a. |
| 1 | | 17,830 | 7,091 | 18,385 | 4,77 | 5,32 | n.a. |
| 2 | | 19,370 | 141,601 | 327,399 | 95,23 | 94,68 | n.a. |
| **Total:** | | | **148,692** | **345,784** | **100,00** | **100,00** | |

## HPLC chromatogram of enantioenriched **2n**:

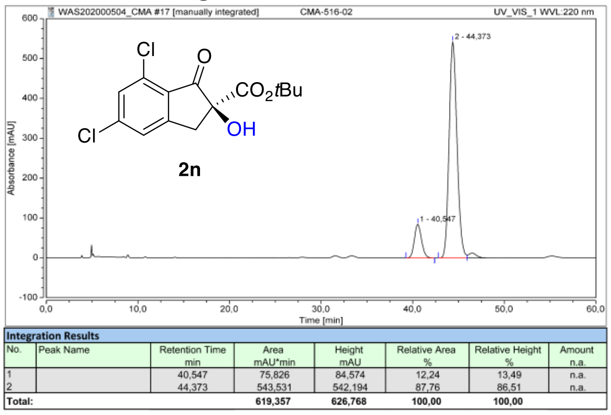


| Integration Results | | | | | | | |
|---|---|---|---|---|---|---|---|
| No. | Peak Name | Retention Time min | Area mAU*min | Height mAU | Relative Area % | Relative Height % | Amount n.a. |
| 1 | | 40,547 | 75,826 | 84,574 | 12,24 | 13,49 | n.a. |
| 2 | | 44,373 | 543,531 | 542,194 | 87,76 | 86,51 | n.a. |
| **Total:** | | | **619,357** | **626,768** | **100,00** | **100,00** | |

## HPLC chromatogram of enantioenriched **3n**:

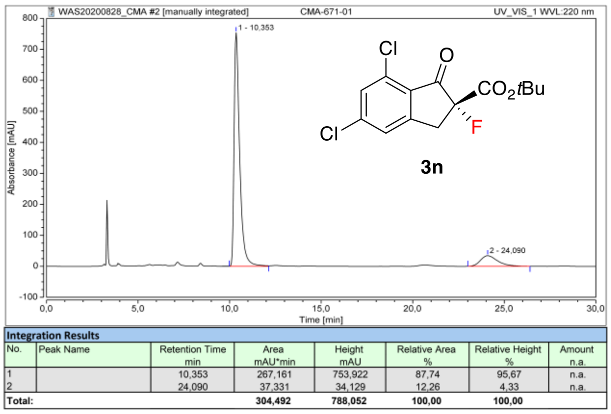


| Integration Results | | | | | | | |
|---|---|---|---|---|---|---|---|
| No. | Peak Name | Retention Time min | Area mAU*min | Height mAU | Relative Area % | Relative Height % | Amount n.a. |
| 1 | | 10,353 | 267,161 | 753,922 | 87,74 | 95,67 | n.a. |
| 2 | | 24,090 | 37,331 | 34,129 | 12,26 | 4,33 | n.a. |
| **Total:** | | | **304,492** | **788,052** | **100,00** | **100,00** | |

# 6. HRMS Data Report

(*R*)-**3a**. **HRMS** (ESI-Orbitrap, MeOH) *m/z*: $[M + NH_4]^+$ calcd for $C_{14}H_{19}FO_3N$, 268.1343; found, 268.1334.

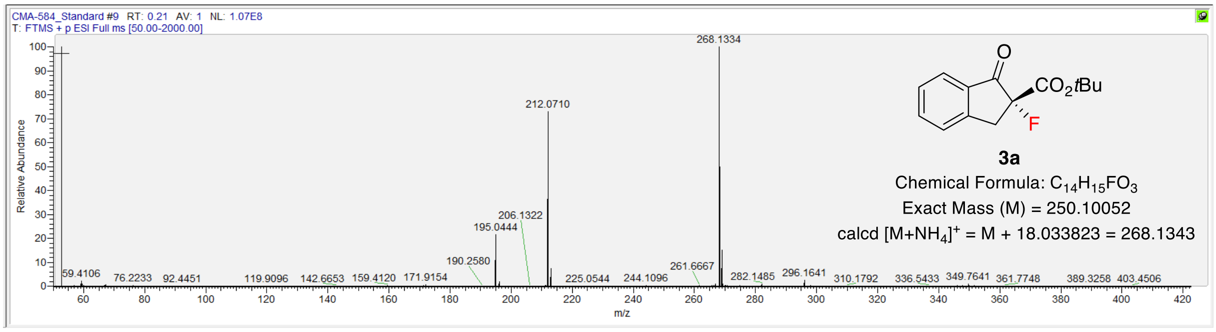


(*R*)-**3b**. **HRMS** (ESI-Orbitrap, MeOH) *m/z*: $[M + H]^+$ calcd for $C_{11}H_{10}FO_3$, 209.0608; found, 209.0604. $[M + NH_4]^+$ calcd for $C_{11}H_{14}FO_3N$, 226.0874; found, 226.0868.

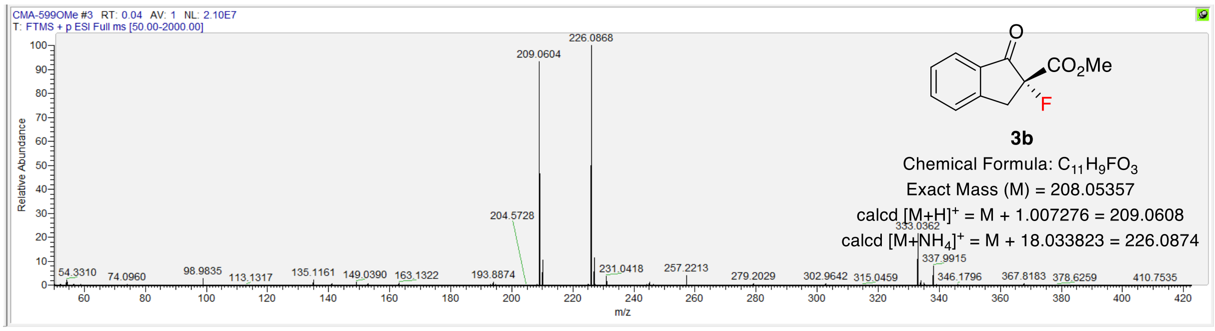


(*R*)-**3c**. **HRMS** (ESI-Orbitrap, MeOH) *m/z*: $[M + NH_4]^+$ calcd for $C_{20}H_{25}FNO_3N$, 346.1813; found, 346.1801.

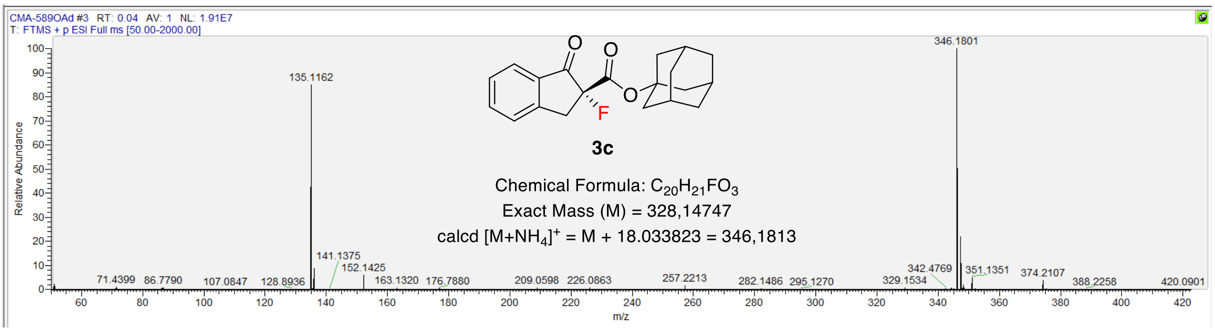


(*R*)-**3d**. **HRMS** (ESI-Orbitrap, MeOH) *m/z*: $[M + NH_4]^+$ calcd for $C_{14}H_{18}F_2O_3N$, 286.1249; found, 286.1238.

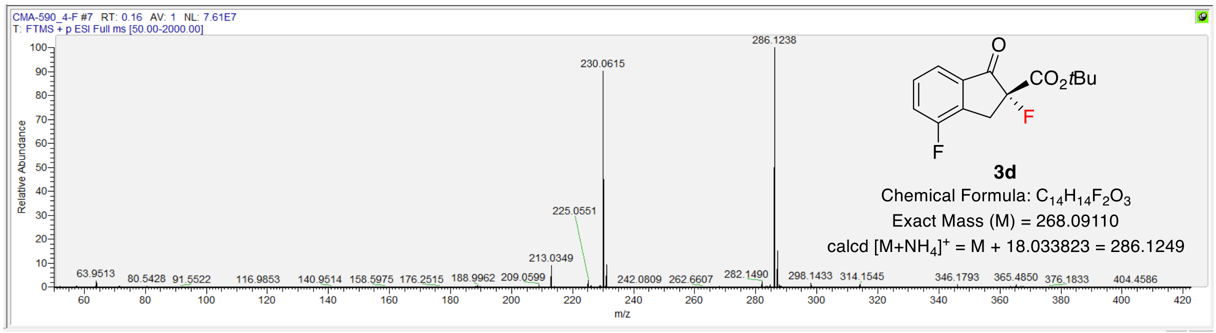

(*R*)-**3e**. **HRMS** (ESI-Orbitrap, MeOH) *m/z*: $[M + NH_4]^+$ calcd for $C_{15}H_{21}FO_3N$, 282.1500; found, 282.1490.

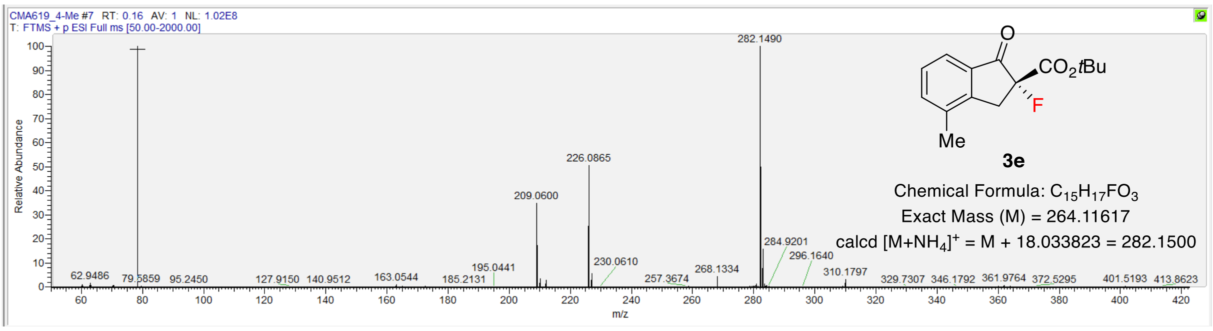


(*R*)-**3f**. **HRMS** (ESI-Orbitrap, MeOH) *m/z*: $[M + NH_4]^+$ calcd for $C_{14}H_{18}ClFO_3N$, 302.0954; found, 302.0942.

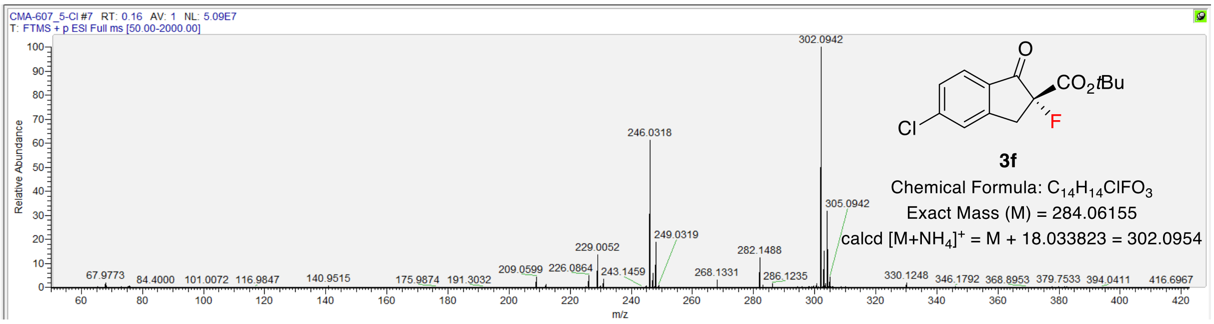


(*R*)-**3g**. **HRMS** (ESI-Orbitrap, MeOH) *m/z*: $[M + NH_4]^+$ calcd for $C_{14}H_{18}BrFO_3N$, 346.0449; found, 346.0436.

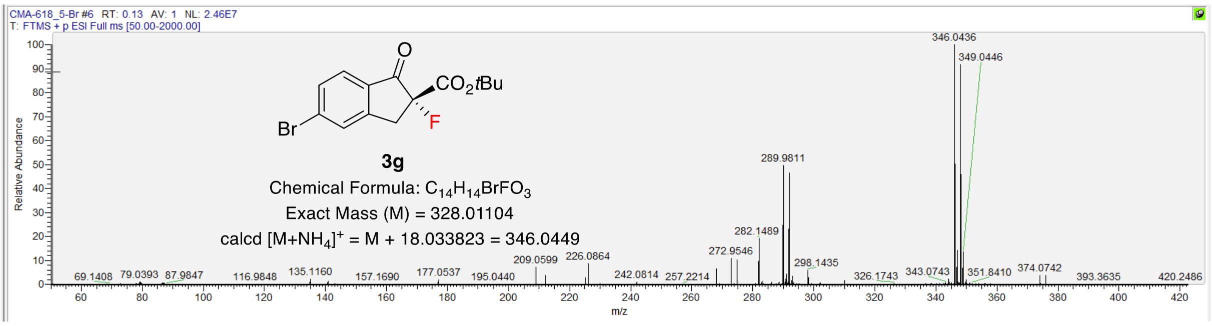


(*R*)-**3h**. **HRMS** (ESI-Orbitrap, MeOH) *m/z*: $[M + NH_4]^+$ calcd for $C_{14}H_{18}F_2O_3N$, 286.1249; found, 286.1239.

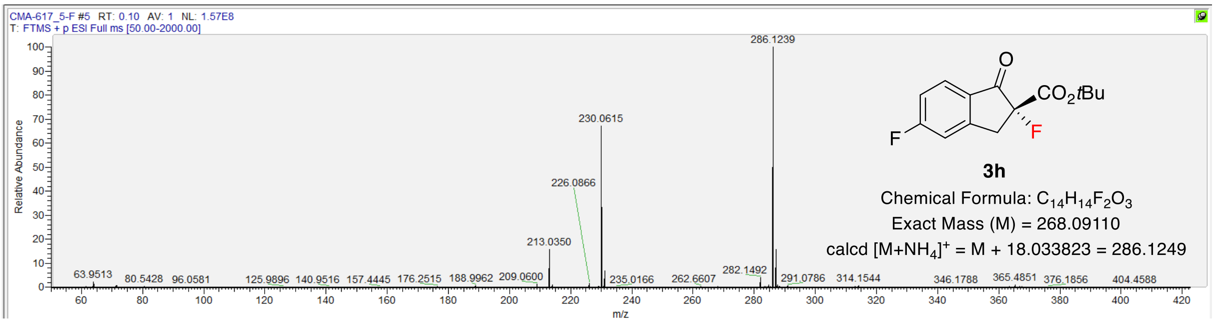

(*R*)-**3i**. **HRMS** (ESI-Orbitrap, MeOH) *m/z*: $[M + NH_4]^+$ calcd for $C_{15}H_{21}FO_4N$, 298.1449; found, 298.1437.

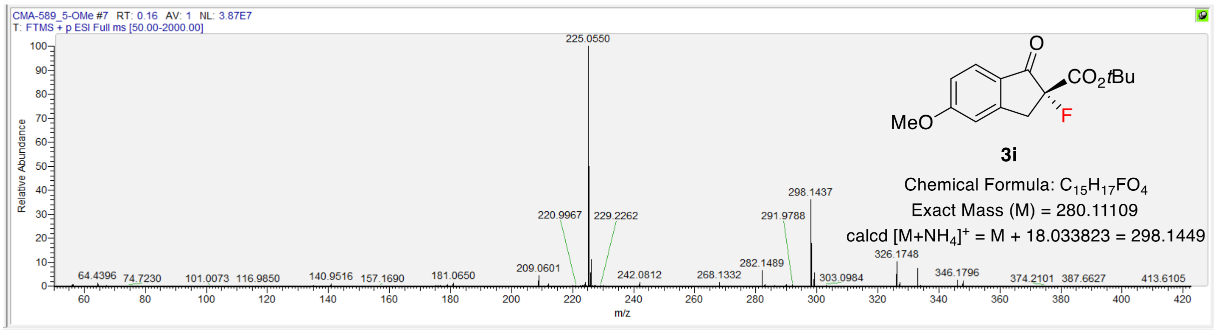


(*R*)-**3j**. **HRMS** (ESI-Orbitrap, MeOH) *m/z*: $[M + NH_4]^+$ calcd for $C_{15}H_{21}FO_3N$, 282.1500; found, 282.1492.

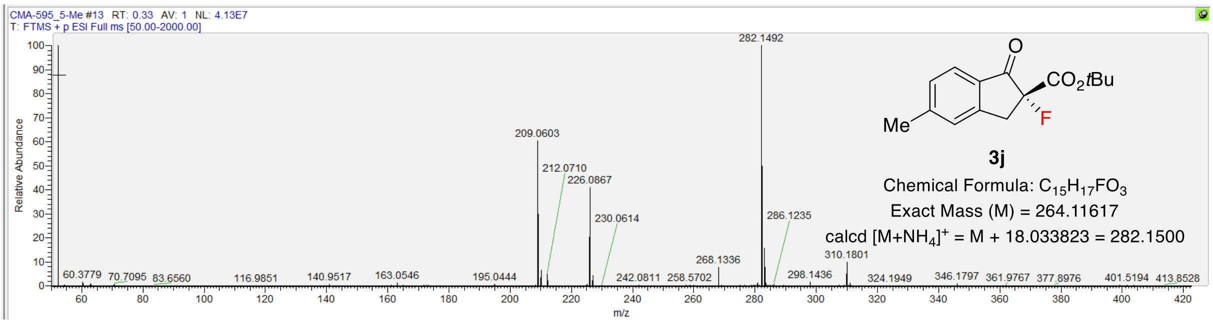


(*R*)-**3k**. **HRMS** (ESI-Orbitrap, MeOH) *m/z*: $[M + NH_4]^+$ calcd for $C_{14}H_{18}BrFO_3N$, 346.0449; found, 346.0453.

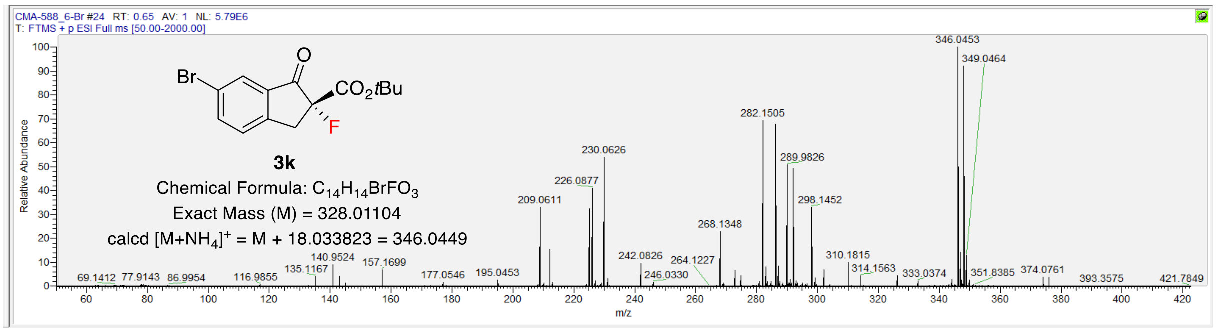


(*R*)-**3l**. **HRMS** (ESI-Orbitrap, MeOH) *m/z*: $[M + NH_4]^+$ calcd for $C_{15}H_{21}FO_3N$, 282.1500; found, 282.1491.

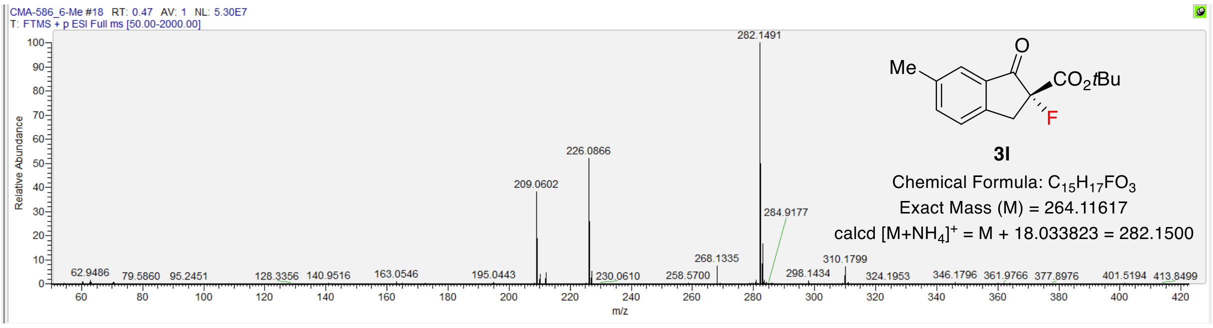

(*R*)-**3m**. **HRMS** (ESI-Orbitrap, MeOH) *m*/*z*: $[M + NH_4]^+$ calcd for $C_{15}H_{21}FO_4N$, 298.1449; found, 298.1439.

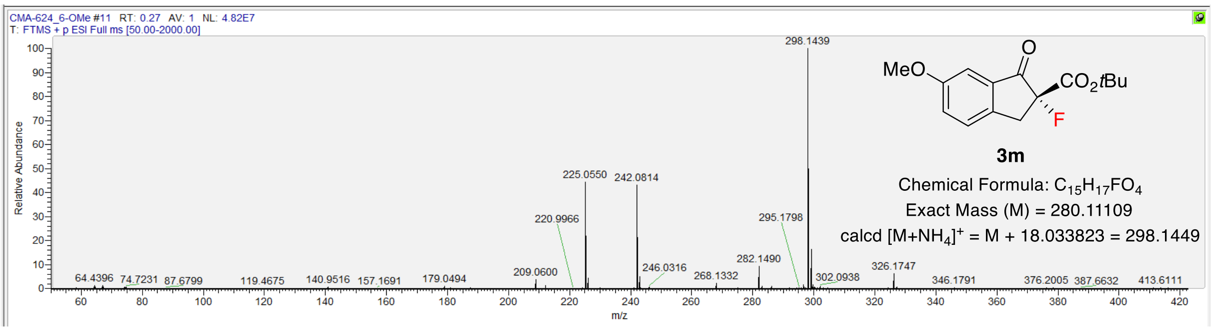


(*R*)-**3n**. **HRMS** (ESI-Orbitrap, MeOH) *m*/*z*: $[M + NH_4]^+$ calcd for $C_{14}H_{17}Cl_2FO_3N$, 336.0564; found, 336.0575.

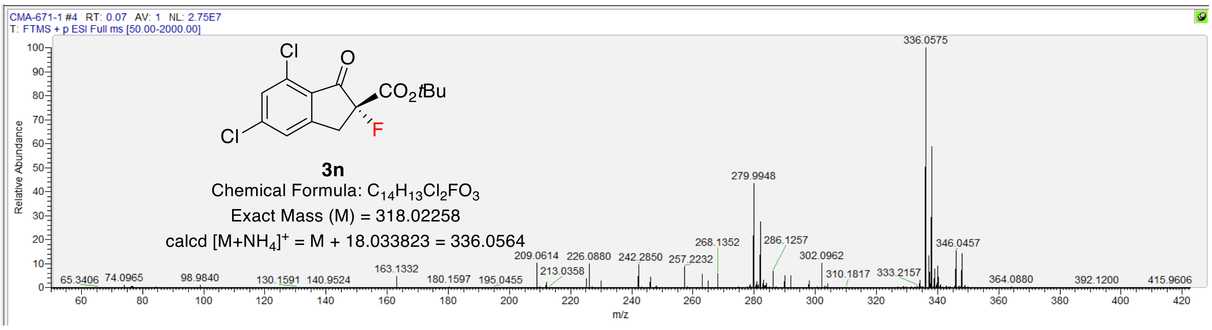

Supplement: SI [file EMS114212-supplement-SI.pdf]
